# Effects of Different Nutritional Strategies on the Prevention and Management of Gestational Diabetes Mellitus: A Systematic Review and Network Meta-Analysis

**DOI:** 10.3390/healthcare14162593

**Published:** 2026-08-18

**Authors:** Jing Shi, Ke Chen, Yingxue Tang, Xingzhe Ren, Yong Zhang

**Affiliations:** 1 School of Medicine, Shaoxing University, Shaoxing 312000, China; 24021054115@usx.edu.cn (J.S.); 24021054118@usx.edu.cn (Y.T.); 24021054114@usx.edu.cn (X.R.); 2 Department of Obstetrics, The First Affiliated Hospital of Shaoxing University, Shaoxing 312000, China; 17357507331@163.com

**Keywords:** perinatal complications, dietary pattern, fasting blood glucose, HOMA-IR

## Abstract

**Highlights:**

**What are the main findings?**

- Inositol supplementation was associated with reduced GDM incidence and lower FBG levels in high-risk pregnant women.
- Inositol, the DASH diet, and fish oil showed favorable effects on glycemic control, insulin resistance, and gestational weight gain in the included network me-ta-analysis.

**What are the implications of the main findings?**

- Nutritional strategies may have potential value in both the prevention and man-agement of GDM.
- Interventions with high SUCRA rankings but limited direct evidence, such as blueberry plus dietary fiber and magnesium–zinc–calcium–vitamin D supplementation, should be interpreted cautiously and require further validation.

**Abstract:**

Background: While nutritional interventions are pivotal for the prevention and management of gestational diabetes mellitus (GDM), the optimal nutritional strategy remains unclear. Methods: PubMed, EMBASE, Cochrane Library and Web of Science were searched for randomized controlled trials (RCTs). Eligible studies targeted both GDM-diagnosed patients and high-risk cohorts, comparing nutritional interventions with standard controls. Data were extracted and synthesized through network meta-analyses to estimate the standardized mean difference (SMD) or relative risk (RR). The primary outcomes were incidence of GDM, fasting blood glucose (FBG), HOMA-IR, glycated hemoglobin (HbA1c), gestational weight gain (GWG), and cesarean section rate (CSR). Results: A total of 59 trials involving 10,262 women evaluated 24 nutritional strategies. Among high-risk pregnant women, inositol was associated with a reduced incidence of GDM (RR = 0.50, 95% CI: 0.37, 0.68; SUCRA = 84.5%) and lower FBG levels (SMD = −0.42, 95% CI: −0.66, −0.18; SUCRA = 89.8%). For CSR in high-risk pregnant women, blueberry combined with dietary fiber showed a favorable ranking (RR = 0.30, 95% CI: 0.10, 0.90; SUCRA = 97.8%). Among patients with GDM, the Dietary Approaches to Stop Hypertension (DASH) diet showed a significant effect on HOMA-IR (SMD = −2.79, 95% CI: −4.49, −1.09; SUCRA = 95.9%). Plant sterols showed a favorable effect on HbA1c levels (SMD = −1.40, 95% CI: −2.00, −0.80; SUCRA = 95.7%). Fish oil was associated with reduced GWG (SMD = −0.44, 95% CI: −0.76, −0.12; SUCRA = 94.1%). Combined supplementation with magnesium, zinc, calcium, and vitamin D ranked favorably for CSR compared with the control group (RR = 0.44, 95% CI: 0.15, 1.29; SUCRA = 82.1%), but this finding was based on limited evidence. Conclusions: Current evidence suggests that inositol, the DASH diet, plant sterols, and fish oil may play a positive role in the prevention and management of gestational diabetes. However, the effects of interventions with very high SUCRA scores but limited supporting evidence—such as blueberries combined with dietary fiber and magnesium–zinc–calcium–vitamin D supplements—should be interpreted with caution. Well-designed, large-scale randomized controlled trials are needed in the future to further confirm these findings.

## 1. Introduction

Gestational diabetes mellitus (GDM) refers to impaired glucose tolerance first identified during pregnancy but not meeting diagnostic criteria for overt diabetes, representing one of the most common pregnancy-related complications [1]. Previous studies have reported that the global prevalence of GDM has been rising annually, reaching 13.97–14.04%; in China, the prevalence is 14.8% [2]. GDM not only increases maternal risks of polyhydramnios, preterm birth, preterm premature rupture of membranes, cesarean delivery, gestational hypertension, and birth injuries [3], but also elevates the risk of developing type 2 diabetes. Studies have reported that women with a history of GDM have a sevenfold higher risk of developing type 2 diabetes compared to women without such a history [4]. Additionally, common risk factors for GDM include a Body Mass Index (BMI) ≥ 25 kg/m^2^, family history of diabetes, age ≥ 35 years, history of GDM, polycystic ovary syndrome (PCOS), adverse obstetric history, history of macrosomic delivery, and history of fetal abnormalities [5]. Pregnant women with even one of these risk factors are considered high-risk for GDM [6]. Without timely intervention, this population faces a significantly increased risk of developing GDM [7]. Therefore, implementing effective management for GDM patients and high-risk individuals remains a critical clinical priority.

As a non-pharmacological approach, the role of nutritional strategies in diabetes prevention and intervention has garnered widespread attention [8,9]. Nutritional strategies offer economic and easily implementable advantages, effectively regulating blood glucose levels in pregnant women and holding significant value in preventing and managing GDM [10]. Nutritional interventions can regulate the metabolic status of GDM patients in multiple ways. For example, dietary fiber supplements can improve blood glucose levels and insulin resistance [11], low-sugar diets improve glycated hemoglobin levels [12], and consuming whole grains, fruits, and vegetables can regulate blood glucose and insulin metabolism [13]. Additionally, vitamin D supplementation enhances insulin sensitivity, promotes insulin secretion, and protects β-cells, significantly reducing diabetes risk [14]. For women with GDM, vitamin D supplements markedly improve glycemic control and increase insulin sensitivity [15]. However, the optimal nutritional intervention strategy for preventing and managing GDM remains uncertain.

As a commonly used method in evidence-based research, meta-analysis is frequently employed. Although a traditional meta-analysis found that nutritional supplements such as inositol and vitamins could serve as potential intervention strategies for preventing GDM [16], relying solely on traditional meta-analyses to evaluate the differential effects of multiple nutritional strategies for interventions targeting individuals at high risk for GDM or those with GDM may have certain limitations. Network meta-analysis (NMA) possesses the capability to construct an evidence network, enabling simultaneous comparisons of multiple interventions and comprehensive ranking based on both direct and indirect evidence. This approach offers methodological advantages in identifying optimal interventions [17]. In recent years, Tang et al. employed an NMA to evaluate the effects of physical activity, dietary intervention, probiotic intervention, combined interventions, and inositol supplementation on the incidence of GDM in pregnant women [18]. Zhang et al. conducted a network meta-analysis to evaluate the effects of lifestyle interventions such as dietary modifications and exercise on GDM and infant outcomes [19]. Similarly, Yu et al. performed an NMA to assess the efficacy of different nutrients in managing blood glucose levels, inflammation, and oxidative stress in GDM patients [20]. Collectively, these prior NMAs each addressed either GDM prevention or GDM management in isolation, and evaluated a comparatively narrow range of nutritional strategies and outcomes. To date, few NMAs have a comprehensive spectrum of nutritional strategies—including vitamins, minerals, dietary supplements, probiotics, and structured dietary programs—into a single evidence network covering both high-risk prevention and GDM management. Furthermore, no prior study has jointly ranked these interventions across six core glycemic, weight-related, and delivery outcomes. Against this backdrop, this study aims to explore the efficacy of various nutritional strategies for preventing or managing GDM in high-risk populations or GDM patients through systematic review and network meta-analysis. It seeks to comprehensively compare the effects of multiple nutritional intervention approaches and identify the optimal intervention strategy based on current evidence.

## 2. Materials and Methods

We conducted the systematic review and network meta-analysis according to the PRISMA-NMA guidelines (Table S1). The study protocol was registered with PROSPERO (CRD420251167123).

### 2.1. Data Sources and Search Strategy

We employed a combination of controlled vocabulary and free-text search terms, including “gestational diabetes”, “nutritional support”, “diet therapy”, “vitamins”, “probiotics”, “fish oils”, and “myoinositols” to conduct a comprehensive search across four databases: PubMed, Cochrane, Web of Science, and EMBASE. The search period spanned from the inception of each database to 31 July 2025. The search was not restricted by language. Reference lists of relevant studies were reviewed to identify additional research. Detailed search strategies are summarized in the search strategy table (Table S2).

### 2.2. Inclusion and Exclusion Criteria

The following criteria were used to include the articles in this review: (1) The study subjects were high-risk populations of GDM and patients with GDM. Pregnant women with ≥1 risk factor for GDM were considered high-risk. These risk factors include BMI ≥ 25 kg/m^2^, family history of diabetes, age ≥ 35 years, history of GDM, PCOS, adverse obstetric history, history of macrosomic delivery, and history of fetal abnormalities [6]. GDM was diagnosed using the IADPSG criteria [3]. (2) Nutritional intervention was implemented in the trials, including various dietary regimens and nutritional supplements. (3) A placebo or regular diet was used in the control group. (4) Outcomes included incidence of GDM, fasting blood glucose (FBG) (Reduced FBG may suggest better short-term glycemic control), HOMA-IR index (Reduced HOMA-IR suggests better insulin sensitivity), glycated hemoglobin (HbA1c) (Reduced HbA1c suggests better long-term glycemic control), gestational weight gain (GWG) (Weight management during pregnancy is closely related to glycemic control), or cesarean section rate (CSR). (5) Studies were randomized controlled trials (RCTs) and published in English. The exclusion criteria were as follows: (1) nonrandomized controlled studies and animal studies; (2) studies lacking critical data (intervention measures, number of subjects, primary outcomes); (3) non-English publications; and (4) papers published as abstracts only.

After retrieving and removing duplicate records, the literature was preliminarily screened based on inclusion criteria through title and abstract review. Subsequently, full-text screening was conducted on the included studies to finalize the selection for analysis. The entire literature screening process was performed independently by two researchers (Shi and Chen), with discrepancies resolved through group discussion (Shi, Chen, Tang, and Ren).

### 2.3. Data Extraction

Data extraction and assessment were performed independently by three authors (Shi, Chen and Tang). Discrepancies were resolved through group discussion. A standardized Excel template was used to extract the following information: authors, year, country, study population (sample size, age), intervention (nutritional intervention protocol, dosage), and outcome measures (GDM incidence, FBG, HOMA-IR, HbA1c, GWG, CSR). In cases of overlapping data, the study with the most comprehensive dataset was selected. Discrepancies were resolved through group discussion.

### 2.4. Quality Assessment

The Cochrane Risk of Bias Tool for Randomized Trials (ROB 2.0) was used to assess the risk of bias [21]. According to the tool, the overall risk of bias for each trial is classified as ‘low’ if all domains are ‘low risk’, and ‘high’ if any domain is ‘high risk’. If no domains were classified as high risk, but one or more raised some concerns, the overall risk of bias for that trial was categorized as ‘some concerns’. Study quality was assessed independently by two researchers (Shi and Chen), with disagreements resolved through group discussion.

### 2.5. Assessment of Evidence Certainty

The certainty of evidence for the results of the primary network meta-analysis was assessed using the GRADE approach. For each primary comparison and outcome, the certainty of evidence was rated as high, moderate, low, or very low.

### 2.6. Data Analysis

Data analysis was performed using Stata 15.0 software, mainly with the network package for network meta-analysis. The risk ratio (RR) and its 95% confidence interval (CI) were used for dichotomous variables, and the standardized mean difference (SMD) and its 95% CI were used for continuous variables. Network meta-analysis was conducted under the assumptions of similarity, transitivity, and consistency. To reduce clinical heterogeneity, high-risk pregnant women and patients with diagnosed GDM were analyzed separately, because these populations differ in metabolic status and intervention goals. The transitivity assumption was assessed by considering key study characteristics, including population type, baseline BMI, gestational age at intervention initiation, intervention duration, and outcome definitions.

Although the included interventions were diverse, including dietary patterns, vitamins, minerals, probiotics, dietary supplements, and combined nutritional interventions, they were all non-pharmacological nutritional strategies evaluated in randomized controlled trials for GDM prevention or management. Each nutritional strategy was therefore treated as a separate node in the network. Direct comparative relationships were visualized using network plots. Node size represented the sample size of each intervention, while line thickness reflected the number of direct comparisons.

The node-splitting method was used to evaluate local inconsistency when closed loops were available, while the Deviance Information Criterion was used to assess global inconsistency. A consistency model was applied when no significant inconsistency was detected, and an inconsistency model was used when significant inconsistency was observed (*p* < 0.05). Heterogeneity was assessed using the I^2^ statistic. SUCRA values were calculated to rank interventions but were interpreted together with effect sizes, 95% CIs, number of studies, risk of bias, and certainty of evidence. League tables were constructed to summarize direct and indirect comparisons. Sensitivity analyses were performed by excluding studies judged to have a high overall risk of bias according to the ROB 2.0 assessment. These analyses were conducted to examine whether studies with high risk of bias substantially influenced the pooled effect estimates and SUCRA-based rankings. Publication bias was assessed using comparison-adjusted funnel plots.

## 3. Results

### 3.1. Study Selection

**Figure 1:**
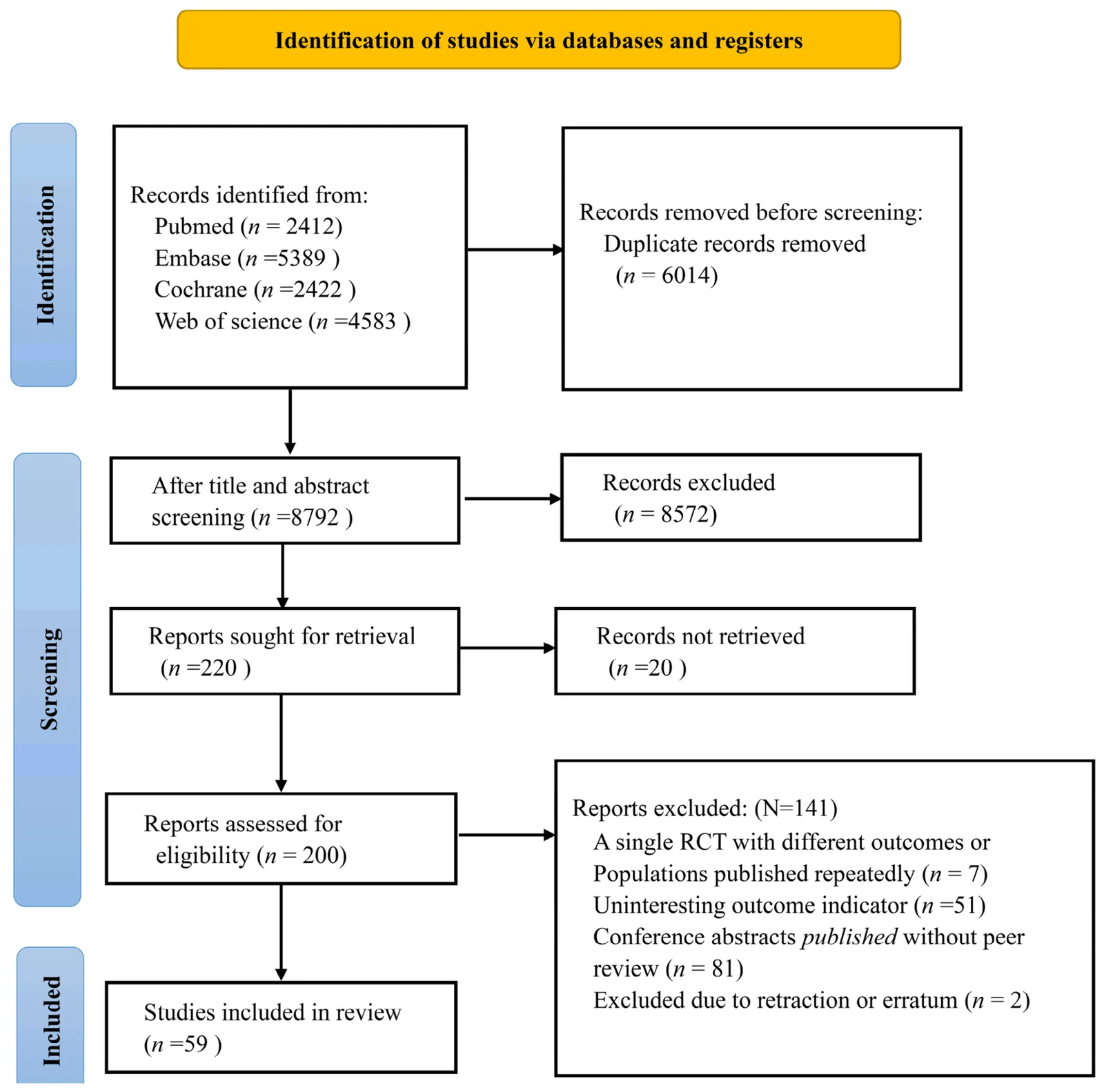
Inclusion in the literature screening flowchart.

A total of 14,806 records were retrieved from the database. After removing 6014 duplicate records, 8792 records underwent preliminary screening based on titles and abstracts. After excluding 8572 records, full texts were retrieved for 220 records. Of these, 20 records were excluded due to unavailability of full text, leaving 200 records for full-text review. Following application of inclusion and exclusion criteria, 141 articles were excluded for non-compliance, resulting in the final inclusion of 59 RCT studies [11,12,13,14,15,22,23,24,25,26,27,28,29,30,31,32,33,34,35,36,37,38,39,40,41,42,43,44,45,46,47,48,49,50,51,52,53,54,55,56,57,58,59,60,61,62,63,64,65,66,67,68,69,70,71,72,73,74,75]. The detailed screening process is shown in Figure 1.

### 3.2. Characteristics of Included Studies

Table 1 and Table 2 describe the overall characteristics of the included studies, which were published between 2011 and 2025 and involved a total of 10,262 participants. Seventeen countries were represented: Iran (*n* = 17), China (*n* = 11), United States (*n* = 5), Italy (*n* = 6), Australia (*n* = 4), Turkey (*n* = 2), Finland (*n* = 2), Israel (*n* = 2), United Kingdom (*n* = 2), Canada (*n* = 1), Denmark (*n* = 1), Greece (*n* = 1), Mexico (*n* = 1), New Zealand (*n* = 1), Pakistan (*n* = 1), Spain (*n* = 1), and Thailand (*n* = 1). These studies employed a total of 24 nutritional strategies: probiotics (Pb, *n* = 11), inositol (Ins, *n* = 11), low glycemic index diet (LGD, *n* = 6), vitamin D (VD, *n* = 7), fish oil (FO, *n* = 4), Dietary Approaches to Stop Hypertension (DASH, *n* = 3), high-carbohydrate low-fat diet (CHOICE, *n* = 3), dietary fiber (DF; *n* = 2), plant sterols (PS, *n* = 1), omega-3 (*n* = 1), macronutrient preloading (MNP, *n* = 1), reduced energy diet (RED, *n* = 1), moderate carbohydrate high-fiber diet (MCRD, *n* = 1), modestly lower carbohydrate diet (MLC, *n* = 1). probiotics + selenium (Pb + Se, *n* = 1), inositol + α-lactalbumin (Ins + La, *n* = 1), blueberry + dietary fiber (BB + DF, *n* = 1), fish oil + probiotics (FO + Pb, *n* = 1), magnesium–zinc–calcium–vitamin D complex (MgZnCaVD, *n* = 1), magnesium + vitamin E (Mg + VE, *n* = 1), omega-3 + vitamin D (Omega-3 + VD, *n* = 1), omega-3 + vitamin E (Omega-3 + VE, *n* = 1), vitamin D + evening primrose oil (VD + EPO, *n* = 1), and low glycemic index diet + dietary fiber (LGD + DF, *n* = 1).

### 3.3. Risk of Bias of the Included Studies and Sensitivity Analyses

According to the RoB2 assessment, among 59 RCTs, 35 had low risk of bias, 21 had some concerns and 3 had high risk of bias. For the “Randomization process” domain, all studies were rated as low risk.

**Figure 2:**
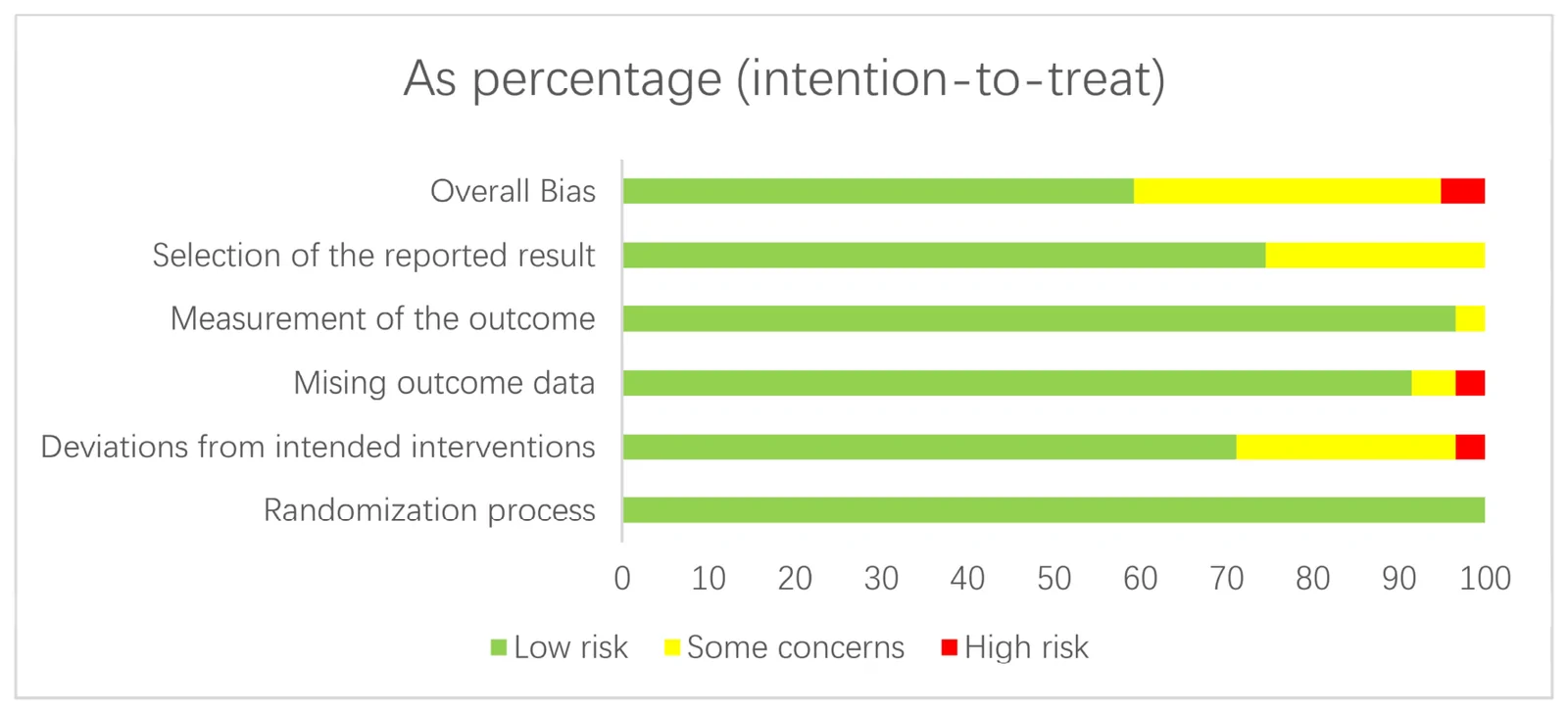
Risk of bias assessment for included studies.

The primary source of high risk of bias in Basu et al.’s study was deviations from the intended interventions [28]. In Dilli et al.’s study [37], the primary source was both deviations from the intended interventions and missing outcome data. In Li et al.’s study [57], the primary source was missing outcome data. The quality assessment results are shown in Figure 2, with detailed findings available in Table S3.

**Figure 3:**
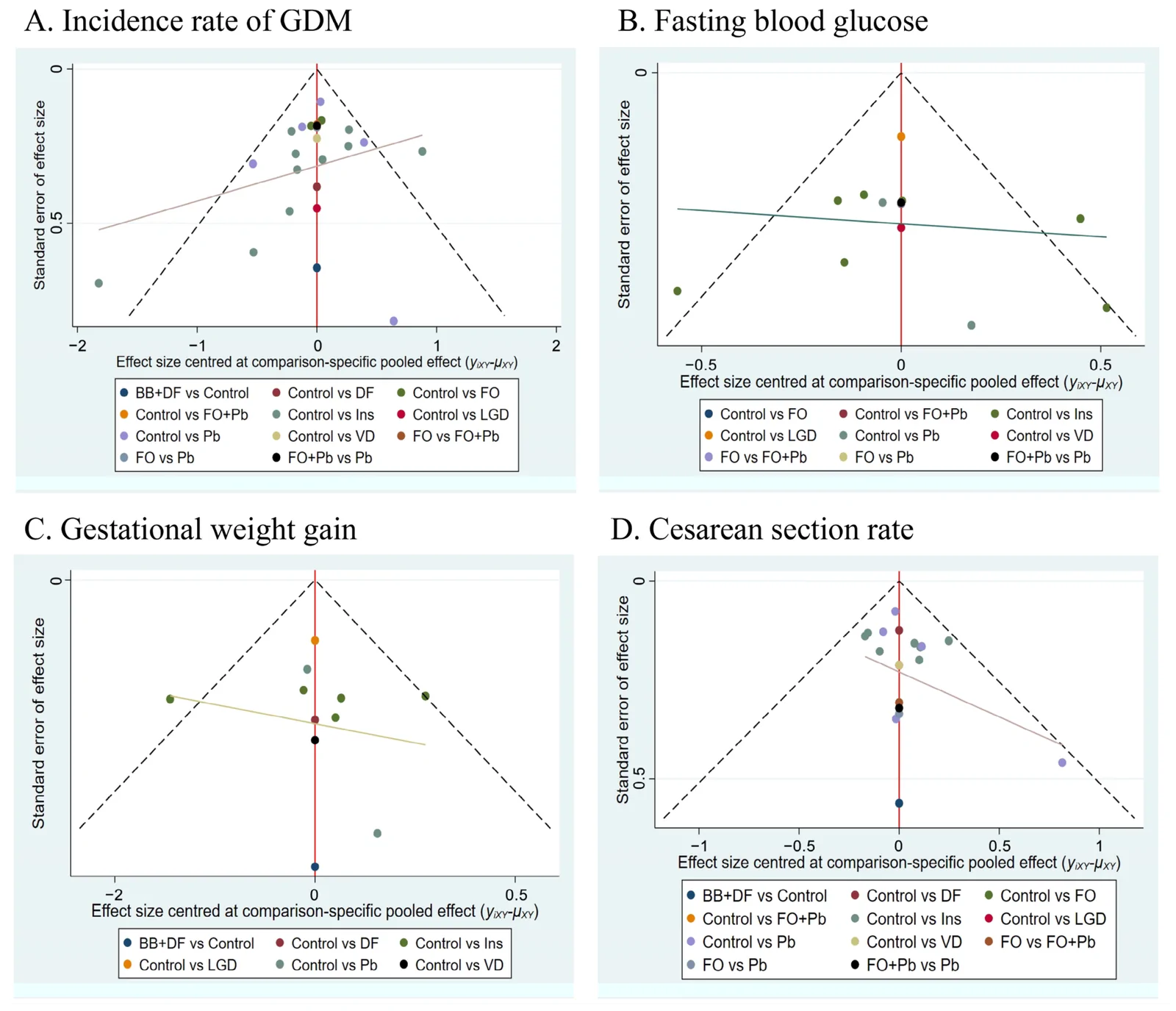
Funnel plots for high-risk populations of gestational diabetes mellitus. Each point represents one study comparison; the vertical red line indicates the null effect, the diagonal lines represent the expected 95% CI, and the oblique fitted line indicates the trend of funnel plot asymmetry.

**Figure 4:**
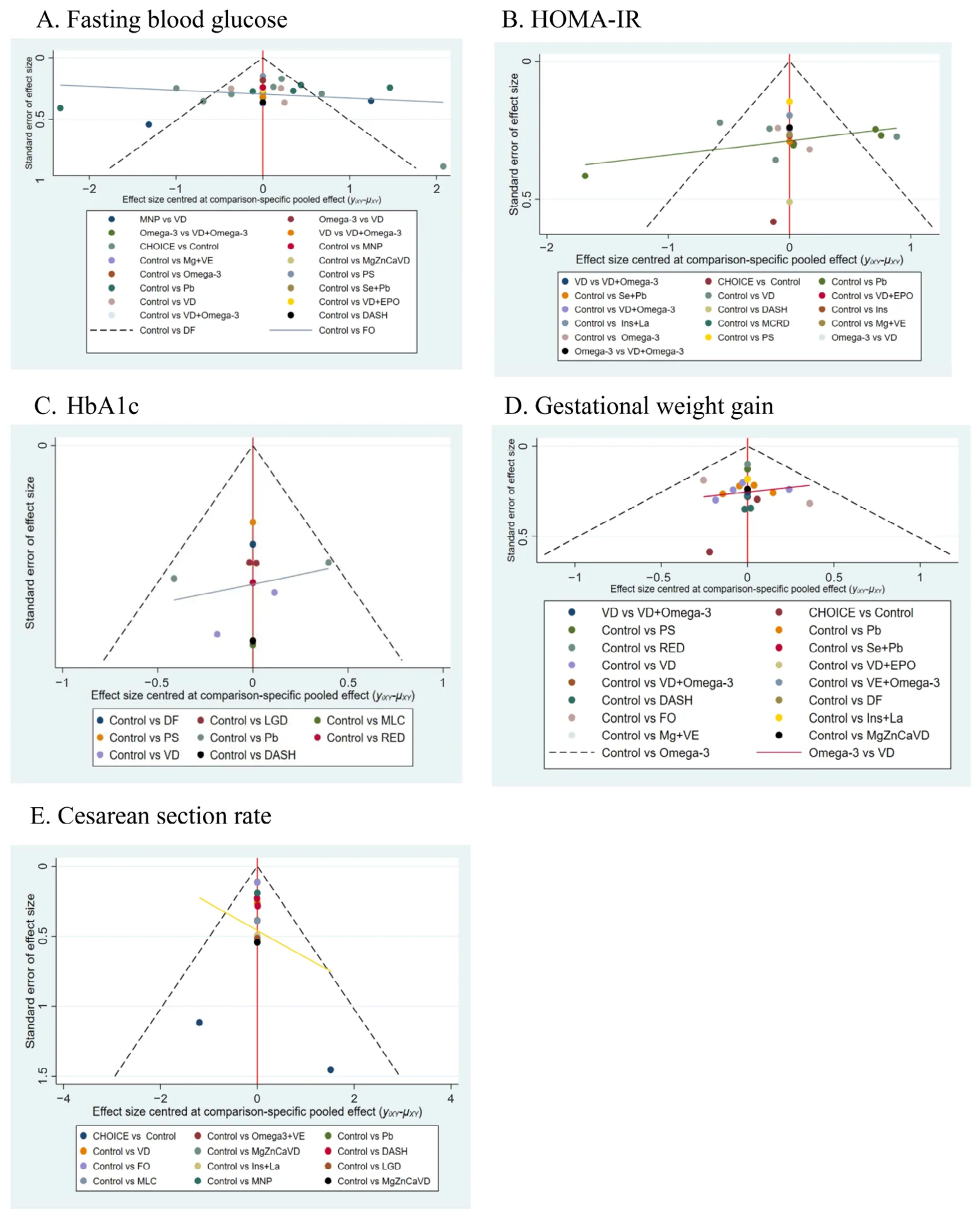
Funnel plots for patients with gestational diabetes mellitus. Each point represents one study comparison; the vertical red line indicates the null effect, the diagonal lines represent the expected 95% CI, and the oblique fitted line indicates the trend of funnel plot asymmetry.

We also performed publication bias analysis using Stata. The funnel plots and Egger’s tests for most outcome measures (Figure 3, Figure 4, Figures S3 and S4) showed scatter plots with near-symmetrical inverted funnel shapes. Egger’s tests yielded *p* > 0.05, indicating a low likelihood of publication bias. However, the funnel plot and Egger’s linear regression test (Egger’s test, t = −2.44; *p* = 0.023, 95% CI: −3.36, −0.28) for the outcome of GDM incidence in high-risk populations indicated the presence of publication bias. The funnel plot suggests that probiotics and fish oil may exhibit relatively large bias.

To evaluate the robustness of the findings, sensitivity analyses were performed after excluding studies rated as having a high risk of bias. Overall, the main effect estimates and SUCRA-based rankings were generally consistent with those of the primary analysis, suggesting that the main findings were not substantially driven by high-risk studies. Results of the sensitivity analysis after excluding high-risk studies are provided in Figure S5.

### 3.4. Certainty of Evidence

The certainty of evidence was assessed using the GRADE approach (Table S6). The overall certainty of evidence for the primary outcome networks was generally rated as moderate. However, this rating refers to the overall network-level evidence for each outcome and should not be interpreted as moderate certainty for every individual intervention comparison. For comparisons supported by only one small trial, such as blueberry combined with dietary fiber, plant sterols, and combined magnesium–zinc–calcium–vitamin D supplementation, the certainty of evidence remains limited and should be interpreted cautiously. The evidence was mainly downgraded because of some concerns regarding risk of bias.

### 3.5. Outcomes in High-Risk Populations for GDM

#### 3.5.1. Incidence of GDM

**Figure 5:**
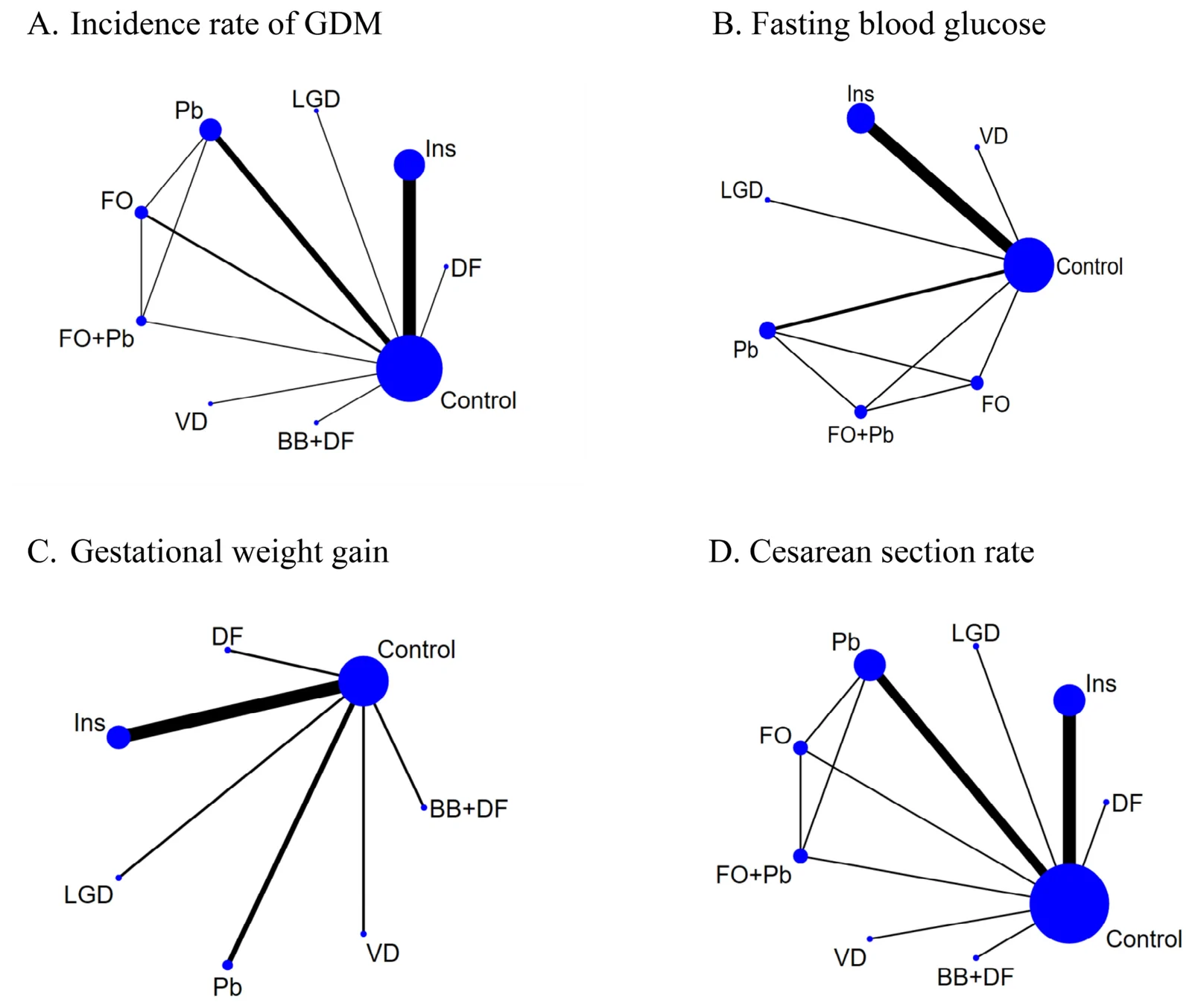
Network plots for high-risk populations of gestational diabetes mellitus. Nodes represent interventions; node size and edge thickness are proportional to sample size and study count, respectively. Abbreviations are defined in the Abbreviations Section.

**Figure 6:**
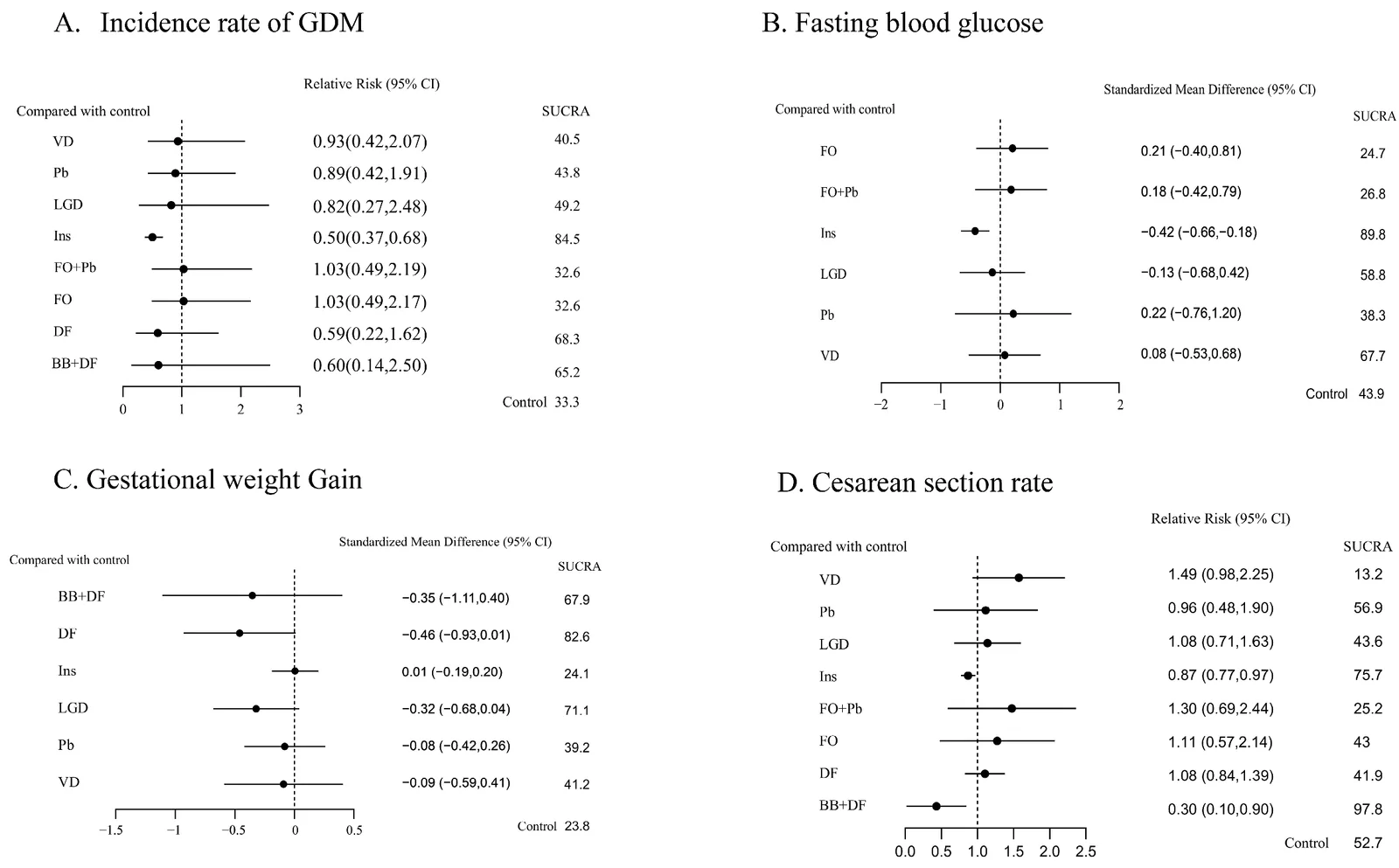
Forest plot comparison with the control group and SUCRA in high-risk populations of gestational diabetes mellitus. Black circles with horizontal lines represent effect estimates with their corresponding 95% confidence intervals.

A total of 20 studies reported the incidence of GDM, covering 6218 individuals at high risk for GDM. The network plot is shown in Figure 5A. Inositol was compared most frequently, involving 10 studies. The global inconsistency test indicated no significant difference in efficacy between the consistency model and the inconsistency model (*p* = 0.93). Local inconsistency testing via node splitting revealed no significant difference in efficacy between direct and indirect comparisons (*p* > 0.05). League Tables for Sensitive Analysis showed that inositol (RR = 0.50, 95% CI: 0.37, 0.68; SUCRA = 84.5%) demonstrated significant efficacy in reducing GDM risk (Figure 6A and Figure S2, Table S4).

#### 3.5.2. Fasting Blood Glucose

A total of 11 studies investigated the effects of nutritional interventions on FBG levels in 2270 individuals at high risk of GDM. The network plot is shown in Figure 5B. Inositol was compared most frequently, involving seven studies. The global inconsistency test revealed no significant difference in efficacy between the consistency model and the inconsistency model (*p* = 0.66). Local inconsistency testing via node splitting revealed no significant difference in efficacy between direct and indirect comparisons (*p* > 0.05). Analysis using the consistency model showed that inositol (SMD = −0.42, 95% CI: −0.66, −0.18; SUCRA = 89.8%) significantly reduced FBG levels in high-risk pregnant women compared to the control group (Figure 6B and Figure S2, Table S4).

#### 3.5.3. Gestational Weight Gain

Eleven studies reported GWG in high-risk GDM populations, encompassing 2404 pregnant women. The network plot is shown in Figure 5C. Inositol was compared most frequently, involving five studies. The global inconsistency test revealed no difference in efficacy between the consistency model and the inconsistency model (*p* = 0.72). Local inconsistency testing via node splitting did not identify significant local inconsistencies (*p* > 0.05). Analysis using the consistency model showed no significant difference in pairwise comparisons between all interventions (Figure 6C and Figure S2, Table S4).

#### 3.5.4. Cesarean Section Rate

Sixteen studies reported CSR among high-risk pregnancy populations, involving 3443 women at high risk for GDM. The network plot is shown in Figure 5D. Inositol interventions were the most frequently studied, with seven studies included. Global inconsistency testing revealed no significant difference in efficacy between the consistency and inconsistency models (*p* = 0.96). Local inconsistency testing via node splitting did not identify significant local inconsistencies (*p* > 0.05). Analysis using the consistency model revealed that inositol demonstrated superior efficacy in reducing CSR compared to vitamin D (RR = 0.58, 95% CI: 0.38–0.90) and the control group (RR = 0.87, 95% CI: 0.77, 0.97) (Figure S2 and Figure 6D). Furthermore, the combined intervention of blueberries and dietary fiber significantly outperformed low glycemic index diets (RR = 0.28, 95% CI: 0.09, 0.91), fish oil combined with probiotics (RR = 0.23, 95% CI: 0.07, 0.82), fish oil (RR = 0.27, 95% CI: 0.08, 0.98), dietary fiber (RR = 0.28, 95% CI: 0.09, 0.86), and the control group (RR = 0.30, 95% CI: 0.10, 0.90) (Figure S2). A SUCRA analysis revealed that, among high-risk pregnancies, the group receiving a combined blueberry and dietary fiber intervention demonstrated the greatest reduction in CSR (97.8%), followed by the inositol intervention group (75.6%) (Figure 6D and Figure S1, Table S4). However, this finding should be interpreted with caution, as the blueberry-plus-dietary-fiber intervention is supported by only one trial. Therefore, its high SUCRA ranking may be unstable and should not be regarded as conclusive evidence of superiority.

### 3.6. Outcomes in GDM Patients

#### 3.6.1. Fasting Blood Glucose

**Figure 7:**
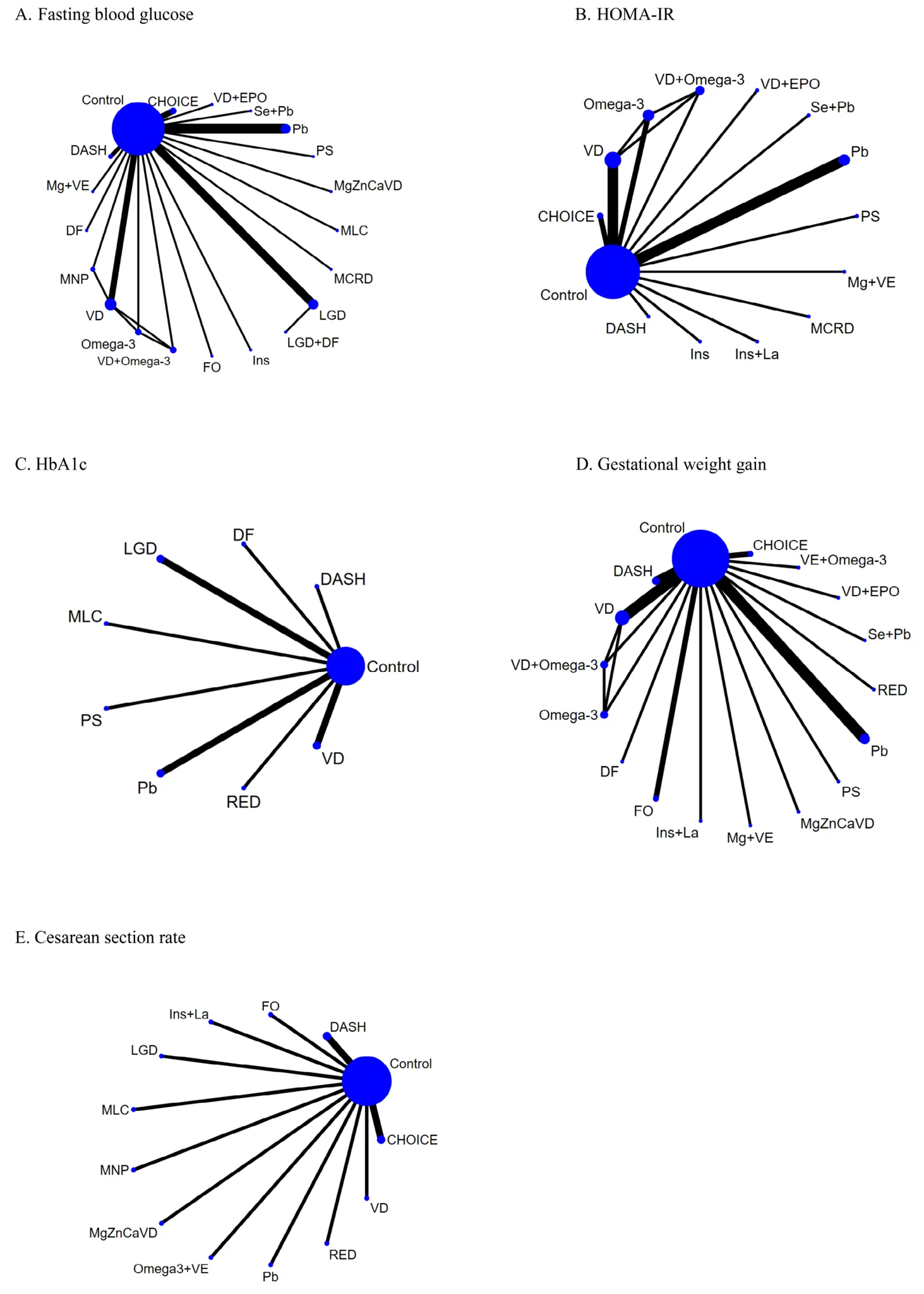
Network plots for patients with gestational diabetes mellitus. Nodes represent interventions; node size and edge thickness are proportional to sample size and study count, respectively. Abbreviations are defined in the Abbreviations Section.

**Figure 8:**
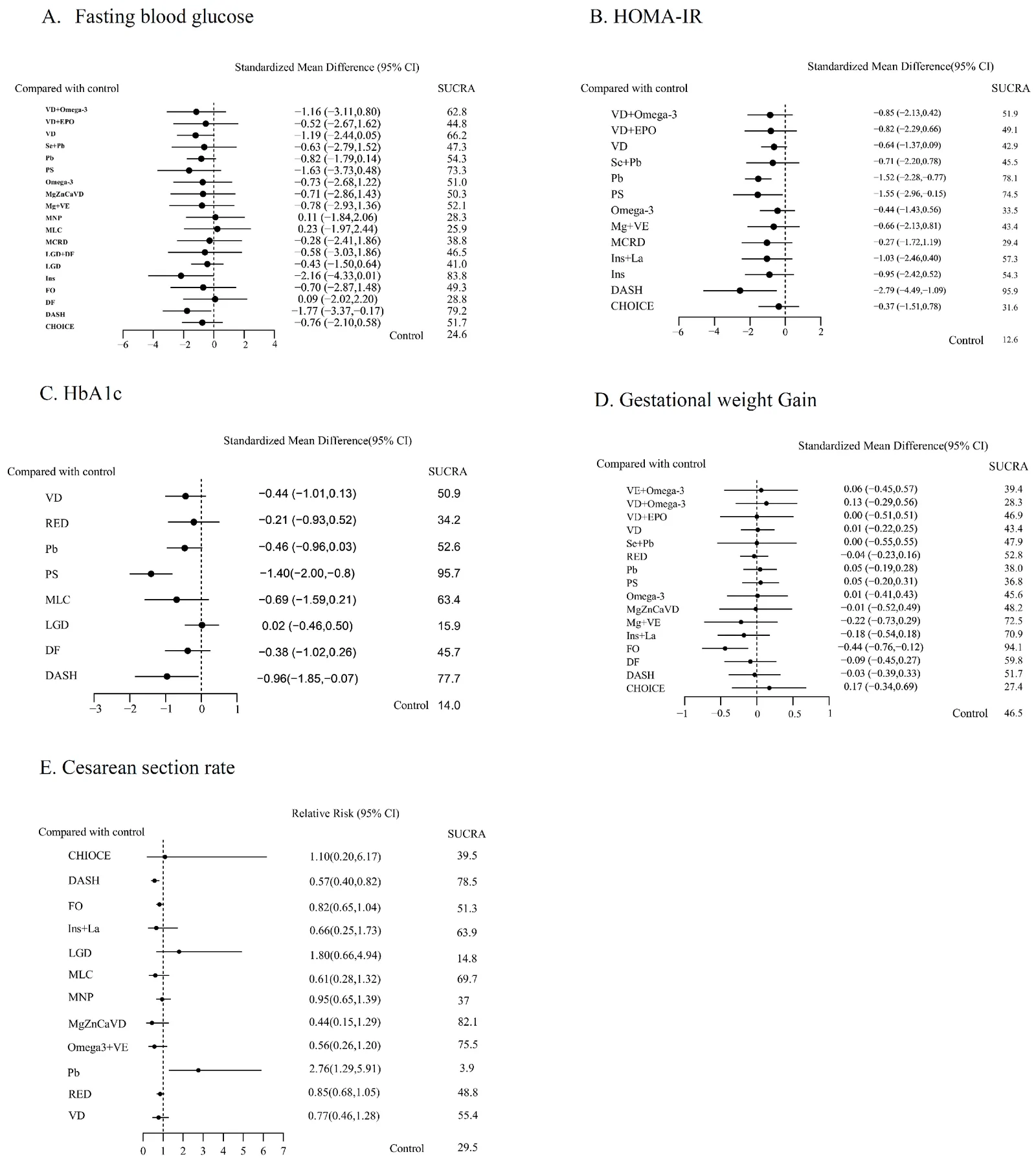
Forest plot comparison with the control group and SUCRA in patients with gestational diabetes mellitus. Black circles with horizontal lines represent effect estimates with their corresponding 95% confidence intervals.

A total of 29 studies reported FBG levels in GDM, involving 2001 pregnant women with GDM. The network plot is shown in Figure 7A. Probiotics were compared most frequently, involving 5 studies. The global inconsistency test showed no significant difference in efficacy between the consistency model and the inconsistency model (*p* = 0.92). Local inconsistency testing via node splitting revealed no significant local inconsistencies (*p* > 0.05). In the overall analysis without subgroup stratification, the DASH diet showed the highest SUCRA ranking for reducing FBG among patients with GDM and demonstrated a significant reduction compared with the control group (SMD = −1.77, 95% CI: −3.37, −0.17; SUCRA = 79.2%). In the subgroup analysis stratified by country income level, inositol ranked highest among studies conducted in high-income countries and showed a significant reduction in FBG compared with the control group (SMD = −2.16, 95% CI: −3.81, −0.52). In contrast, among studies conducted in low- and middle-income countries, the DASH diet had the highest SUCRA ranking; however, the difference compared with the control group did not reach statistical significance (SMD = −1.80, 95% CI: −3.77, 0.16). These subgroup findings suggest that the relative ranking of nutritional interventions for FBG may vary according to country income level, although the results should be interpreted cautiously because of the limited number of studies within each subgroup. (Figure 8A, Figures S1, S2 and S4, Table S4).

#### 3.6.2. HOMA-IR

Nineteen studies reported the HOMA-IR index of 1374 GDM patients, with the network plot shown in Figure 7B. Probiotics were the most frequently compared intervention, involving 4 studies. The global inconsistency test revealed no significant difference in efficacy between the consistency model and the inconsistency model (*p* = 0.96). Local consistency testing via node splitting revealed no significant local inconsistencies (*p* > 0.05). Analysis using the consistency model showed that compared to the control group, probiotics (SMD = −1.52, 95% CI: −2.28, −0.77), plant sterols (SMD = −1.55, 95% CI: −2.96, −0.15), and the DASH diet (SMD = −2.79, 95% CI: −4.49, −1.09) significantly reduced the HOMA-IR index compared to the control group (Figure 8B and Figure S2). Specifically, the DASH intervention demonstrated superior efficacy compared to Vitamin D (SMD= −2.15, 95% CI: −4.00, −0.30), Omega-3 (SMD = −2.36, 95% CI: −4.33, −0.39), MCRD (SMD = −2.53, 95% CI: −4.76, −0.29) and CHOICE (SMD = −2.43, 95% CI: −4.47, −0.38) (Figure S2). SUCRA analysis showed (Figure 8B and Figure S1, Table S4) that DASH had the best effect in reducing insulin resistance (95.9%), followed by probiotics (78.1%) and plant sterols (74.5%).

#### 3.6.3. Glycated Hemoglobin

Eleven studies reported the effect of nutritional interventions on HbA1c in GDM population, as shown in Figure 7C. These studies covered 903 pregnant women with GDM. Probiotics, Vitamin D and LGD were compared the most times, involving 2 studies. The global inconsistency test revealed no significant difference in efficacy between the consistency model and the inconsistency model (*p* = 0.13). Since the network plot did not form closed loops, node splitting was not applied. All analyses were conducted within the consistency model framework. League Tables showed that PS were superior to vitamin D (SMD = −0.96, 95% CI: −1.79, −0.14), RED (SMD = −1.19, 95% CI: −2.14, −0.25), probiotics (SMD = −0.94, 95% CI: −1.71, −0.16), LGD (SMD = −1.42, 95% CI: −2.19, −0.65), DF (SMD = −1.02, 95% CI: −1.90, −0.15), and the control group (SMD = −1.40, 95% CI: −2.00, −0.80) (Figure S2). The DASH intervention showed significantly greater effects than the control group (SMD = −0.96, 95% CI: −1.85, −0.07) (Figure 8C). SUCRA analysis indicated that PS (95.7%) demonstrated the strongest effect on reducing HbA1c, followed by DASH (77.7%) (Figure 8C and Figure S1 and Tables S4 and S5). However, this finding should be interpreted with caution, as the PS intervention is supported by only one trial. Therefore, its high SUCRA ranking may be unstable and should not be regarded as conclusive evidence of superiority.

#### 3.6.4. Gestational Weight Gain

Twenty-four studies reported on GWG in 2198 individuals with GDM. The network plot is shown in Figure 7D. Probiotics were compared the most, with four studies being involved. Global inconsistency testing revealed no significant difference in efficacy between the consistency model and inconsistency model (*p* = 0.25). Local inconsistency testing via node splitting did not identify significant local inconsistencies (*p* > 0.05). Analysis using the consistency model revealed (Figure S2) that FO intervention was more effective than vitamin D combined with omega-3 (SMD = −0.57, 95% CI: −1.10, −0.04), vitamin D alone (SMD = −0.45, 95% CI: −0.85, −0.06), RED (SMD = −0.40, 95% CI: −0.78, −0.03), probiotics (SMD = −0.49, 95% CI: −0.88, −0.09), Pb (SMD = −0.49, 95% CI: −0.88, −0.09), PS (SMD = −0.49, 95% CI: −0.90, −0.09), control group (SMD = −0.44, 95% CI: −0.76, −0.12), and CHOICE (SMD = −0.61, 95% CI: −1.22, −0.00). SUCRA analysis indicated that FO interventions showed the strongest effect on controlling GWG (94.1%) (Figure 8D and Figure S1 and Tables S4 and S5).

#### 3.6.5. Cesarean Section Rate

Fourteen studies reported CSR among 1255 women with GDM; the network plot is shown in Figure 7E. CHOICE and DASH were compared most frequently, each included in two studies. A consistency model was applied because the absence of closed loops precluded the assessment of global or local inconsistency. Accordingly, results derived from this model indicated that vitamin D alone (RR = 0.28, 95% CI: 0.11, 0.70), RED (RR = 0.31, 95% CI: 0.14, 0.68), omega-3 combined with vitamin E (RR = 0.20, 95% CI: 0.07, 0.59), MgZnCaVD complex supplements (RR = 0.16, 95% CI: 0.04, 0.60), MNP (RR = 0.35, 95% CI: 0.15, 0.81), MLC (RR = 0.22, 95% CI: 0.08, 0.66), inositol plus α-Lactalbumin (RR = 0.24, 95% CI: 0.07, 0.81), FO (RR = 0.30, 95% CI: 0.13, 0.66), DASH diet (RR = 0.21, 95% CI: 0.09, 0.48) and control group (RR = 0.36, 95% CI: 0.17, 0.78) were more effective than probiotics in reducing the CSR. Additionally, the DASH diet showed superior effects compared to the control group (RR = 0.57, 95% CI: 0.40, 0.82), and the LGD diet (RR = 0.32, 95% CI: 0.11, 0.93) (Figure S2). SUCRA analysis indicated that the MgZnCaVD complex (82.1%) had the strongest intervention effect (Figure 8E and Figure S1, Table S4). Similarly, although magnesium, zinc, calcium, and vitamin D supplements ranked highest in reducing the cesarean section rate among women with gestational diabetes, this finding is based on limited evidence and should be considered a preliminary conclusion.

## 4. Discussion

To our knowledge, this is one of the few systematic reviews and network meta-analyses to evaluate multiple nutritional strategies across both high-risk pregnant women and women diagnosed with GDM. The findings suggest that inositol may have beneficial effects on GDM incidence and FBG in high-risk populations. Blueberry combined with dietary fiber showed a favorable ranking for reducing CSR, but this result should be interpreted cautiously because it was supported by limited evidence. Among women with GDM, the DASH diet, plant sterols, fish oil, and combined magnesium–zinc–calcium–vitamin D supplementation showed potential benefits for selected outcomes, including FBG, HOMA-IR, HbA1c, GWG, and CSR.

Our research indicates that inositol is the most effective nutritional approach for reducing the incidence of GDM, which is consistent with the findings of the meta-analysis conducted by Wei [76]. Evidence suggests that taking 4 g of myo-inositol (MI) every day may reduce the likelihood and severity of GDM. Research by Dell’Edera et al. also found that inositol and its derivatives can reduce the incidence of GDM, with 250 mg daily of D-chiro-inositol (DCI) and 200 mg daily of MI potentially representing the optimal combination dosage. Consequently, such regimens may serve as a viable alternative strategy when oral hypoglycemic agents are contraindicated or unavailable [77]. Its potential mechanism of action involves inositol upregulating key proteins in the insulin signaling pathway—IRS2, PI3K, AKT, and their phosphorylation levels, thereby promoting the expression of the glucose transporter GLUT4 and inhibiting glycogen synthase kinase GSK3β. This synergistically enhances the capacity for glucose uptake and glycogen synthesis in the liver and cells, ultimately significantly improving blood glucose homeostasis and reducing the accumulation of advanced glycation end products. It can also be considered that inositol prevents GDM by enhancing insulin resistance [78].

Our findings also suggest that inositol may have beneficial effects on improving FBG levels in individuals at high risk of GDM and in patients with GDM. These results are consistent with those of several previous studies [79,80]. Pintaudi et al. also found that twice-daily inositol treatment over 3 months significantly reduced fasting blood glucose and glycated hemoglobin in patients with type 2 diabetes [81]. The mechanism may involve inositol enhancing insulin-mediated glucose uptake, improving peripheral tissue glucose utilization, and alleviating insulin resistance associated with β-cell dysfunction, thereby increasing insulin sensitivity and regulating blood glucose levels [82]. The subgroup analysis further suggested that country income level may modify the relative ranking of nutritional interventions for FBG among patients with GDM. Inositol ranked highest in studies from high-income countries, whereas DASH ranked highest in studies from low- and middle-income countries. However, because the number of studies within each subgroup was limited and the confidence interval for DASH in low- and middle-income countries crossed the null value, these findings should be interpreted as exploratory and require confirmation in future studies.

The DASH diet showed a comparatively advantageous effect on reducing HOMA-IR in patients with GDM. These findings are consistent with the results of the network meta-analysis by Di [83]. Di et al.’s network meta-analysis included 28 studies evaluating the effects of seven dietary patterns on glycemic control and pregnancy outcomes in women with GDM. They found that the DASH diet was the most effective intervention and significantly reduced insulin resistance [83]. Kechagia and other studies have also demonstrated that the DASH diet not only lowers fasting serum insulin levels but also exerts positive effects on insulin resistance [84,85,86].

Plant sterols ranked highest for reducing glycated hemoglobin levels in patients with GDM, consistent with the meta-analysis by Salehi-Sahlabadi et al. [87]. The study found that hyperglycemic patients with daily intake of 1–2 g of phytosterols exhibited lower HbA1c levels. The mechanism by which phytosterols influence glycated hemoglobin may be related to the expression and translocation of GLUT4 in skeletal muscle, liver, and white adipose tissue [87]. However, this finding should be interpreted cautiously because of the limited supporting evidence.

Fish oil is the optimal nutritional strategy for controlling weight gain in GDM patients. The n-3 PUFAs abundant in fish oil effectively slow weight increase, and whether added to the diet or taken as supplements, they may play a positive role in maintaining weight loss [88]. Previous studies have shown that n-3 PUFAs can effectively mitigate weight gain. This is achieved by improving body composition and counteracting obesity-related metabolic changes. Mechanisms involved include regulating lipid metabolism, modulating adipokines (e.g., adiponectin and leptin), reducing adipose tissue inflammation and altering epigenetic mechanisms [89]. An animal study further confirmed that n-3 PUFAs can improve hepatic insulin resistance and effectively reduce weight gain in rats [90].

In this network meta-analysis, the combination of blueberries and dietary fiber showed a favorable SUCRA ranking in reducing the cesarean section rate among high-risk populations. However, this finding should be interpreted with caution, as only one study supported this conclusion, making these results preliminary. Although previous studies in non-pregnant populations have suggested that blueberries and dietary fiber may improve insulin sensitivity and glycemic control, these mechanisms have not been directly validated in pregnant women. Furthermore, the cesarean section rate (CSR) is influenced by a variety of obstetric and clinical factors and is not solely related to glycemic control [91,92,93,94]. Therefore, the potential mechanism of action of the combined use of blueberries and dietary fiber on the CSR remains uncertain and requires further validation in future large-scale randomized controlled trials.

Our NMA showed that combined supplementation with magnesium, zinc, calcium, and vitamin D had a favorable SUCRA ranking in reducing the CSR among women with GDM. Similarly, due to limited evidence, this finding should be interpreted with caution. Calcium, magnesium, zinc, and vitamin D may play roles in glucose metabolism, vascular function, inflammation regulation, and blood pressure control [95,96,97,98,99], all of which may be associated with the risk of cesarean section. However, no studies have yet directly confirmed these mechanisms. Therefore, the potential benefits of this combination supplement on cesarean section rates should be considered preliminary findings and require validation in future large-scale randomized controlled trials.

In this study, blueberry combined with dietary fiber and the magnesium–zinc–calcium–vitamin D complex supplement both yielded high SUCRA rankings for cesarean section rate, and plant sterols also showed a high SUCRA value for glycated hemoglobin; however, the evidence underpinning these findings is relatively weak. Although no significant global or local inconsistency was detected in most networks, clinical heterogeneity may still exist because the included studies differed in baseline BMI, intervention dose, treatment duration, and outcome measurement. In addition, the risk of bias should be considered. Although most trials were judged to have a low risk of bias, three studies were rated as having a high risk of bias, which may have affected the effect estimates and rankings, especially for interventions supported by few studies. Sensitivity analyses excluding these high-risk studies confirmed the overall robustness of the findings, although results based on sparse evidence should still be interpreted cautiously. Consequently, these conclusions should be regarded as exploratory and require further validation in future randomized controlled trials with adequate statistical power.

Our study has several limitations. First, some interventions, such as blueberry combined with dietary fiber and combined magnesium–zinc–calcium–vitamin D supplementation, were supported by only a few studies; therefore, their effect estimates and SUCRA rankings should be interpreted cautiously. Second, potential publication bias was detected for GDM incidence among high-risk pregnant women, which may have overestimated the pooled effects and rankings. Third, subgroup and dose–response analyses were limited by sparse evidence networks and variations in baseline BMI, gestational age, intervention dosage, formulation, duration, and timing. These factors may also have affected the transitivity assumption and the reliability of indirect comparisons. Fourth, exclusion of studies without available full texts may have introduced selection bias. Finally, the inclusion of only English-language publications and the geographical concentration of trials in Iran and China may have introduced language and regional bias and limited the generalizability of the findings. Future large-scale trials with standardized protocols are needed.

## 5. Conclusions

These findings suggest that inositol, the DASH diet, plant sterols, and fish oil may have potential benefits in the prevention or management of GDM. However, findings for interventions supported by limited evidence, such as blueberry combined with dietary fiber and combined magnesium–zinc–calcium–vitamin D supplementation, should be interpreted cautiously. Further high-quality, large-scale randomized controlled trials are needed to confirm these results.

## Figures and Tables

**Table 1:** Characteristics of high-risk pregnant women.

Author	Year	Country	Population	N (T)	N (C)	Age (T)	Age (C)	Intervention	Dose	Outcomes
Moini [64]	2025	Iran	High-risk pregnant women	71	74	32.81 ± 5.57	30.81 ± 6.22	Inositol	2000 mg, 2 times/day	A ↓, D ↓, E ↓
Chen [32]	2024	China	High-risk pregnant women	114	112	30 ± 4.51	30.65 ± 5.28	Inositol	500 mg, 2 times/day	A ↓, B →, E ↓
Asimakopoulos [27]	2024	Greece	High-risk pregnant women	94	98	33 ± 4.52	33.26 ± 5.46	Inositol	2000 mg, 2 times/day	A ↓, D ↓
Zhang [73]	2022	China	High-risk pregnant women	70	74	35.26 ± 1.8	35.19 ± 1.91	DF	24 g, 2 times/day	A →, B →, E →
Amaefule [22]	2022	UK	High-risk pregnant women	99	99	31.4 ± 5.8	31.9 ± 5.6	Inositol	2 g, 2 times/day	A ↓, B ↓
Esmaeilzadeh [39]	2021	Israel	High-risk pregnant women	27	29	27.8 ± 4.2	29.3 ± 4.4	Inositol	2000 mg, NA	A ↓, B →, D ↓
Shahriari [69]	2021	Iran	High-risk pregnant women	241	266	31.83 ± 5.8	32.20 ± 5.51	Probiotics	LA1, >7.5 × 10^9^ CFU; sp54cs, >1.5 × 10^9^ CFU; sp9cs, >6 × 10^9^ CFU; 1 time/day	A ↓, B ↓
Basu [28]	2021	USA	High-risk pregnant women	17	17	27 ± 5.3	27 ± 5.0	Blueberries + DF	Blueberries, 280 g; Soluble fiber, 12 g; 1 time/day	A ↓, B →, E →
Halkjær [43]	2020	Denmark	High-risk pregnant women	25	25	30.7 ± 4.5	30.7 ± 4.7	Probiotics	450 billion CFU, 1 time/day	A →, B →, D →, E →
Corcoy [14]	2020	Spain	High-risk pregnant women	79	75	32.8 ± 5.4	32.2 ± 5.2	Vitamin D	800 IU, 2 times/day	A →, B →, C →, D →, E →
Callaway [29]	2019	Australia	High-risk pregnant women	207	204	31.3 ± 4.7	31.7 ± 4.8	Probiotics	>1 × 10^9^ CFU, 1 time/day	A →, B →, E →
Pellonpera [66]	2019	Finland	High-risk pregnant women	328	110	30.4 ± 4.8 30.8 ± 4.8 30.8 ± 4.6	30.4 ± 4.1	Fish Oil Probiotics Fish Oil + Probiotics	Fish Oil, 2 capsules, 1 time/day Probiotics, 1 capsule, 1 time/day	A →, B →, D →
Celentano [31]	2018	USA	High-risk pregnant women	109	52	33.8 ± 4.3	33.9 ± 4.9	Inositol	MI, 4000 mg, DCI, 500 mg, MI/DCI, 27.6 mg DCI + 1100 mg MI, 2 times/day	A ↓, B →, D ↓
Wickens [71]	2017	New Zealand	High-risk pregnant women	212	211	33.3 ± 4.48	34 ± 4.48	Probiotics	HN001, 6 × 10^9^ CFU, 1 time/day	A ↓, B →
Markovic [60]	2016	Australia	High-risk pregnant women	65	60	36 ± 4.4	34.7 ± 4.1	LGD	Comprehensive Dietary Intervention	A →, B →
Santamaria [68]	2015	Italy	High-risk pregnant women	95	102	32.1 ± 4.8	32.7 ± 5.3	Inositol	2 g, 2 times/day	A ↓, B →, D →, E →
D’Anna [34]	2015	Italy	High-risk pregnant women	110	110	30.97 ± 19.53	31.21 ± 18.03	Inositol	2 g, 2 times/day	A ↓, B →, D ↓, E →
D’Anna [35]	2013	Italy	High-risk pregnant women	99	98	31.0 ± 5.3	31.6 ± 5.6	Inositol	2 g, 2 times/day	A ↓, B →, E →
Matarrelli [62]	2013	Italy	High-risk pregnant women	35	38	33.0 ± 4.9	33.8 ± 4.7	Inositol	2000 mg, 2 times/day	A ↓, D ↓
Zhou [75]	2012	China	High-risk pregnant women	1197	1202	28.9 ± 5.72	28.9 ± 5.6	Fish Oil	3 capsules, 1 time/day	A ↓
Walsh [70]	2012	Iran	High-risk pregnant women	383	398	32.0 ± 4.2	32.0 ± 4.2	LGD	Comprehensive Dietary Intervention	D →, E ↓

A: incidence rate of GDM; B: cesarean section rate; C: HOMA-IR; D: fasting blood glucose; E: gestational weight gain. NA: not reported or not available; ↓: compared to the control group, the intervention group demonstrated a more pronounced effect on this outcome measure; →: compared to the control group, the intervention showed no statistically significant effect on this outcome measure. MI: myo-inositol; DCI: D-chiro-inositol; DF: dietary fiber; LGD: low glycemic index diet.

**Table 2:** Characteristics of GDM patients.

Author	Year	Country	Population	N (T)	N (C)	Age (T)	Age (C)	Intervention	Dose	Outcomes
Kusinski [55]	2025	UK	GDM patients	214	211	33.26 ± 4.97	32.80 ± 5.11	RED	Comprehensive Dietary Intervention	A →, D →, E ↓
Nachum [65]	2024	Israel	GDM patients	41	44	33.8 ± 4.7	32.7 ± 5.1	Probiotics	>6 × 10^9^/capsule, 2 capsules/day	A →, D →
Nadeem [15]	2024	Pakistan	GDM patients	17	17	27.0 ± 1.7	25.24 ± 3.2	Vitamin D	200,000 IU, a single intramuscular dose	B ↓, C ↓, E ↓
Hernandez [44]	2023	USA	GDM patients	23	23	33 ± 1	32 ± 1	CHOICE	Comprehensive Dietary Intervention	A →, B →, C →, D →
Markussen [61]	2023	Finland	GDM patients	36	36	32.7 ± 4.5	32.7 ± 4.6	MCRD	Comprehensive Dietary Intervention	B →, C →
Camarena Pulido [30]	2022	Mexico	GDM patients	27	27	32 ± 6.5	32 ± 6.5	Vitamin D	5000 IU, 1 time/day	B →, E →
Amirani [23]	2022	Iran	GDM patients	26	25	27.1 ± 5.8	28.6 ± 3.8	Probiotics + Se	Probiotics, 2 × 10^9^ CFU; Se, 200 mg; 1 time/day	B ↓, C ↓, D →
D’Anna [36]	2021	Italy	GDM patients	60	59	34.3 ± 5.5	33.3 ± 6.4	Inositol + α-lactalbumin	Inositol, 2 g; α-lactalbumin, 50 mg; 2 times/day	A →, B ↓, D →
Wang [11]	2021	China	GDM patients	60	60	30.23 ± 3.78	30.47 ± 4.19	DF	18 g, 2 times/day	C ↓, D ↓, E ↓
Mijatovic [63]	2020	Australia	GDM patients	24	22	32.5 ± 0.9	34.2 ± 0.9	MLC	Comprehensive Dietary Intervention	A →, C →, E →
Jamilian [50]	2019	Iran	GDM patients	30	30	27.7 ± 4.0	29.1 ± 4.1	MgZnCaVD	Mg, 100 mg+, Zn, 4 mg+, Ca, 400 mg+, Vitamin D, 200 IU, 2 times/day	A ↓, C →, D →
Sahhaf Ebrahimi [67]	2019	Iran	GDM patients	42	42	31.64 ± 5.97	31.61 ± 5.49	Probiotics	*L. acidophilus* + *B. lactis*, 10^6^ CFU, 1 time/day	C ↓, E ↓
Kijmanawat [54]	2018	Thailand	GDM patients	28	29	32.50 ± 5.02	30.72 ± 5.05	Probiotics	*L. acidophilus*, 1000 million CFU, *Bifidobacterium bifidum*, 1000 million CFU, 1 time/day	B ↓, C ↓, D →
Jamilian [52]	2018	Iran	GDM patients	20	20	30.5 ± 3.8	30.8 ± 2.4	Fish Oil	1 capsule, 2 times/day	B ↓, C ↓, D →
Maktabi [59]	2018	Iran	GDM patients	30	30	30.1 ± 5.9	31.5 ± 3.2	Mg + Vitamin E	Mg, 250 mg, Vitamin E, 400 IU, 1 time/day	B ↓, C ↓, D →
Dilli [37]	2018	Turkey	GDM patients	52	68	30.9 ± 5.3	32.7 ± 5.9	Fish Oil	1 capsule, 2 times/day	A →, D →
Jamilian [51]	2017	Iran	GDM patients	105	35	31.5 ± 7.0 30.7 ± 3.5 31.2 ± 4.3	30.7 ± 4.1	Omega-3, Vitamin D, Omega-3 + Vitamin D	Omega-3, 1000 mg, 2 times/day; Vitamin D, 50,000 IU, 1 time/2 weeks;	B ↓, C ↓, D →
Jamilian [48]	2017	Iran	GDM patients	30	30	29.7 ± 5.5	30.4 ± 4.2	Omega-3 + Vitamin E	Omega-3, 1000 mg; Vitamin E, 400 IU; 1 time/day	A ↓, D →
Gao [40]	2017	China	GDM patients	123	121	29.8 ± 6.4	31.5 ± 5.2	Plant sterols	2 g, 2 times/day	B ↓, C ↓, D ↓, E ↓
Hernandez [13]	2016	USA	GDM patients	6	6	30 ± 1	28 ± 2	CHOICE	Comprehensive Dietary Intervention	A →, B ↓, C ↓, D →
Jafarnejad [47]	2016	Iran	GDM patients	41	41	31.9 ± 4.0	32.4 ± 3.1	Probiotics	*Lactic acid* bacteria, 112.5 × 10^9^ CFU, 2 times/day	B ↓, C ↓, D →, E →
Zhang [74]	2016	China	GDM patients	113	20	29.93 ± 4.82	29.8 ± 4.7	Vitamin D	200 IU, 1 time/day 50,000 IU, 1 time/month 50,000 IU 1 time/2 weeks	D →
Li [57]	2016	China	GDM patients	49	48	28.3 ± 4.1	29.0 ± 5.3	Vitamin D	500 IU, 2 times/day	B ↓, C ↓, D →
Karamali [53]	2016	Iran	GDM patients	30	30	31.8 ± 6.0	29.7 ± 4.0	Probiotics	*Acidophilus*, 2 × 10^9^ CFU/g, *L. Casei*, 2 × 10^9^ CFU/g, *B. bifidum*, 2 × 10^9^ CFU/g, 1 time/day	B ↓, C ↓, D →
Jamilian [49]	2016	Iran	GDM patients	30	30	28.4 ± 6.2	29.6 ± 4.3	Vitamin D + EPO	Vitamin D, 1000 IU, EPO, 1000 mg, 1 time/day	B ↓, C ↓, D →
Li [56]	2016	China	GDM patients	33	33	32.7 ± 4.9	30.8 ± 4.7	MNP	Comprehensive Dietary Intervention	A →, C ↓
Dolatkhah [38]	2015	Turkey	GDM patients	29	27	28.14 ± 6.24	26.48 ± 5.23	Probiotics	LA-5, BB-12, STY-31, LBY-27, >4 × 10^9^ CFU, 1 time/day	B ↓, C ↓
Yao [72]	2015	China	GDM patients	17	16	30.7 ± 5.6	28.3 ± 5.1	DASH	Comprehensive Dietary Intervention	A ↓, B ↓, C ↓, D →
Hu [46]	2015	China	GDM patients	66	64	29.7 ± 3.7	30.3 ± 4.9	LGD	Comprehensive Dietary Intervention	C ↓
Ma [12]	2014	China	GDM patients	41	42	30.1 ± 3.8	30.0 ± 3.5	LGD	Comprehensive Dietary Intervention	C ↓, E →
Asemi [25]	2014	Iran	GDM patients	26	26	31.9 ± 6.1	30.7 ± 6.3	DASH	Comprehensive Dietary Intervention	A ↓, D →
Asemi [24]	2014	Iran	GDM patients	22	23	31.1 ± 5.5	30.8 ± 6.2	Vitamin D	50,000 IU 1 time/3 weeks	A →, D →
Hernandez [45]	2014	USA	GDM patients	16	16	28.4 ± 1.0	28.4 ± 1.0	CHOICE	Comprehensive Dietary Intervention	C →
Ghanei [41]	2013	Iran	GDM patients	18	13	NA	NA	LGD + DF	DF, 15 g 3 times/day	C →
Asemi [26]	2012	Iran	GDM patients	17	17	30.7 ± 6.7	29.4 ± 6.2	DASH	Comprehensive Dietary Intervention	C →, D →, E →
Grant [42]	2011	Canada	GDM patients	24	23	34 ± 0.1	34 ± 1.1	LGD	Comprehensive Dietary Intervention	C →
Louie [58]	2011	Australia	GDM patients	47	45	34.0 ± 4.1	32.4 ± 4.5	LGD	Comprehensive Dietary Intervention	A →, C ↓, E →
Corrado [33]	2011	Italy	GDM patients	24	45	28.7 ± 3.5	28.4 ± 3.7	Inositol	2 g, 2 times/day	B ↓, C ↓

A: cesarean section rate; B: HOMA-IR; C: fasting blood glucose; D: gestational weight gain; E: HbA1c. NA: not reported or not available; ↓: compared to the control group, the intervention group demonstrated a more pronounced effect on this outcome measure; →: compared to the control group, the intervention showed no statistically significant effect on this outcome measure. RED: restricted energy diet; CHOICE: 60% carbohydrate, 25% fat, 15% protein; MCRD: carbohydrate-restricted diet; DF: dietary fiber; MLC: modestly lower carbohydrate diet; LGD: low glycemic index diet; MNP: macronutrient preload; DASH: Dietary Approaches to Stop Hypertension.

## Data Availability

No new data were created or analyzed in this study. Data sharing is not applicable to this article.

## Supplementary Materials

The following supporting information can be downloaded at https://www.mdpi.com/article/10.3390/healthcare14162593/s1. Table S1: PRISMA NMA Checklist; Table S2: Search strategy of the systematic review; Table S3: Risk of bias assessment for included randomized controlled clinical trials; Table S4: Rank Probability and SUCRA Values; Table S5: League Tables for Sensitive Analysis; Table S6: GRADE Assessment; Figure S1: Cumulative Rank Curve; Figure S2: Inconsistency analysis; Figure S3: Egger’s publication bias plot; Figure S4: Subgroup analysis of fasting blood glucose outcomes in the GDM population; Figure S5: Sensitivity analysis results excluding high-risk studies.

## Abbreviations

The following abbreviations are used in this manuscript:

BB	Blueberry
BMI	Body mass index
Ca	Calcium
CFU	Colony forming units
CHOICE	CHOICE diet
CI	Confidence interval
CSR	Cesarean section rate
DASH	Dietary Approaches to Stop Hypertension
DF	Dietary fiber
EPO	Evening primrose oil
FBG	Fasting blood glucose
FO	Fish oil
GDM	Gestational diabetes mellitus
GWG	Gestational weight gain
HbA1c	Glycated hemoglobin
HOMA-IR	Homeostatic Model Assessment for Insulin Resistance
IADPSG	International Association of Diabetes and Pregnancy Study Groups
Ins	Inositol
La	Alpha-lactalbumin
LGD	Low glycemic index diet
MCRD	Moderate carbohydrate high-fiber diet
Mg	Magnesium
MgZnCaVD	Magnesium–zinc–calcium–vitamin D complex
MLC	Modestly lower carbohydrate diet
MNP	Macronutrient preload
NMA	Network meta-analysis
Pb	Probiotics
PCOS	Polycystic ovary syndrome
PS	Plant sterols
RCTs	Randomized controlled trials
RED	Restricted energy diet
RR	Relative risk
Se	Selenium
SMD	Standardized mean difference
SUCRA	Surface under the cumulative ranking
VD	Vitamin D
VE	Vitamin E
