# Supplementary material for: Effects of Different Nutritional Strategies on the Prevention and Management of Gestational Diabetes Mellitus: A Systematic Review and Network Meta-Analysis
Source: Healthcare (Basel). 2026 Aug 18;14(16):2593. doi: 10.3390/healthcare14162593 (PMC13512817; doi:10.3390/healthcare14162593)

Supplementary materials

CONTENTS

Table S1: PRISMA NMA Checklist ***2-6***

Table S2: Search strategy of the systematic review ***7-17***

Table S3: Risk of bias assessment for included randomized controlled clinical trials ***18-20***

Table S4: Rank Probability and SUCRA Values ***21-29***

Table S5: League Tables for Sensitive Analysis ***30-44***

Table S6: GRADE Assessment ***45***

Figure S1: Cumulative Rank Curve ***46-54***

Figure S2: Inconsistency analysis ***55-63***

Figure S3: Egger's publication bias plot ***64-72***

Figure S4: Subgroup analysis of fasting blood glucose outcomes in the GDM population ***73-74***

Figure S5: Sensitivity analysis results excluding high-risk studies ***75-78***

Supplementary materials

Table S1: PRISMA NMA Checklist

Section/Topic	*Item #*	*Checklist Item*	*Page #*
**TITLE**			
Title	1	Identify the report as a systematic review *incorporating a network meta-analysis (or related form of meta-analysis).*	***1***
**ABSTRACT**			
Structured summary	2	Provide a structured summary including, as applicable:<br>**Background:** main objectives<br>**Methods:** data sources; study eligibility criteria, participants, and interventions; study appraisal; and *synthesis methods, such as network meta-analysis.*<br>**Results:** number of studies and participants identified; summary estimates with corresponding confidence/credible intervals; *treatment rankings may also be discussed. Authors may choose to summarize pairwise comparisons against a chosen treatment included in their analyses for brevity.*<br>**Discussion/Conclusions:** limitations; conclusions and implications of findings.<br>**Other:** primary source of funding; systematic review registration number with registry name.	***1-2***
**INTRODUCTION**			
Rationale	3	Describe the rationale for the review in the context of what is already known*, including mention of why a network meta-analysis has been conducted.*_	***2-3***
Objectives	4	Provide an explicit statement of questions being addressed, with reference to participants, interventions, comparisons, outcomes, and study design (PICOS).	***2-3***
**METHODS**			
Protocol and registration	5	Indicate whether a review protocol exists and if and where it can be accessed (e.g., Web address); and, if available, provide registration information, including registration number.	***3***
Eligibility criteria	6	Specify study characteristics (e.g., PICOS, length of follow-up) and report characteristics (e.g., years considered, language, publication status) used as criteria for eligibility, giving rationale. *Clearly describe eligible treatments included in the treatment network, and note whether any have been clustered or merged into the same node (with justification).*_	***3-4***
Information sources	7	Describe all information sources (e.g., databases with dates of coverage, contact with study authors to identify additional studies) in the search and date last searched.	***4***

(Continued)

Supplementary materials

PRISMA NMA Checklist (Continued)

Section/Topic	*Item #*	*Checklist Item*	*Page #*
Search	8	Present full electronic search strategy for at least one database, including any limits used, such that it could be repeated.	***3, Table S2***
Study selection	9	State the process for selecting studies (i.e., screening, eligibility, included in systematic review, and, if applicable, included in the meta-analysis).	***3-4***
Data collection process	10	Describe method of data extraction from reports (e.g., piloted forms, independently, in duplicate) and any processes for obtaining and confirming data from investigators.	***4***
Data items	11	List and define all variables for which data were sought (e.g., PICOS, funding sources) and any assumptions and simplifications made.	***4***
Geometry of the network	**S1**	Describe methods used to explore the geometry of the treatment network under study and potential biases related to it. This should include how the evidence base has been graphically summarized for presentation, and what characteristics were compiled and used to describe the evidence base to readers.	***4***
Risk of bias within individual studies	12	Describe methods used for assessing risk of bias of individual studies (including specification of whether this was done at the study or outcome level), and how this information is to be used in any data synthesis.	***4***
Summary measures	13	State the principal summary measures (e.g., risk ratio, difference in means). *Also describe the use of additional summary measures assessed, such as treatment rankings and surface under the cumulative ranking curve (SUCRA) values, as well as modified approaches used to present summary findings from meta-analyses.*	***4***
Planned methods of analysis	14	Describe the methods of handling data and combining results of studies for each network meta-analysis. This should include, but not be limited to:<br>• *Handling of multi-arm trials;*<br>• *Selection of variance structure;*<br>• *Selection of prior distributions in Bayesian analyses; and*<br>• *Assessment of model fit.*	***4***
Assessment of Inconsistency	**S2**	Describe the statistical methods used to evaluate the agreement of direct and indirect evidence in the treatment network(s) studied. Describe efforts taken to address its presence when found.	***4***

(Continued)

Supplementary materials

PRISMA NMA Checklist (Continued)

Section/Topic	***Item #***	***Checklist Item***	***Page #***
Risk of bias across studies	15	Specify any assessment of risk of bias that may affect the cumulative evidence (e.g., publication bias, selective reporting within studies).	***5***
Additional analyses	16	Describe methods of additional analyses if done, indicating which were pre-specified. This may include, but not be limited to, the following:<br>• Sensitivity or subgroup analyses;<br>• Meta-regression analyses;<br>• *Alternative formulations of the treatment network; and*<br>• *Use of alternative prior distributions for Bayesian analyses (if applicable).*_	***5***
**RESULTS†**			
Study selection	17	Give numbers of studies screened, assessed for eligibility, and included in the review, with reasons for exclusions at each stage, ideally with a flow diagram.	***5***
Presentation of network structure	**S3**	Provide a network graph of the included studies to enable visualization of the geometry of the treatment network.	***Fig.5,7***
Summary of network geometry	**S4**	Provide a brief overview of characteristics of the treatment network. This may include commentary on the abundance of trials and randomized patients for the different interventions and pairwise comparisons in the network, gaps of evidence in the treatment network, and potential biases reflected by the network structure.	***6***
Study characteristics	18	For each study, present characteristics for which data were extracted (e.g., study size, PICOS, follow-up period) and provide the citations.	***6***
Risk of bias within studies	19	Present data on risk of bias of each study and, if available, any outcome level assessment.	***12***
Results of individual studies	20	For all outcomes considered (benefits or harms), present, for each study: 1) simple summary data for each intervention group, and 2) effect estimates and confidence intervals. *Modified approaches may be needed to deal with information from larger networks.*	***13-19***

(Continued)

Supplementary materials

PRISMA NMA Checklist (Continued)

Section/Topic	***Item #***	***Checklist Item***	***Page #***
Synthesis of results	21	Present results of each meta-analysis done, including confidence/credible intervals. *In larger networks, authors may focus on comparisons versus a particular comparator (e.g. placebo or standard care), with full findings presented in an appendix. League tables and forest plots may be considered to summarize pairwise comparisons.* If additional summary measures were explored (such as treatment rankings), these should also be presented.	***13-19***
Exploration for inconsistency	**S5**	Describe results from investigations of inconsistency. This may include such information as measures of model fit to compare consistency and inconsistency models, *P* values from statistical tests, or summary of inconsistency estimates from different parts of the treatment network.	***13-19***
Risk of bias across studies	22	Present results of any assessment of risk of bias across studies for the evidence base being studied.	***12***
Results of additional analyses	23	Give results of additional analyses, if done (e.g., sensitivity or subgroup analyses, meta-regression analyses*, alternative network geometries studied, alternative choice of prior distributions for Bayesian analyses,* and so forth).	***12***
**DISCUSSION**			
Summary of evidence	24	Summarize the main findings, including the strength of evidence for each main outcome; consider their relevance to key groups (e.g., healthcare providers, users, and policy-makers).	***19-22***
Limitations	25	Discuss limitations at study and outcome level (e.g., risk of bias), and at review level (e.g., incomplete retrieval of identified research, reporting bias). *Comment on the validity of the assumptions, such as transitivity and consistency. Comment on any concerns regarding network geometry (e.g., avoidance of certain comparisons).*	***19-22***
Conclusions	26	Provide a general interpretation of the results in the context of other evidence, and implications for future research.	***19-22***

(Continued)

Supplementary materials

PRISMA NMA Checklist (Continued)

Section/Topic	***Item #***	***Checklist Item***	***Page #***
**FUNDING**			
Funding	27	Describe sources of funding for the systematic review and other support (e.g., supply of data); role of funders for the systematic review. This should also include information regarding whether funding has been received from manufacturers of treatments in the network and/or whether some of the authors are content experts with professional conflicts of interest that could affect use of treatments in the network.	***22***

PICOS = population, intervention, comparators, outcomes, study design.
** Text in italics indicate S wording specific to reporting of network meta-analyses that has been added to guidance from the PRISMA statement.*
† Authors may wish to plan for use of appendices to present all relevant information in full detail for items in this section.

Supplementary materials

Table S2: Search strategy of the systematic review

Cochrane

ID	Search Hits	Results
#1	MeSH descriptor: [Diabetes, Gestational] explode all trees	1737
#2	(Gestational Diabetes Mellitus OR Pregnancy Induced Diabetes OR Gestational Diabetes OR diabetes mellitus gravidarum OR diabetes mellitus of pregnancy OR diabetes of pregnancy OR pregnancy diabetes OR pregnancy diabetes mellitus):ti,ab,kw	7572
#3	#1 OR #2	7620
#4	MeSH descriptor: [Nutritional Support] explode all trees	4402
#5	MeSH descriptor: [Diet Therapy] explode all trees	8270
#6	MeSH descriptor: [Vitamins] explode all trees	5705
#7	MeSH descriptor: [Probiotics] explode all trees	3650
#8	MeSH descriptor: [Diet, Carbohydrate-Restricted] explode all trees	838
#9	MeSH descriptor: [Diet, Mediterranean] explode all trees	980
#10	MeSH descriptor: [Dietary Fiber] explode all trees	2664
#11	MeSH descriptor: [Fish Oils] explode all trees	4632
#12	(Nutritional Support OR Artificial Feeding):ti,ab,kw OR (Diet Therapies OR Dietary Modification* OR Diet Modification* OR Restriction Diet Therap* OR Restrictive Diet Therap* OR Dietary Restriction* OR 'diet intervention' OR 'diet treatment' OR 'dietary intervention' OR 'dietary therapy' OR 'dietary treatment' OR 'dietotherapy' OR 'nutrition therapy' OR 'nutritional therapy' OR 'diet therapy'):ti,ab,kw OR ('davitamin' OR 'vitaminology' OR 'vitamin*'):ti,ab,kw OR ('probiotic*'):ti,ab,kw OR (Carbohydrate Restricted Diet* OR Low Carbohydrate Diet* OR 'carbohydrate-poor diet' OR 'low carb diet'):ti,ab,kw	141254
#13	(Mediterranean Diet OR MedDiet):ti,ab,kw OR ('low GI diet' OR 'low GI treatment' OR 'low glycemic index therapy' OR 'low glycemic index treatment' OR 'reduced GI diet' OR 'low glycemic index diet'v):ti,ab,kw OR (Myoinositols OR inositol* OR myoinositol OR chiroinositol):ti,ab,kw OR ('alimentary fiber' OR 'diet fiber' OR 'dietary fibre' OR 'optifibre' OR 'stimulance multi fibre' OR 'dietary fiber' OR Wheat Bran* OR Roughage*):ti,ab,kw OR (Inositol* OR Fish Oil*):ti,ab,kw	17523
#14	#4 OR #5 OR #6 OR #7 OR #8 OR #9 OR #10 OR #11 OR #12 OR #13	150816
#15	#3 AND #14	2422

Pubmed

Search number	Query	Search Details	Results
5	((Diabetes, Gestational[MeSH Terms]) OR (Gestational Diabetes Mellitus[Title/Abstract] OR Pregnancy Induced Diabetes[Title/Abstract] OR Gestational Diabetes[Title/Abstract] OR diabetes mellitus gravidarum[Title/Abstract] OR diabetes mellitus of pregnancy[Title/Abstract] OR diabetes of pregnancy[Title/Abstract] OR pregnancy diabetes[Title/Abstract] OR pregnancy diabetes mellitus[Title/Abstract])) AND ((((((((Nutritional Support[MeSH Terms]) OR (Diet Therapy[MeSH Terms])) OR (Vitamins[MeSH Terms])) OR (Probiotics[MeSH Terms])) OR (Diet, Carbohydrate-Restricted[MeSH Terms])) OR (Diet, Mediterranean[MeSH Terms])) OR (Dietary Fiber[MeSH Terms])) OR (Fish Oils[MeSH Terms])) OR ((((((((((Nutritional Support[Title/Abstract] OR Artificial Feeding[Title/Abstract]) OR (Diet Therapies[Title/Abstract] OR Dietary Modification*[Title/Abstract] OR Diet	("diabetes, gestational"[MeSH Terms] OR ("gestational diabetes mellitus"[Title/Abstract] OR "pregnancy induced diabetes"[Title/Abstract] OR "gestational diabetes"[Title/Abstract] OR (("diabetes mellitus"[MeSH Terms] OR ("Diabetes"[All Fields] AND "Mellitus"[All Fields]) OR "diabetes mellitus"[All Fields]) AND "gravidarum"[Title/Abstract]) OR (("diabetes mellitus"[MeSH Terms] OR ("Diabetes"[All Fields] AND "Mellitus"[All Fields]) OR "diabetes mellitus"[All Fields]) AND "of pregnancy"[Title/Abstract]) OR (("diabete"[All Fields] OR "diabetes mellitus"[MeSH Terms] OR ("Diabetes"[All Fields] AND "Mellitus"[All Fields]) OR "diabetes mellitus"[All Fields] OR "Diabetes"[All Fields] OR "diabetes insipidus"[MeSH Terms] OR ("Diabetes"[All Fields] AND "insipidus"[All Fields]) OR "diabetes insipidus"[All Fields] OR "diabetic"[All Fields] OR "diabetics"[All Fields] OR "diabets"[All Fields]) AND "of pregnancy"[Title/Abstract]) OR "pregnancy diabetes"[Title/Abstract] OR "pregnancy diabetes mellitus"[Title/Abstract])) AND ("nutritional support"[MeSH Terms] OR "diet therapy"[MeSH Terms] OR "vitamins"[MeSH Terms] OR "probiotics"[MeSH Terms] OR "diet, carbohydrate restricted"[MeSH Terms] OR "diet, mediterranean"[MeSH Terms] OR "dietary fiber"[MeSH Terms] OR "fish oils"[MeSH Terms] OR	2412

Supplementary materials

	Modification*[Title/Abstract] OR Restriction Diet Therap*[Title/Abstract] OR Restrictive Diet Therap*[Title/Abstract] OR Dietary Restriction*[Title/Abstract] OR 'diet intervention'[Title/Abstract] OR 'diet treatment'[Title/Abstract] OR 'dietary intervention'[Title/Abstract] OR 'dietary therapy'[Title/Abstract] OR 'dietary treatment'[Title/Abstract] OR 'dietotherapy'[Title/Abstract] OR 'nutrition therapy'[Title/Abstract] OR 'nutritional therapy'[Title/Abstract] OR 'diet therapy'[Title/Abstract])) OR ('davitamin'[Title/Abstract] OR 'vitaminology'[Title/Abstract] OR 'vitamin*'[Title/Abstract])) OR ('probiotic*'[Title/Abstract])) OR (Carbohydrate Restricted Diet*[Title/Abstract] OR Low Carbohydrate Diet*[Title/Abstract] OR 'carbohydrate-poor diet'[Title/Abstract] OR 'low carb diet'[Title/Abstract])) OR (Mediterranean Diet[Title/Abstract] OR MedDiet[Title/Abstract])) OR ('low GI diet'[Title/Abstract] OR	("nutritional support"[Title/Abstract] OR "artificial feeding"[Title/Abstract] OR ("diet therapies"[Title/Abstract] OR "dietary modification*"[Title/Abstract] OR "diet modification*"[Title/Abstract] OR "restriction diet therap*"[Title/Abstract] OR "restrictive diet therap*"[Title/Abstract] OR "dietary restriction*"[Title/Abstract] OR "diet intervention"[Title/Abstract] OR "diet treatment"[Title/Abstract] OR "dietary intervention"[Title/Abstract] OR "dietary therapy"[Title/Abstract] OR "dietary treatment"[Title/Abstract] OR "dietotherapy"[Title/Abstract] OR "nutrition therapy"[Title/Abstract] OR "nutritional therapy"[Title/Abstract] OR "diet therapy"[Title/Abstract]) OR ("davitamin"[Title/Abstract] OR "vitaminology"[Title/Abstract] OR "vitamin*"[Title/Abstract]) OR "probiotic*"[Title/Abstract] OR ("carbohydrate restricted diet*"[Title/Abstract] OR "low carbohydrate diet*"[Title/Abstract] OR "carbohydrate poor diet"[Title/Abstract] OR "low carb diet"[Title/Abstract]) OR ("mediterranean diet"[Title/Abstract] OR "MedDiet"[Title/Abstract]) OR ("low gi diet"[Title/Abstract] OR "low gi treatment"[Title/Abstract] OR "low glycemic index therapy"[Title/Abstract] OR "low glycemic index treatment"[Title/Abstract] OR (("reduce"[All Fields] OR "reduced"[All Fields] OR "reduces"[All Fields] OR "reducing"[All Fields]) AND "gi diet"[Title/Abstract]) OR "low glycemic index diet"[Title/Abstract])

	'low GI treatment'[Title/Abstract] OR 'low glycemic index therapy'[Title/Abstract] OR 'low glycemic index treatment'[Title/Abstract] OR 'reduced GI diet'[Title/Abstract] OR 'low glycemic index diet'[Title/Abstract])) OR (Myoinositols[Title/Abstract] OR inositol*[Title/Abstract] OR myoinositol[Title/Abstract] OR chiroinositol[Title/Abstract])) OR ('alimentary fiber'[Title/Abstract] OR 'diet fiber'[Title/Abstract] OR 'dietary fibre'[Title/Abstract] OR 'optifibre'[Title/Abstract] OR 'stimulance multi fibre'[Title/Abstract] OR 'dietary fiber'[Title/Abstract] OR Wheat Bran*[Title/Abstract] OR Roughage*[Title/Abstract])) OR (Inositol*[Title/Abstract])) OR (Fish Oil*[Title/Abstract])))	OR ("Myoinositols"[Title/Abstract] OR "inositol*"[Title/Abstract] OR "myoinositol"[Title/Abstract] OR "chiroinositol"[Title/Abstract]) OR ("alimentary fiber"[Title/Abstract] OR "diet fiber"[Title/Abstract] OR "dietary fibre"[Title/Abstract] OR ("stimulance"[All Fields] AND "multi fibre"[Title/Abstract]) OR "dietary fiber"[Title/Abstract] OR "wheat bran*"[Title/Abstract] OR "roughage*"[Title/Abstract]) OR "inositol*"[Title/Abstract] OR "fish oil*"[Title/Abstract]))	
4	((((((((Nutritional Support[MeSH Terms]) OR (Diet Therapy[MeSH Terms])) OR (Vitamins[MeSH Terms])) OR (Probiotics[MeSH Terms])) OR (Diet, Carbohydrate-Restricted[MeSH Terms])) OR (Diet, Mediterranean[MeSH Terms])) OR (Dietary Fiber[MeSH Terms])) OR	"nutritional support"[MeSH Terms] OR "diet therapy"[MeSH Terms] OR "vitamins"[MeSH Terms] OR "probiotics"[MeSH Terms] OR "diet, carbohydrate restricted"[MeSH Terms] OR "diet, mediterranean"[MeSH Terms] OR "dietary fiber"[MeSH Terms] OR "fish oils"[MeSH Terms] OR ("nutritional support"[Title/Abstract] OR "artificial feeding"[Title/Abstract] OR ("diet therapies"[Title/Abstract] OR	597154

Supplementary materials

(Fish Oils[MeSH Terms])) OR (((((((((((Nutritional Support[Title/Abstract] OR Artificial Feeding[Title/Abstract]) OR (Diet Therapies[Title/Abstract] OR Dietary Modification*[Title/Abstract] OR Diet Modification*[Title/Abstract] OR Restriction Diet Therap*[Title/Abstract] OR Restrictive Diet Therap*[Title/Abstract] OR Dietary Restriction*[Title/Abstract] OR 'diet intervention'[Title/Abstract] OR 'diet treatment'[Title/Abstract] OR 'dietary intervention'[Title/Abstract] OR 'dietary therapy'[Title/Abstract] OR 'dietary treatment'[Title/Abstract] OR 'dietotherapy'[Title/Abstract] OR 'nutrition therapy'[Title/Abstract] OR 'nutritional therapy'[Title/Abstract] OR 'diet therapy'[Title/Abstract])) OR ('davitamin'[Title/Abstract] OR 'vitaminology'[Title/Abstract] OR 'vitamin*'[Title/Abstract])) OR ('probiotic*'[Title/Abstract])) OR (Carbohydrate Restricted Diet*[Title/Abstract] OR Low Carbohydrate	"dietary modification*"[Title/Abstract] OR "diet modification*"[Title/Abstract] OR "restriction diet therap*"[Title/Abstract] OR "restrictive diet therap*"[Title/Abstract] OR "dietary restriction*"[Title/Abstract] OR "diet intervention"[Title/Abstract] OR "diet treatment"[Title/Abstract] OR "dietary intervention"[Title/Abstract] OR "dietary therapy"[Title/Abstract] OR "dietary treatment"[Title/Abstract] OR "dietotherapy"[Title/Abstract] OR "nutrition therapy"[Title/Abstract] OR "nutritional therapy"[Title/Abstract] OR "diet therapy"[Title/Abstract]) OR ("davitamin"[Title/Abstract] OR "vitaminology"[Title/Abstract] OR "vitamin*"[Title/Abstract]) OR "probiotic*"[Title/Abstract] OR ("carbohydrate restricted diet*"[Title/Abstract] OR "low carbohydrate diet*"[Title/Abstract] OR "carbohydrate poor diet"[Title/Abstract] OR "low carb diet"[Title/Abstract]) OR ("mediterranean diet"[Title/Abstract] OR "MedDiet"[Title/Abstract]) OR ("low gi diet"[Title/Abstract] OR "low gi treatment"[Title/Abstract] OR "low glycemic index therapy"[Title/Abstract] OR "low glycemic index treatment"[Title/Abstract] OR (("reduce"[All Fields] OR "reduced"[All Fields] OR "reduces"[All Fields] OR "reducing"[All Fields]) AND "gi diet"[Title/Abstract]) OR "low glycemic index diet"[Title/Abstract]) OR ("Myoinositols"[Title/Abstract] OR "inositol*"[Title/Abstract] OR "myoinositol"[Title/Abstract] OR "chiroinositol"[Title/Abstract]) OR

	Diet*[Title/Abstract] OR 'carbohydrate-poor diet'[Title/Abstract] OR 'low carb diet'[Title/Abstract])) OR (Mediterranean Diet[Title/Abstract] OR MedDiet[Title/Abstract])) OR ('low GI diet'[Title/Abstract] OR 'low GI treatment'[Title/Abstract] OR 'low glycemic index therapy'[Title/Abstract] OR 'low glycemic index treatment'[Title/Abstract] OR 'reduced GI diet'[Title/Abstract] OR 'low glycemic index diet'[Title/Abstract])) OR (Myoinositols[Title/Abstract] OR inositol*[Title/Abstract] OR myoinositol[Title/Abstract] OR chiroinositol[Title/Abstract])) OR ('alimentary fiber'[Title/Abstract] OR 'diet fiber'[Title/Abstract] OR 'dietary fibre'[Title/Abstract] OR 'optifibre'[Title/Abstract] OR 'stimulance multi fibre'[Title/Abstract] OR 'dietary fiber'[Title/Abstract] OR Wheat Bran*[Title/Abstract] OR Roughage*[Title/Abstract])) OR (Inositol*[Title/Abstract])) OR (Fish Oil*[Title/Abstract]))	("alimentary fiber"[Title/Abstract] OR "diet fiber"[Title/Abstract] OR "dietary fibre"[Title/Abstract] OR ("stimulance"[All Fields] AND "multi fibre"[Title/Abstract]) OR "dietary fiber"[Title/Abstract] OR "wheat bran*"[Title/Abstract] OR "roughage*"[Title/Abstract]) OR "inositol*"[Title/Abstract] OR "fish oil*"[Title/Abstract])	
3	(((((((((Nutritional Support[Title/Abstract] OR Artificial	"nutritional support"[Title/Abstract] OR "artificial feeding"[Title/Abstract] OR ("diet	456485

	Feeding[Title/Abstract]) OR (Diet Therapies[Title/Abstract] OR Dietary Modification*[Title/Abstract] OR Diet Modification*[Title/Abstract] OR Restriction Diet Therap*[Title/Abstract] OR Restrictive Diet Therap*[Title/Abstract] OR Dietary Restriction*[Title/Abstract] OR 'diet intervention'[Title/Abstract] OR 'diet treatment'[Title/Abstract] OR 'dietary intervention'[Title/Abstract] OR 'dietary therapy'[Title/Abstract] OR 'dietary treatment'[Title/Abstract] OR 'dietotherapy'[Title/Abstract] OR 'nutrition therapy'[Title/Abstract] OR 'nutritional therapy'[Title/Abstract] OR 'diet therapy'[Title/Abstract])) OR ('davitamin'[Title/Abstract] OR 'vitaminology'[Title/Abstract] OR 'vitamin*'[Title/Abstract])) OR ('probiotic*'[Title/Abstract])) OR (Carbohydrate Restricted Diet*[Title/Abstract] OR Low Carbohydrate Diet*[Title/Abstract] OR 'carbohydrate-poor diet'[Title/Abstract] OR 'low carb	therapies"[Title/Abstract] OR "dietary modification*"[Title/Abstract] OR "diet modification*"[Title/Abstract] OR "restriction diet therap*"[Title/Abstract] OR "restrictive diet therap*"[Title/Abstract] OR "dietary restriction*"[Title/Abstract] OR "diet intervention"[Title/Abstract] OR "diet treatment"[Title/Abstract] OR "dietary intervention"[Title/Abstract] OR "dietary therapy"[Title/Abstract] OR "dietary treatment"[Title/Abstract] OR "dietotherapy"[Title/Abstract] OR "nutrition therapy"[Title/Abstract] OR "nutritional therapy"[Title/Abstract] OR "diet therapy"[Title/Abstract]) OR ("davitamin"[Title/Abstract] OR "vitaminology"[Title/Abstract] OR "vitamin*"[Title/Abstract]) OR "probiotic*"[Title/Abstract] OR ("carbohydrate restricted diet*"[Title/Abstract] OR "low carbohydrate diet*"[Title/Abstract] OR "carbohydrate poor diet"[Title/Abstract] OR "low carb diet"[Title/Abstract]) OR ("mediterranean diet"[Title/Abstract] OR "MedDiet"[Title/Abstract]) OR ("low gi diet"[Title/Abstract] OR "low gi treatment"[Title/Abstract] OR "low glycemic index therapy"[Title/Abstract] OR "low glycemic index treatment"[Title/Abstract] OR (("reduce"[All Fields] OR "reduced"[All Fields] OR "reduces"[All Fields] OR "reducing"[All Fields]) AND "gi diet"[Title/Abstract]) OR "low glycemic index diet"[Title/Abstract]) OR ("Myoinositols"[Title/Abstract] OR "inositol*"[Title/Abstract] OR "myoinositol"[Title/Abstract] OR

	diet'[Title/Abstract])) OR (Mediterranean Diet[Title/Abstract] OR MedDiet[Title/Abstract])) OR ('low GI diet'[Title/Abstract] OR 'low GI treatment'[Title/Abstract] OR 'low glycemic index therapy'[Title/Abstract] OR 'low glycemic index treatment'[Title/Abstract] OR 'reduced GI diet'[Title/Abstract] OR 'low glycemic index diet'[Title/Abstract])) OR (Myoinositols[Title/Abstract] OR inositol*[Title/Abstract] OR myoinositol[Title/Abstract] OR chiroinositol[Title/Abstract])) OR ('alimentary fiber'[Title/Abstract] OR 'diet fiber'[Title/Abstract] OR 'dietary fibre'[Title/Abstract] OR 'optifibre'[Title/Abstract] OR 'stimulance multi fibre'[Title/Abstract] OR 'dietary fiber'[Title/Abstract] OR Wheat Bran*[Title/Abstract] OR Roughage*[Title/Abstract])) OR (Inositol*[Title/Abstract])) OR (Fish Oil*[Title/Abstract])	"chiroinositol"[Title/Abstract]) OR ("alimentary fiber"[Title/Abstract] OR "diet fiber"[Title/Abstract] OR "dietary fibre"[Title/Abstract] OR ("stimulance"[All Fields] AND "multi fibre"[Title/Abstract]) OR "dietary fiber"[Title/Abstract] OR "wheat bran*"[Title/Abstract] OR "roughage*"[Title/Abstract]) OR "inositol*"[Title/Abstract] OR "fish oil*"[Title/Abstract]	
2	(((((((Nutritional Support[MeSH Terms]) OR (Diet Therapy[MeSH Terms])) OR (Vitamins[MeSH Terms])) OR (Probiotics[MeSH Terms])) OR (Diet,	"nutritional support"[MeSH Terms] OR "diet therapy"[MeSH Terms] OR "vitamins"[MeSH Terms] OR "probiotics"[MeSH Terms] OR "diet, carbohydrate restricted"[MeSH Terms] OR "diet, mediterranean"[MeSH Terms] OR	245830

	Carbohydrate-Restricted[MeSH Terms])) OR (Diet, Mediterranean[MeSH Terms])) OR (Dietary Fiber[MeSH Terms])) OR (Fish Oils[MeSH Terms])	"dietary fiber"[MeSH Terms] OR "fish oils"[MeSH Terms]	
1	(Diabetes, Gestational[MeSH Terms]) OR (Gestational Diabetes Mellitus[Title/Abstract] OR Pregnancy Induced Diabetes[Title/Abstract] OR Gestational Diabetes[Title/Abstract] OR diabetes mellitus gravidarum[Title/Abstract] OR diabetes mellitus of pregnancy[Title/Abstract] OR diabetes of pregnancy[Title/Abstract] OR pregnancy diabetes[Title/Abstract] OR pregnancy diabetes mellitus[Title/Abstract])	"diabetes, gestational"[MeSH Terms] OR ("gestational diabetes mellitus"[Title/Abstract] OR "pregnancy induced diabetes"[Title/Abstract] OR "gestational diabetes"[Title/Abstract] OR (("diabetes mellitus"[MeSH Terms] OR ("Diabetes"[All Fields] AND "Mellitus"[All Fields]) OR "diabetes mellitus"[All Fields]) AND "gravidarum"[Title/Abstract]) OR (("diabetes mellitus"[MeSH Terms] OR ("Diabetes"[All Fields] AND "Mellitus"[All Fields]) OR "diabetes mellitus"[All Fields]) AND "of pregnancy"[Title/Abstract]) OR (("diabete"[All Fields] OR "diabetes mellitus"[MeSH Terms] OR ("Diabetes"[All Fields] AND "Mellitus"[All Fields]) OR "diabetes mellitus"[All Fields] OR "Diabetes"[All Fields] OR "diabetes insipidus"[MeSH Terms] OR ("Diabetes"[All Fields] AND "insipidus"[All Fields]) OR "diabetes insipidus"[All Fields] OR "diabetic"[All Fields] OR "diabetics"[All Fields] OR "diabets"[All Fields]) AND "of pregnancy"[Title/Abstract]) OR "pregnancy diabetes"[Title/Abstract] OR "pregnancy diabetes mellitus"[Title/Abstract])	34298

Supplementary materials

EMBASE

No.	Query	Results
#6	#1 AND #4 AND #5	5389
#5	'randomized controlled trial'/exp OR 'randomized controlled trial' OR 'randomised controlled study':ab,ti OR 'randomised controlled trial':ab,ti OR 'randomized controlled study':ab,ti OR 'randomized controlled trial':ab,ti OR 'random*':ab,ti OR 'clinic*':ab,ti OR 'control':ab,ti OR 'trial':ab,ti OR 'placebo':ab,ti	13962046
#4	#2 OR #3	1570019
#3	'nutritional support':ab,ti OR 'artificial feeding':ab,ti OR 'diet therapies':ab,ti OR 'dietary modification*':ab,ti OR 'diet modification*':ab,ti OR 'restriction diet therap*':ab,ti OR 'restrictive diet therap*':ab,ti OR 'dietary restriction*':ab,ti OR 'diet intervention':ab,ti OR 'diet treatment':ab,ti OR 'dietary intervention':ab,ti OR 'dietary therapy':ab,ti OR 'dietary treatment':ab,ti OR 'dietotherapy':ab,ti OR 'nutrition therapy':ab,ti OR 'nutritional therapy':ab,ti OR 'diet therapy':ab,ti OR 'davitamin':ab,ti OR 'vitaminology':ab,ti OR 'vitamin*':ab,ti OR 'probiotic*':ab,ti OR 'carbohydrate restricted diet*':ab,ti OR 'low carbohydrate diet*':ab,ti OR 'carbohydrate-poor diet':ab,ti OR 'low carb diet':ab,ti OR 'mediterranean diet':ab,ti OR meddiet:ab,ti OR 'low gi diet':ab,ti OR 'low gi treatment':ab,ti OR 'low glycemic index therapy':ab,ti OR 'low glycemic index treatment':ab,ti OR 'reduced gi diet':ab,ti OR 'low glycemic index diet':ab,ti OR myoinositols:ab,ti OR myoinositol:ab,ti OR chiroinositol:ab,ti OR 'alimentary fiber':ab,ti OR 'diet fiber':ab,ti OR 'dietary fibre':ab,ti OR 'optifibre':ab,ti OR 'stimulance multi fibre':ab,ti OR 'dietary fiber':ab,ti OR 'wheat bran*':ab,ti OR roughage*:ab,ti OR inositol*:ab,ti OR 'fish oil*':ab,ti	588588
#2	'nutritional support'/exp OR 'nutritional support' OR 'diet therapy'/exp OR 'diet therapy' OR 'vitamin'/exp OR 'vitamin' OR 'probiotic agent'/exp OR 'probiotic agent' OR 'low carbohydrate diet'/exp OR 'low carbohydrate diet' OR 'mediterranean diet'/exp OR 'mediterranean diet' OR 'low glycemic index diet'/exp OR 'low glycemic index diet' OR 'dietary fiber'/exp OR 'dietary fiber'	1472329
#1	'gestational diabetes'/exp OR 'gestational diabetes' OR 'gestational diabetes mellitus':ab,ti OR 'pregnancy induced diabetes':ab,ti OR 'gestational diabetes':ab,ti OR	65943

Supplementary materials

No.	Query	Results
	'diabetes mellitus gravidarum':ab,ti OR 'diabetes mellitus of pregnancy':ab,ti OR 'diabetes of pregnancy':ab,ti OR 'pregnancy diabetes':ab,ti OR 'pregnancy diabetes mellitus':ab,ti	

Web of Science

No.	Query	Results
**#1**	Gestational Diabetes Mellitus OR Pregnancy Induced Diabetes OR Gestational Diabetes OR diabetes mellitus gravidarum OR diabetes mellitus of pregnancy OR diabetes of pregnancy OR pregnancy diabetes OR pregnancy diabetes mellitus (Topic)	**39642**
**#2**	Nutritional Support OR Artificial Feeding OR Diet Therapies OR Dietary Modification* OR Diet Modification* OR Restriction Diet Therap* OR Restrictive Diet Therap* OR Dietary Restriction* OR 'diet intervention' OR 'diet treatment' OR 'dietary intervention' OR 'dietary therapy' OR 'dietary treatment' OR 'dietotherapy' OR 'nutrition therapy' OR 'nutritional therapy' OR 'diet therapy' (Topic) or 'davitamin' OR 'vitaminology' OR 'vitamin*' OR 'probiotic*' OR Carbohydrate Restricted Diet* OR Low Carbohydrate Diet* OR 'carbohydrate-poor diet' OR 'low carb diet' (Topic) or Mediterranean Diet OR MedDiet OR 'low GI diet' OR 'low GI treatment' OR 'low glycemic index therapy' OR 'low glycemic index treatment' OR 'reduced GI diet' OR 'low glycemic index diet' (Topic) or Myoinositols OR inositol* OR myoinositol OR chiroinositol OR 'alimentary fiber' OR 'diet fiber' OR 'dietary fibre' OR 'optifibre' OR 'stimulance multi fibre' OR 'dietary fiber' OR Wheat Bran* OR Roughage* (Topic) or Inositol* OR Fish Oil* (Topic)	**768569**
**#3**	#1 AND #2	**4583**

Table S3: Risk of bias assessment for included randomized controlled clinical trials

+ Low risk
! Some concerns
— High risk

D1:Randomisation process;
D2:Deviations from the intended interventions;
D3:Missing outcome data;
D4:Measurement of the outcome;
D5:Selection of the reported result

Study ID	D1	D2	D3	D4	D5	Overall
Kusinski 2025	+	+	+	+	+	+
Moini 2025	+	+	+	+	+	+
Chen 2024	+	+	+	+	+	+
Nachum 2024	+	+	!	+	+	!
Asimakopoulos 2024	+	!	+	+	+	!
Nadeem 2024	+	!	+	+	!	!
Hernandez 2023	+	+	+	+	+	+
Markussen 2023	+	+	!	+	+	!
Zhang 2023	+	!	!	+	+	!
Pulido 2022	+	+	+	+	+	+
Amirani 2022	+	+	+	+	+	+
Amaefule 2022	+	+	+	+	+	+
Esmaeilzadeh 2021	+	+	+	+	+	+
D’Anna 2021	+	!	+	+	!	!
Wang 2021	+	!	+	+	!	!
Shahriari 2021	+	+	+	+	+	+
Basu 2021	+	—	+	+	+	—
Halkjær 2020	+	+	+	+	+	+

Table S4: Risk of bias assessment for included randomized controlled clinical trials (Continued)

Study ID	D1	D2	D3	D4	D5	Overall
Corcoy 2020	+	+	+	+	+	+
Mijatovic 2020	+	+	+	+	+	+
Pellonpera 2019	+	+	+	+	+	+
Jamilian 2019	+	+	+	+	+	+
Callaway 2019	+	+	+	+	+	+
Ebrahimi 2019	+	+	+	+	+	+
Celentano 2018	+	+	+	+	+	+
Kijmanawat 2018	+	+	+	+	+	+
Jamilian 2018	+	+	+	+	+	+
Maktabi 2018	+	+	+	+	+	+
Dilli 2018	+	–	–	+	+	–
Jamilian 2017	+	+	+	+	+	+
Jamilian 2017	+	+	+	+	+	+
Wickens 2017	+	+	+	+	+	+
Gao 2017	+	!	+	+	!	!
Hernandez 2016	+	+	+	+	+	+
Markovic 2016	+	+	+	+	+	+
Jafarnejad 2016	+	!	+	+	!	!
Zhang 2016	+	!	+	+	!	!
Jamilian 2016	+	+	+	+	+	+
Li 2016	+	+	–	+	!	–

Supplementary S4: Risk of bias assessment for included randomized controlled clinical trials (Continued)

Study ID	D1	D2	D3	D4	D5	Overall
Li 2016	+	!	+	+	!	!
Karamali 2016	+	+	+	+	+	+
Santamaria 2015	+	!	+	+	+	!
D'Anna 2015	+	!	+	!	+	!
Dolatkhah 2015	+	+	+	+	+	+
Yao 2015	+	+	+	+	!	!
Hu 2015	+	+	+	+	!	!
Ma 2014	+	+	+	+	!	!
Asemi 2014	+	!	+	+	!	!
Asemi 2014	+	+	+	+	+	+
Hernandez 2014	+	+	+	+	+	+
D'Anna 2013	+	!	+	+	+	!
Matarrelli 2013	+	+	+	+	+	+
Ghanei 2013	+	+	+	+	!	!
Asemi 2012	+	!	+	+	!	!
Zhou 2012	+	+	+	+	+	+
Walsh 2012	+	+	+	+	+	+
Grant 2011	+	+	+	+	+	+
Louie 2011	+	+	+	+	+	+
Corrado 2011	+	!	+	!	!	!

Supplementary materials

Table S4: Rank Probability and SUCRA Values

RED:Restricted Energy Diet; CHOICE:60% Carbohydrate,25% Fat,15% Protein; MCRD:Carbohydrate-Restricted Diet; DF:Dietary Fiber; MLC:Modestly lower Carbohydrate Diet; LGD:Low Glycemic Index Diet; MNP:Macronutrient Preload; DASH:Dietary Approaches to Stop Hypertension; BB:Blueberry; FO:Fish Oil; Pb:Probiotics; La:Alpha-Lactalbumin;Ins:Inositol; Se:Selenium; Mg:Magnesium; VE:Vitamin E; VD:Vitamin D; MgZnCaVD:Magnesium-Zinc-Calcium-Vitamin D; EPO:Evening Primrose Oil

High-Risk Populations of GDM

Incidence of GDM

Rank	BB+DF	Control	DF	FO	FO+Pb	Ins	LGD	Pb	VD
**1st**	31.7	0	23.2	1.3	0.9	26.1	10.9	3.2	2.7
**2nd**	12.9	0	18.8	3.6	3	38.4	11.1	6.1	6
**3rd**	11.5	1.2	16.8	6.9	5.7	24.7	11.7	10.8	10.6
**4th**	10.1	6	13	10.4	10.7	7.9	12.8	15.5	13.6
**5th**	6.4	19.3	7.8	11.5	13.1	2.4	10.7	15.4	13.3
**6th**	5.4	27.6	6	12.7	13.4	0.4	8.5	13.4	12.6
**7th**	4.9	26.9	4.5	14.6	16.8	0.1	8	12	12.1
**8th**	5.8	15	5.3	17.6	18.3	0	10.5	13.7	13.9
**Worst**	11.2	3.9	4.6	21.3	18	0	15.8	9.9	15.3
**SUCRA**	65.2	33.3	68.3	32.6	32.6	84.5	49.2	43.8	40.5

Fasting Blood Glucose

Rank	Control	FO	F0+Pb	Ins	LGD	Pb	VD
**1st**	0.0	1.6	1.8	55.5	12.3	3.5	25.3
**2nd**	3.0	3.6	4.2	33.4	21.2	8.7	26.0
**3rd**	20.3	7.1	8.4	8.6	22.8	13.9	18.8
**4th**	35.7	10.0	11.6	1.9	14.9	15.9	10.0
**5th**	22.7	16.4	19.7	0.5	11.3	21.9	7.6
**6th**	13.6	25.7	24.7	0.1	8.8	21.1	6.1
**Worst**	4.8	35.8	29.6	0.0	8.7	15.1	6.1
**SUCRA**	43.9	24.7	26.8	89.8	58.8	38.3	67.7

Supplementary materials

Gestational Weight Gain

Rank	BB+DF	Control	DF	Ins	LGD	Pb	VD
1st	33.9	0.0	43.2	0.0	16.0	1.8	5.2
2nd	20.1	0.0	29.1	0.7	32.3	6.4	11.4
3rd	15.1	1.8	15.9	5.0	30.0	15.8	16.4
4th	9.1	11.9	6.5	14.9	13.2	25.6	18.8
5th	5.2	32.2	2.9	23.7	5.2	18.9	12.0
6th	5.5	38.2	1.2	29.4	2.0	13.0	10.8
Worst	11.2	15.9	1.3	26.3	1.4	18.4	25.5
SUCRA	67.9	23.8	82.6	24.1	71.1	39.2	41.2

Cesarean Section Rate

Rank	BB+DF	Control	DF	FO	FO+Pb	Ins	LGD	Pb	VD
1st	93.5	0	0.1	1	0.2	1.4	0.5	3.3	0
2nd	3	0.4	2.9	12	2.8	39.8	10.2	28.5	0.4
3rd	1.1	13.6	9.1	12.3	6	33.3	10.8	13.1	0.7
4th	0.8	29.8	14.2	9.6	6.4	16.3	11.6	9.8	1.6
5th	0.3	28.3	21.9	9.3	6.7	7.4	13.7	8.9	3.4
6th	0.4	18.3	20.6	12.8	10.2	1.7	15.2	12.9	7.8
7th	0.3	8.2	17.8	17.2	16.4	0.2	15.7	11.2	13
8th	0.4	1.3	11	16.7	25.5	0	15.9	8.2	21.1
Worst	0.3	0	2.4	9.2	25.8	0	6.3	4.1	51.9
SUCRA	97.8	52.7	41.9	43	25.2	75.7	43.6	56.9	13.2

Supplementary materials

Patients with GDM

Fasting Blood Glucose

Rank	CHOICE	Control	DASH	DF	FO	Ins	LGD	LGD+DF	MCRD
**1st**	0.7	0.0	14.3	0.7	3.7	33.8	0.0	4.2	1.6
**2nd**	1.9	0.0	17.0	1.1	5.1	16.5	0.1	5.3	2.4
**3rd**	3.3	0.0	14.6	1.4	5.4	11.8	0.5	5.0	2.9
**4th**	4.0	0.0	10.4	2.3	5.3	7.7	1.3	4.9	3.7
**5th**	5.7	0.0	9.4	2.1	4.9	5.3	1.6	4.3	4.1
**6th**	6.4	0.0	6.5	2.6	5.1	4.5	2.7	4.3	3.8
**7th**	7.1	0.0	6.6	3.0	5.0	3.6	4.1	4.4	4.0
**8th**	8.1	0.0	4.7	3.0	4.7	2.9	5.6	4.3	4.5
**9th**	8.1	0.1	3.9	2.9	5.1	2.6	7.7	4.4	4.8
**10th**	8.6	0.4	3.5	3.6	5.0	2.1	8.5	4.7	4.3
**11th**	7.9	1.3	2.4	3.4	5.5	1.8	9.9	4.4	4.6
**12th**	7.4	3.8	2.1	4.5	4.9	1.6	9.8	4.7	5.1
**13th**	7.2	9.5	1.3	4.9	5.5	1.3	10.2	4.6	4.7
**14th**	6.2	15.4	1.1	5.0	4.0	0.9	9.5	4.6	4.9
**15th**	4.4	22.0	0.8	5.3	4.5	0.8	8.6	5.0	5.5
**16th**	4.6	21.6	0.7	6.3	4.7	0.8	6.6	4.4	5.8
**17th**	3.4	15.3	0.2	8.8	5.1	0.8	6.2	5.0	6.5
**18th**	2.6	8.0	0.3	10.0	5.7	0.6	4.4	6.3	8.0
**19th**	1.5	2.2	0.2	12.8	5.8	0.4	2.3	7.6	9.2
**Worst**	0.8	0.4	0.1	16.2	5.0	0.2	0.6	7.5	9.7
**SUCRA**	51.7	24.6	79.2	28.8	49.3	83.8	41.0	46.5	38.8

(continued)

Supplementary materials

Fasting Blood Glucose(continued)

Rank	MLC	MNP	Mg+VE	MgZnCaVD	Omega-3	PS	Pb
**1st**	0.8	0.4	4.5	3.7	1.7	15.9	0.2
**2nd**	0.8	0.8	5.7	4.8	4.3	13.6	0.7
**3rd**	1.4	1.3	5.7	5.7	4.2	10.6	2.0
**4th**	1.9	1.7	5.7	5.6	5.8	9.2	3.4
**5th**	2.0	2.0	5.7	5.4	6.5	7.8	5.6
**6th**	2.3	2.3	5.3	5.3	6.4	6.2	8.1
**7th**	2.0	2.4	5.5	5.4	5.8	5.3	8.7
**8th**	2.6	2.7	4.6	5.0	6.3	4.4	10.6
**9th**	2.6	3.4	5.7	5.1	6.5	4.1	10.6
**10th**	2.8	3.6	5.1	5.3	6.0	3.9	10.4
**11th**	4.1	4.2	5.9	5.0	5.1	3.1	10.0
**12th**	4.3	4.8	5.0	5.4	5.0	3.1	8.3
**13th**	4.3	5.6	4.6	4.4	5.1	2.8	6.5
**14th**	4.6	5.7	4.1	4.6	5.3	2.3	5.2
**15th**	4.9	6.1	4.0	4.2	4.3	1.8	4.2
**16th**	5.7	6.6	4.4	4.5	5.2	1.5	2.5
**17th**	7.6	8.6	4.6	5.0	5.1	1.6	1.6
**18th**	9.6	11.2	5.1	5.5	4.5	1.1	0.9
**19th**	14.8	13.0	5.3	5.2	3.6	1.1	0.4
**Worst**	21.0	13.6	3.7	4.8	3.2	0.7	0.1
**SUCRA**	25.9	28.3	52.1	50.3	51.0	73.3	54.3

(continued)

Supplementary materials

Fasting Blood Glucose(continued)

Rank	Se+Pb	VD	VD+EPO	VD+0mega-3
**1st**	3.1	1.7	2.6	6.4
**2nd**	4.4	4.4	3.4	7.7
**3rd**	4.6	7.2	3.9	8.4
**4th**	4.3	10.0	4.4	8.4
**5th**	4.6	11.1	4.8	7.2
**6th**	5.0	11.1	4.7	7.4
**7th**	5.0	10.4	4.9	6.9
**8th**	5.2	9.4	5.1	6.2
**9th**	5.0	7.4	4.5	5.3
**10th**	5.2	6.4	5.4	5.3
**11th**	5.8	5.9	5.1	4.8
**12th**	5.5	4.9	5.6	4.5
**13th**	5.0	3.4	5.1	4.1
**14th**	5.4	2.8	5.1	3.2
**15th**	4.7	1.5	4.4	3.0
**16th**	4.8	1.0	5.7	2.6
**17th**	5.5	0.8	5.7	2.6
**18th**	6.6	0.5	6.7	2.4
**19th**	5.6	0.2	6.7	2.1
**20th**	4.9	0.0	6.2	1.3
**Worst**	3.1	1.7	2.6	6.4
**SUCRA**	47.3	66.2	44.8	62.8

Supplementary materials

HOMA-IR

Rank	CHOICE	Control	DASH	Ins	Ins+La	MCRD	Mg+VE	Omega-3
**1st**	0.1	0.0	77.0	2.4	2.7	0.3	1.1	0.0
**2nd**	0.7	0.0	11.3	9.0	9.9	1.5	4.5	0.3
**3rd**	1.6	0.0	4.6	9.2	10.8	2.6	5.5	1.2
**4th**	2.8	0.0	2.7	10.8	10.3	3.8	6.5	2.4
**5th**	4.6	0.0	1.5	9.3	10.6	4.2	8.3	3.9
**6th**	6.0	0.0	1.0	9.0	9.7	5.0	8.8	6.4
**7th**	7.3	0.1	0.7	8.6	9.1	6.2	8.5	7.9
**8th**	8.6	0.2	0.5	7.2	7.6	7.2	8.2	11.3
**9th**	9.6	1.1	0.3	7.7	7.0	7.6	8.0	12.4
**10th**	11.6	5.0	0.2	7.1	5.8	8.6	8.2	13.4
**11th**	12.2	14.0	0.0	5.5	5.5	9.1	7.8	12.8
**12th**	10.5	30.8	0.1	4.6	4.3	9.1	7.0	10.6
**13th**	11.0	32.7	0.1	4.6	3.6	11.5	7.3	9.5
**Worst**	13.5	16.1	0.0	4.9	3.2	23.2	10.4	7.7
**SUCRA**	31.6	12.6	95.9	54.3	57.3	29.4	43.4	33.5

(continued)

HOMA-IR (continued)

Rank	PS	Pb	Se+Pb	VD	VD+EPO	VD+Omega-3
**1st**	8.9	3.7	1.1	0.0	1.6	1.0
**2nd**	25.6	20.4	4.9	0.2	6.3	5.4
**3rd**	16.1	25.6	6.5	1.2	7.5	7.5
**4th**	12.2	20.0	7.3	3.2	8.3	9.7
**5th**	9.8	13.7	8.3	5.8	9.8	10.2
**6th**	7.5	8.1	9.0	10.5	8.5	10.5
**7th**	5.7	4.1	8.5	13.6	8.8	10.7
**8th**	4.0	2.3	8.6	16.5	8.0	9.9
**9th**	3.4	1.1	8.7	15.9	8.4	8.7
**10th**	2.4	0.5	7.7	14.5	7.6	7.4
**11th**	1.9	0.3	7.5	9.7	6.8	6.9
**12th**	1.2	0.0	6.7	5.5	5.5	4.1
**13th**	0.8	0.0	6.7	2.5	5.5	4.2
**Worst**	0.6	0.0	8.7	0.9	7.3	3.6
**SUCRA**	74.5	78.1	45.5	42.9	49.1	51.9

Supplementary materials

Glycated Hemoglobin

Rank	Control	DASH	DF	LGD	MLC	PS	Pb	RED	VD
**1st**	0.0	19.7	0.3	0.0	6.8	72.4	0.2	0.2	0.4
**2nd**	0.0	37.9	5.1	0.0	21.4	22.3	5.0	2.6	5.7
**3rd**	0.0	19.4	12.4	0.6	25.0	4.5	15.7	7.5	14.9
**4th**	0.0	8.9	17.3	2.0	14.6	0.6	24.3	10.7	21.6
**5th**	0.5	5.8	19.0	5.4	10.8	0.1	23.6	13.8	20.9
**6th**	5.2	4.1	18.9	11.1	9.1	0.1	17.7	16.5	17.4
**7th**	23.9	2.3	13.1	18.5	5.6	0.0	8.7	16.6	11.3
**8th**	46.1	1.2	7.0	22.8	2.7	0.0	3.5	11.6	5.0
**Worst**	24.2	0.8	6.9	39.6	4.0	0.0	1.3	20.4	2.8
**SUCRA**	14	77.7	45.7	15.9	63.4	95.7	52.6	34.2	50.9

Gestational Weight Gain

Rank	CHOICE	Control	DASH	DF	FO	Ins+La	Mg+VE	MgZnCaVD	Omega-3	Ps
**1st**	1.0	0.0	1.4	2.7	53.6	6.6	17.2	3.9	1.8	0.0
**2nd**	1.9	0.0	4.5	7.2	24.6	14.0	16.8	6.1	4.0	0.3
**3rd**	3.0	0.0	7.4	10.7	10.6	15.1	12.7	7.0	5.8	1.4
**4th**	3.6	0.1	8.1	10.2	5.5	13.4	9.9	7.7	5.8	2.8
**5th**	3.1	1.1	8.3	10.0	2.3	10.8	8.0	6.5	7.2	3.6
**6th**	3.3	3.8	7.6	8.9	1.4	8.0	5.6	6.2	7.0	4.9
**7th**	3.0	8.7	8.2	7.7	0.8	5.7	3.7	5.5	5.8	6.2
**8th**	3.3	14.8	6.2	6.3	0.5	5.6	3.7	4.6	5.8	5.8
**9th**	3.3	20.5	5.7	5.2	0.2	4.3	3.3	3.9	4.8	7.0
**10th**	3.3	20.2	5.5	4.9	0.2	3.5	2.6	4.5	5.3	7.4
**11th**	3.8	15.3	5.7	4.8	0.2	2.6	2.5	4.1	5.1	9.2
**12th**	4.4	9.5	6.0	5.0	0.0	2.9	2.8	5.5	6.2	10.1
**13th**	6.1	4.3	6.0	4.5	0.1	2.3	2.5	5.7	6.6	11.0
**14th**	6.6	1.3	6.1	4.4	0.0	2.4	2.6	6.0	7.8	10.8
**15th**	10.4	0.3	5.3	3.4	0.0	1.2	2.2	6.6	8.8	9.9
**16th**	14.3	0.1	4.7	2.6	0.0	1.1	2.4	8.4	7.7	6.5
**Worst**	25.6	0.0	3.1	1.5	0.0	0.5	1.6	7.9	4.6	3.0
**SUCRA**	27.4	46.5	51.7	59.8	94.1	70.9	72.5	48.2	45.6	36.8

(continued)

Supplementary materials

Gestational Weight Gain(continued)

Rank	Pb	RED	Se+Pb	VD	VD+EPO	VD+Omega-3	VE+Omega-3
**1st**	0.0	0.1	4.9	0.1	4.0	0.3	2.3
**2nd**	0.2	1.0	7.1	0.7	6.3	1.2	4.3
**3rd**	1.2	2.8	7.3	1.4	6.7	1.9	5.1
**4th**	2.2	6.1	6.9	3.4	6.6	2.3	5.4
**5th**	4.4	8.7	5.9	5.2	6.7	3.3	4.9
**6th**	5.0	11.1	5.7	6.5	5.6	4.0	5.2
**7th**	5.6	12.3	5.6	8.4	4.4	3.7	4.9
**8th**	7.4	10.8	4.0	8.2	4.8	3.8	4.5
**9th**	7.6	9.2	4.0	9.2	4.3	3.8	3.7
**10th**	8.3	9.2	3.7	9.5	4.5	4.0	3.7
**11th**	9.9	8.9	3.7	10.2	4.7	4.9	4.5
**12th**	10.5	6.6	4.8	9.3	5.3	5.7	5.4
**13th**	10.8	5.4	5.3	10.0	5.6	8.1	5.6
**14th**	10.7	4.2	5.6	8.2	6.2	9.9	7.2
**15th**	8.1	2.4	7.1	5.9	7.4	11.8	8.9
**16th**	5.7	1.1	8.1	2.8	8.3	14.9	11.4
**Worst**	2.3	0.3	10.4	1.0	8.7	16.4	13.1
**SUCRA**	38.0	52.8	47.9	43.4	46.9	28.3	39.4

Supplementary materials

Cesarean Section Rate

Rank	CHOICE	Control	DASH	FO	Ins+La	LGD	MLC	MNP
**1st**	10.4	0.0	5.7	0.0	13.2	0.3	11.2	0.0
**2nd**	7.4	0.0	18.6	0.4	14.4	0.5	16.4	0.3
**3rd**	5.4	0.0	26.7	1.7	12.6	0.6	15.9	1.2
**4th**	3.9	0.0	24.6	5.3	10.4	1.1	13.9	2.2
**5th**	4.4	0.1	15.0	13.6	8.6	1.3	10.1	5.4
**6th**	3.7	0.5	5.8	21.2	6.0	1.8	8.7	8.1
**7th**	3.7	3.5	2.2	23.1	5.6	1.8	6.0	12.3
**8th**	3.1	14.2	0.9	18.7	4.9	2.5	4.2	15.0
**9th**	3.2	31.1	0.3	10.4	5.1	2.7	4.1	16.3
**10th**	5.1	34.1	0.1	4.0	7.3	5.0	4.6	20.4
**11th**	17.3	15.1	0.0	1.5	8.2	20.3	3.7	15.1
**12th**	17.4	1.5	0.0	0.1	3.3	41.0	1.1	3.6
**Worst**	15.0	0.0	0.0	0.0	0.5	21.2	0.1	0.0
**SUCRA**	39.5	29.5	78.5	51.3	63.9	14.8	69.7	37.0

(continued)

Cesarean Section Rate(continued)

Rank	MgZnCaVD	Omega3+ VE	Pb	RED	VD
**1st**	40.8	17.1	0.0	0.0	1.1
**2nd**	17.3	20.3	0.0	0.1	4.3
**3rd**	10.8	16.7	0.0	1.0	7.5
**4th**	8.0	12.9	0.0	4.2	13.6
**5th**	5.7	9.9	0.1	10.1	15.8
**6th**	4.3	6.6	0.1	19.6	13.7
**7th**	2.8	4.4	0.0	23.6	10.9
**8th**	2.0	3.8	0.0	21.1	9.7
**9th**	2.3	2.6	0.2	12.9	8.9
**10th**	2.6	3.0	0.5	5.4	7.9
**11th**	2.5	2.1	7.1	1.8	5.3
**12th**	0.8	0.6	29.1	0.2	1.3
**13th**	0.1	0.0	62.9	0.0	0.0
**14th**	3.0	0.9	0.4	27.9	0.2
**Worst**	0.0	0.1	0.1	63.8	0.0
**SUCRA**	82.1	75.5	3.9	48.8	55.4

Supplementary materials

Table S5: League Tables for Sensitive Analysis

RED:Restricted Energy Diet; CHOICE:60% Carbohydrate,25% Fat,15% Protein; MCRD:Carbohydrate-Restricted Diet; DF:Dietary Fiber; MLC:Modestly lower Carbohydrate Diet; LGD:Low Glycemic Index Diet; MNP:Macronutrient Preload; DASH:Dietary Approaches to Stop Hypertension; BB:Blueberry; FO:Fish Oil; Pb:Probiotics; La:Alpha-Lactalbumin;Ins:Inositol; Se:Selenium; Mg:Magnesium; VE:Vitamin E; VD:Vitamin D; MgZnCaVD:Magnesium-Zinc-Calcium-Vitamin D; EPO:Evening Primrose Oil

High-Risk Populations of GDM

Incidence of GDM(Big Loop)

VD	0.96 (0.32,2.88)	0.88 (0.22,3.44)	0.54 (0.23,1.26)	1.11 (0.37,3.31)	1.11 (0.37,3.29)	0.64 (0.18,2.29)	1.07 (0.48,2.38)	0.85 (0.35,2.04)
1.05 (0.35,3.15)	Pb	0.92 (0.24,3.52)	0.56 (0.25,1.27)	1.16 (0.54,2.46)	1.16 (0.40,3.34)	0.67 (0.19,2.34)	1.12 (0.52,2.39)	0.73 (0.12,4.45)
1.14 (0.29,4.46)	1.09 (0.28,4.17)	LGD	0.61 (0.19,1.92)	1.26 (0.33,4.80)	1.26 (0.33,4.77)	0.72 (0.16,3.22)	1.22 (0.40,3.69)	1.20 (0.28,5.15)
1.87 (0.80,4.38)	1.79 (0.79,4.04)	1.64 (0.52,5.16)	Ins	2.06 (0.92,4.64)	2.07 (0.93,4.60)	1.19 (0.42,3.38)	**2.00 (1.48,2.70)**	0.58 (0.12,2.91)
0.90 (0.30,2.71)	0.87 (0.41,1.84)	0.79 (0.21,3.03)	0.48 (0.22,1.09)	FO+Pb	1.00 (0.35,2.88)	0.58 (0.16,2.01)	0.97 (0.46,2.05)	0.58 (0.12,2.89)
0.90 (0.30,2.68)	0.86 (0.30,2.50)	0.79 (0.21,3.01)	0.48 (0.22,1.08)	1.00 (0.35,2.87)	FO	0.58 (0.17,2.00)	0.97 (0.46,2.03)	1.09 (0.38,3.14)
1.57 (0.44,5.65)	1.50 (0.43,5.28)	1.38 (0.31,6.14)	0.84 (0.30,2.39)	1.74 (0.50,6.07)	1.74 (0.50,6.04)	DF	1.68 (0.62,4.58)	1.01 (0.18,5.76)
0.93 (0.42,2.07)	0.89 (0.42,1.91)	0.82 (0.27,2.48)	**0.50 (0.37,0.68)**	1.03 (0.49,2.19)	1.03 (0.49,2.17)	0.59 (0.22,1.62)	Control	0.60 (0.14,2.50)
1.18 (0.49,2.83)	1.37 (0.22,8.30)	0.83 (0.19,3.57)	1.72 (0.34,8.62)	1.72 (0.35,8.58)	0.92 (0.32,2.64)	0.99 (0.17,5.65)	1.66 (0.40,6.92)	BB+DF

Supplementary materials

Fasting Blood Glucose(Big Loop)

VD	0.30 (-0.57,1.18)	0.10 (-0.74,0.93)	-0.19 (-0.87,0.48)	0.41 (-0.47,1.28)	0.43 (-0.44,1.31)	-0.08 (-0.68,0.53)
-0.30 (-1.18,0.57)	Pb	-0.21 (-1.02,0.61)	-0.50 (-1.15,0.15)	0.10 (-0.50,0.71)	0.13 (-0.47,0.73)	-0.22 (-1.20,0.76)
-0.10 (-0.93,0.74)	0.21 (-0.61,1.02)	LGD	-0.29 (-0.89,0.31)	0.31 (-0.51,1.13)	0.34 (-0.48,1.15)	0.13 (-0.42,0.68)
0.19 (-0.48,0.87)	0.50 (-0.15,1.15)	0.29 (-0.31,0.89)	Ins	0.60 (-0.05,1.25)	0.63 (-0.03,1.28)	**0.42 (0.18,0.66)**
-0.41 (-1.28,0.47)	-0.10 (-0.71,0.50)	-0.31 (-1.13,0.51)	-0.60 (-1.25,0.05)	FO+Pb	0.03 (-0.58,0.63)	-0.18 (-0.79,0.42)
-0.43 (-1.31,0.44)	-0.13 (-0.73,0.47)	-0.34 (-1.15,0.48)	-0.63 (-1.28,0.03)	-0.03 (-0.63,0.58)	FO	-0.21 (-0.81,0.40)
0.08 (-0.53,0.68)	0.22 (-0.76,1.20)	-0.13 (-0.68,0.42)	**-0.42 (-0.66,-0.18)**	0.18 (-0.42,0.79)	0.21 (-0.40,0.81)	Control

Supplementary materials

Gestational Weight Gain(Big Loop)

VD	0.01 (-0.60,0.62)	-0.23 (-0.85,0.39)	0.10 (-0.44,0.64)	-0.37 (-1.05,0.32)	0.09 (-0.41,0.59)	-0.26 (-1.17,0.64)
-0.01 (-0.62,0.60)	Pb	-0.24 (-0.74,0.25)	0.09 (-0.31,0.48)	-0.38 (-0.96,0.20)	0.08 (-0.26,0.42)	-0.27 (-1.10,0.55)
0.23 (-0.39,0.85)	0.24 (-0.25,0.74)	LGD	0.33 (-0.08,0.73)	-0.14 (-0.73,0.45)	0.32 (-0.04,0.68)	-0.03 (-0.87,0.80)
-0.10 (-0.64,0.44)	-0.09 (-0.48,0.31)	-0.33 (-0.73,0.08)	Ins	-0.47 (-0.97,0.04)	-0.01 (-0.20,0.19)	-0.36 (-1.14,0.42)
0.37 (-0.32,1.05)	0.38 (-0.20,0.96)	0.14 (-0.45,0.73)	0.47 (-0.04,0.97)	DF	0.46 (-0.01,0.93)	0.11 (-0.78,0.99)
-0.09 (-0.59,0.41)	-0.08 (-0.42,0.26)	-0.32 (-0.68,0.04)	0.01 (-0.19,0.20)	-0.46 (-0.93,0.01)	Control	-0.35 (-1.11,0.40)
0.26 (-0.64,1.17)	0.27 (-0.55,1.10)	0.03 (-0.80,0.87)	0.36 (-0.42,1.14)	-0.11 (-0.99,0.78)	0.35 (-0.40,1.11)	BB+DF

Supplementary materials

Cesarean Section Rate*(Big Loop)*

VD	0.64<br>(0.29,1.44)	0.72<br>(0.40,1.31)	**0.58<br>(0.38,0.90)**	0.88<br>(0.41,1.86)	0.74<br>(0.34,1.62)	0.73<br>(0.45,1.18)	0.67<br>(0.44,1.02)	0.31<br>(0.09,1.15)
1.55<br>(0.70,3.45)	Pb	1.12<br>(0.50,2.50)	0.90<br>(0.45,1.81)	1.36<br>(0.72,2.55)	1.15<br>(0.60,2.23)	1.13<br>(0.54,2.33)	1.04<br>(0.53,2.07)	0.98<br>(0.49,1.97)
1.38<br>(0.76,2.49)	0.89<br>(0.40,1.98)	LGD	0.81<br>(0.52,1.24)	1.21<br>(0.57,2.57)	1.03<br>(0.47,2.24)	1.00<br>(0.62,1.63)	0.93<br>(0.61,1.41)	**0.28<br>(0.09,0.91)**
**1.71<br>(1.11,2.64)**	1.11<br>(0.55,2.21)	1.24<br>(0.81,1.92)	Ins	1.50<br>(0.79,2.84)	1.28<br>(0.65,2.49)	1.25<br>(0.95,1.64)	**1.15<br>(1.03,1.30)**	0.35<br>(0.12,1.05)
1.14<br>(0.54,2.43)	0.74<br>(0.39,1.38)	0.83<br>(0.39,1.76)	0.67<br>(0.35,1.26)	FO+Pb	0.85<br>(0.47,1.55)	0.83<br>(0.42,1.63)	0.77<br>(0.41,1.44)	**0.23<br>(0.07,0.82)**
1.34<br>(0.62,2.92)	0.87<br>(0.45,1.67)	0.97<br>(0.45,2.12)	0.78<br>(0.40,1.53)	1.18<br>(0.64,2.14)	FO	0.98<br>(0.48,1.97)	0.90<br>(0.47,1.74)	**0.27<br>(0.08,0.98)**
1.38<br>(0.85,2.24	0.89<br>(0.43,1.84	1.00<br>(0.61,1.62	0.80<br>(0.61,1.06)	1.20<br>(0.61,2.37	1.02<br>(0.51,2.07	DF	0.93<br>(0.72,1.19)	**0.28<br>(0.09,0.86)**
1.49<br>(0.98,2.25)	0.96<br>(0.48,1.90)	1.08<br>(0.71,1.63)	**0.87<br>(0.77,0.97)**	1.30<br>(0.69,2.44)	1.11<br>(0.57,2.14)	1.08<br>(0.84,1.39)	Control	**0.30<br>(0.10,0.90)**
3.18<br>(0.87,11.60)	1.02<br>(0.51,2.03)	**3.57<br>(1.10,11.58)**	2.88<br>(0.95,8.69)	**4.32<br>(1.22,15.30)**	**3.67<br>(1.02,13.22)**	**3.58<br>(1.16,11.06)**	**3.32<br>(1.11,9.96)**	BB+DF

Supplementary materials

Patients with GDM

Fasting Blood Glucose(Big Loop)

VD+Omega-3	0.63<br>(-2.27,3.53)	-0.04<br>(-1.99,1.91)	0.52<br>(-2.39,3.43)	0.33<br>(-1.85,2.51)	-0.47<br>(-3.34,2.40)
-0.63<br>(-3.53,2.27)	VD+EPO	-0.67<br>(-3.15,1.81)	-0.11<br>(-3.15,2.93)	-0.30<br>(-2.65,2.05)	-1.10<br>(-4.11,1.90)
0.04<br>(-1.91,1.99)	0.67<br>(-1.81,3.15)	VD	0.56<br>(-1.93,3.05)	0.37<br>(-1.20,1.95)	-0.43<br>(-2.87,2.01)
-0.52<br>(-3.43,2.39)	0.11<br>(-2.93,3.15)	-0.56<br>(-3.05,1.93)	Se+Pb	-0.19<br>(-2.55,2.17)	-0.99<br>(-4.00,2.02)
-0.33<br>(-2.51,1.85)	0.30<br>(-2.05,2.65)	-0.37<br>(-1.95,1.20)	0.19<br>(-2.17,2.55)	Pb	-0.80<br>(-3.11,1.51)
0.47<br>(-2.40,3.34)	1.10<br>(-1.90,4.11)	0.43<br>(-2.01,2.87)	0.99<br>(-2.02,4.00)	0.80<br>(-1.51,3.11)	PS
-0.42<br>(-2.56,1.71)	0.21<br>(-2.69,3.11)	-0.46<br>(-2.41,1.49)	0.10<br>(-2.81,3.00)	-0.09<br>(-2.27,2.08)	-0.89<br>(-3.76,1.97)
-0.44<br>(-3.34,2.46)	0.19<br>(-2.84,3.22)	-0.48<br>(-2.96,2.00)	0.08<br>(-2.96,3.12)	-0.11<br>(-2.46,2.24)	-0.91<br>(-3.92,2.09)
-0.37<br>(-3.27,2.53)	0.26<br>(-2.77,3.30)	-0.41<br>(-2.89,2.07)	0.15<br>(-2.89,3.19)	-0.04<br>(-2.39,2.31)	-0.84<br>(-3.85,2.16)
-1.26<br>(-3.88,1.35)	-0.63<br>(-3.53,2.27)	-1.30<br>(-3.26,0.65)	-0.74<br>(-3.65,2.17)	-0.93<br>(-3.11,1.25)	-1.73<br>(-4.60,1.13)
-1.39<br>(-4.33,1.56)	-0.76<br>(-3.83,2.32)	-1.43<br>(-3.96,1.11)	-0.87<br>(-3.95,2.22)	-1.06<br>(-3.47,1.35)	-1.86<br>(-4.91,1.19)
-0.88<br>(-3.77,2.01)	-0.25<br>(-3.27,2.78)	-0.92<br>(-3.39,1.55)	-0.36<br>(-3.39,2.67)	-0.55<br>(-2.89,1.79)	-1.35<br>(-4.34,1.64)
-0.57<br>(-3.70,2.55)	0.06<br>(-3.19,3.31)	-0.61<br>(-3.36,2.13)	-0.05<br>(-3.31,3.21)	-0.24<br>(-2.87,2.39)	-1.04<br>(-4.27,2.18)
-0.73<br>(-2.95,1.50)	-0.09<br>(-2.49,2.30)	-0.77<br>(-2.41,0.87)	-0.21<br>(-2.61,2.20)	-0.40<br>(-1.83,1.04)	-1.20<br>(-3.55,1.16)
1.01<br>(-1.91,3.93)	1.64<br>(-1.41,4.69)	0.97<br>(-1.54,3.47)	1.53<br>(-1.53,4.59)	1.34<br>(-1.04,3.71)	0.54<br>(-2.49,3.56)
-0.46<br>(-3.38,2.46)	0.17<br>(-2.88,3.23)	-0.50<br>(-3.01,2.01)	0.06<br>(-3.00,3.13)	-0.13<br>(-2.51,2.25)	-0.93<br>(-3.96,2.10)
-1.24<br>(-4.12,1.63)	-0.61<br>(-3.62,2.40)	-1.28<br>(-3.73,1.17)	-0.72<br>(-3.74,2.30)	-0.91<br>(-3.23,1.41)	-1.71<br>(-4.69,1.27)
0.62<br>(-1.91,3.14)	1.25<br>(-1.43,3.93)	0.58<br>(-1.45,2.60)	1.14<br>(-1.55,3.82)	0.95<br>(-0.92,2.82)	0.15<br>(-2.49,2.79)
-1.16<br>(-3.11,0.80)	-0.52<br>(-2.67,1.62)	-1.19<br>(-2.44,0.05)	-0.63<br>(-2.79,1.52)	-0.82<br>(-1.79,0.14)	-1.63<br>(-3.73,0.48)
-0.39<br>(-2.76,1.97)	0.24<br>(-2.29,2.77)	-0.43<br>(-2.26,1.39)	0.13<br>(-2.41,2.66)	-0.06<br>(-1.71,1.58)	-0.86<br>(-3.35,1.63)

Supplementary materials

Fasting Blood Glucose(Big Loop)(continued)

0.42<br>(-1.71,2.56)	0.44<br>(-2.46,3.34)	0.37<br>(-2.53,3.27)	1.26<br>(-1.35,3.88)	1.39<br>(-1.56,4.33)	0.88<br>(-2.01,3.77)	0.57<br>(-2.55,3.70)	0.73<br>(-1.50,2.95)
-0.21<br>(-3.11,2.69)	-0.19<br>(-3.22,2.84)	-0.26<br>(-3.30,2.77)	0.63<br>(-2.27,3.53)	0.76<br>(-2.32,3.83)	0.25<br>(-2.78,3.27)	-0.06<br>(-3.31,3.19)	0.09<br>(-2.30,2.49)
0.46<br>(-1.49,2.41)	0.48<br>(-2.00,2.96)	0.41<br>(-2.07,2.89)	1.30<br>(-0.65,3.26)	1.43<br>(-1.11,3.96)	0.92<br>(-1.55,3.39)	0.61<br>(-2.13,3.36)	0.77<br>(-0.87,2.41)
-0.10<br>(-3.00,2.81)	-0.08<br>(-3.12,2.96)	-0.15<br>(-3.19,2.89)	0.74<br>(-2.17,3.65)	0.87<br>(-2.22,3.95)	0.36<br>(-2.67,3.39)	0.05<br>(-3.21,3.31)	0.21<br>(-2.20,2.61)
0.09<br>(-2.08,2.27)	0.11<br>(-2.24,2.46)	0.04<br>(-2.31,2.39)	0.93<br>(-1.25,3.11)	1.06<br>(-1.35,3.47)	0.55<br>(-1.79,2.89)	0.24<br>(-2.39,2.87)	0.40<br>(-1.04,1.83)
0.89<br>(-1.97,3.76)	0.91<br>(-2.09,3.92)	0.84<br>(-2.16,3.85)	1.73<br>(-1.13,4.60)	1.86<br>(-1.19,4.91)	1.35<br>(-1.64,4.34)	1.04<br>(-2.18,4.27)	1.20<br>(-1.16,3.55)
Omega-3	0.02<br>(-2.88,2.92)	-0.05<br>(-2.95,2.85)	0.84<br>(-1.77,3.46)	0.97<br>(-1.98,3.91)	0.46<br>(-2.43,3.35)	0.15<br>(-2.98,3.28)	0.30<br>(-1.92,2.53)
-0.02<br>(-2.92,2.88)	MgZnCaVD	-0.07<br>(-3.10,2.97)	0.82<br>(-2.08,3.72)	0.95<br>(-2.13,4.03)	0.44<br>(-2.59,3.46)	0.13<br>(-3.12,3.39)	0.28<br>(-2.11,2.68)
0.05<br>(-2.85,2.95)	0.07<br>(-2.97,3.10)	Mg+VE	0.89<br>(-2.01,3.79)	1.02<br>(-2.06,4.10)	0.51<br>(-2.52,3.53)	0.20<br>(-3.05,3.46)	0.35<br>(-2.04,2.75)
-0.84<br>(-3.46,1.77)	-0.82<br>(-3.72,2.08)	-0.89<br>(-3.79,2.01)	MNP	0.13<br>(-2.82,3.07)	-0.38<br>(-3.28,2.51)	-0.69<br>(-3.82,2.44)	-0.54<br>(-2.76,1.69)
-0.97<br>(-3.91,1.98)	-0.95<br>(-4.03,2.13)	-1.02<br>(-4.10,2.06)	-0.13<br>(-3.07,2.82)	MLC	-0.51<br>(-3.58,2.56)	-0.81<br>(-4.11,2.48)	-0.66<br>(-3.11,1.79)
-0.46<br>(-3.35,2.43)	-0.44<br>(-3.46,2.59)	-0.51<br>(-3.53,2.52)	0.38<br>(-2.51,3.28)	0.51<br>(-2.56,3.58)	MCRD	-0.31<br>(-3.55,2.94)	-0.15<br>(-2.54,2.23)
-0.15<br>(-3.28,2.98)	-0.13<br>(-3.39,3.12)	-0.20<br>(-3.46,3.05)	0.69<br>(-2.44,3.82)	0.81<br>(-2.48,4.11)	0.31<br>(-2.94,3.55)	LGD+DF	0.15<br>(-2.05,2.35)
-0.30<br>(-2.53,1.92)	-0.28<br>(-2.68,2.11)	-0.35<br>(-2.75,2.04)	0.54<br>(-1.69,2.76)	0.66<br>(-1.79,3.11)	0.15<br>(-2.23,2.54)	-0.15<br>(-2.35,2.05)	LGD
1.43<br>(-1.49,4.35)	1.45<br>(-1.60,4.50)	1.38<br>(-1.67,4.43)	2.27<br>(-0.65,5.19)	2.40<br>(-0.70,5.49)	1.89<br>(-1.16,4.93)	1.58<br>(-1.69,4.85)	1.73<br>(-0.69,4.15)
-0.04<br>(-2.96,2.89)	-0.02<br>(-3.08,3.04)	-0.09<br>(-3.15,2.97)	0.80<br>(-2.12,3.73)	0.93<br>(-2.17,4.03)	0.42<br>(-2.63,3.47)	0.11<br>(-3.16,3.39)	0.27<br>(-2.16,2.69)
-0.82<br>(-3.69,2.05)	-0.80<br>(-3.81,2.21)	-0.87<br>(-3.88,2.14)	0.02<br>(-2.85,2.90)	0.15<br>(-2.91,3.20)	-0.36<br>(-3.36,2.64)	-0.67<br>(-3.90,2.56)	-0.52<br>(-2.88,1.85)
1.04<br>(-1.48,3.56)	1.06<br>(-1.62,3.74)	0.99<br>(-1.69,3.67)	1.88<br>(-0.64,4.40)	2.01<br>(-0.72,4.73)	1.50<br>(-1.17,4.16)	1.19<br>(-1.73,4.11)	1.34<br>(-0.58,3.27)
-0.73<br>(-2.68,1.22)	-0.71<br>(-2.86,1.43)	-0.78<br>(-2.93,1.36)	0.11<br>(-1.84,2.06)	0.23<br>(-1.97,2.44)	-0.28<br>(-2.41,1.86)	-0.58<br>(-3.03,1.86)	-0.43<br>(-1.50,0.64)
0.03<br>(-2.34,2.39)	0.05<br>(-2.48,2.58)	-0.02<br>(-2.55,2.51)	0.87<br>(-1.50,3.24)	1.00<br>(-1.59,3.58)	0.49<br>(-2.03,3.00)	0.18<br>(-2.61,2.97)	0.33<br>(-1.38,2.04)

Supplementary materials

Fasting Blood Glucose(Big Loop)(continued)

-1.01<br>(-3.93,1.91)	0.46<br>(-2.46,3.38)	1.24<br>(-1.63,4.12)	-0.62<br>(-3.14,1.91)	1.16<br>(-0.80,3.11)	0.39<br>(-1.97,2.76)
-1.64<br>(-4.69,1.41)	-0.17<br>(-3.23,2.88)	0.61<br>(-2.40,3.62)	-1.25<br>(-3.93,1.43)	0.52<br>(-1.62,2.67)	-0.24<br>(-2.77,2.29)
-0.97<br>(-3.47,1.54)	0.50<br>(-2.01,3.01)	1.28<br>(-1.17,3.73)	-0.58<br>(-2.60,1.45)	1.19<br>(-0.05,2.44)	0.43<br>(-1.39,2.26)
-1.53<br>(-4.59,1.53)	-0.06<br>(-3.13,3.00)	0.72<br>(-2.30,3.74)	-1.14<br>(-3.82,1.55)	0.63<br>(-1.52,2.79)	-0.13<br>(-2.66,2.41)
-1.34<br>(-3.71,1.04)	0.13<br>(-2.25,2.51)	0.91<br>(-1.41,3.23)	-0.95<br>(-2.82,0.92)	0.82<br>(-0.14,1.79)	0.06<br>(-1.58,1.71)
-0.54<br>(-3.56,2.49)	0.93<br>(-2.10,3.96)	1.71<br>(-1.27,4.69)	-0.15<br>(-2.79,2.49)	1.63<br>(-0.48,3.73)	0.86<br>(-1.63,3.35)
-1.43<br>(-4.35,1.49)	0.04<br>(-2.89,2.96)	0.82<br>(-2.05,3.69)	-1.04<br>(-3.56,1.48)	0.73<br>(-1.22,2.68)	-0.03<br>(-2.39,2.34)
-1.45<br>(-4.50,1.60)	0.02<br>(-3.04,3.08)	0.80<br>(-2.21,3.81)	-1.06<br>(-3.74,1.62)	0.71<br>(-1.43,2.86)	-0.05<br>(-2.58,2.48)
-1.38<br>(-4.43,1.67)	0.09<br>(-2.97,3.15)	0.87<br>(-2.14,3.88)	-0.99<br>(-3.67,1.69)	0.78<br>(-1.36,2.93)	0.02<br>(-2.51,2.55)
-2.27<br>(-5.19,0.65)	-0.80<br>(-3.73,2.12)	-0.02<br>(-2.90,2.85)	-1.88<br>(-4.40,0.64)	-0.11<br>(-2.06,1.84)	-0.87<br>(-3.24,1.50)
-2.40<br>(-5.49,0.70)	-0.93<br>(-4.03,2.17)	-0.15<br>(-3.20,2.91)	-2.01<br>(-4.73,0.72)	-0.23<br>(-2.44,1.97)	-1.00<br>(-3.58,1.59)
-1.89<br>(-4.93,1.16)	-0.42<br>(-3.47,2.63)	0.36<br>(-2.64,3.36)	-1.50<br>(-4.16,1.17)	0.28<br>(-1.86,2.41)	-0.49<br>(-3.00,2.03)
-1.58<br>(-4.85,1.69)	-0.11<br>(-3.39,3.16)	0.67<br>(-2.56,3.90)	-1.19<br>(-4.11,1.73)	0.58<br>(-1.86,3.03)	-0.18<br>(-2.97,2.61)
-1.73<br>(-4.15,0.69)	-0.27<br>(-2.69,2.16)	0.52<br>(-1.85,2.88)	-1.34<br>(-3.27,0.58)	0.43<br>(-0.64,1.50)	-0.33<br>(-2.04,1.38)
Ins	1.47<br>(-1.61,4.54)	2.25<br>(-0.78,5.28)	0.39<br>(-2.31,3.09)	2.16<br>(-0.01,4.33)	1.40<br>(-1.15,3.95)
-1.47<br>(-4.54,1.61)	FO	0.78<br>(-2.25,3.82)	-1.08<br>(-3.78,1.63)	0.70<br>(-1.48,2.87)	-0.07<br>(-2.62,2.49)
-2.25<br>(-5.28,0.78)	-0.78<br>(-3.82,2.25)	DF	-1.86<br>(-4.51,0.79)	-0.09<br>(-2.20,2.02)	-0.85<br>(-3.35,1.65)
-0.39<br>(-3.09,2.31)	1.08<br>(-1.63,3.78)	1.86<br>(-0.79,4.51)	DASH	**1.77<br>(0.17,3.37）**	1.01<br>(-1.07,3.09)
-2.16<br>(-4.33,0.01)	-0.70<br>(-2.87,1.48)	0.09<br>(-2.02,2.20)	**-1.77<br>(-3.37,-0.17）**	Control	-0.76<br>(-2.10,0.58)
-1.40<br>(-3.95,1.15)	0.07<br>(-2.49,2.62)	0.85<br>(-1.65,3.35)	-1.01<br>(-3.09,1.07)	0.76<br>(-0.58,2.10)	CHOICE

HOMA-IR

VD+Omega-3	0.04<br>(-1.91,1.99)	0.21<br>(-1.08,1.51)	0.14<br>(-1.82,2.10)	-0.67<br>(-2.15,0.81)	-0.70<br>(-2.60,1.20)
-0.04<br>(-1.99,1.91)	VD+EPO	0.18<br>(-1.47,1.82)	0.10<br>(-1.99,2.20)	-0.70<br>(-2.36,0.95)	-0.74<br>(-2.77,1.30)
-0.21<br>(-1.51,1.08)	-0.18<br>(-1.82,1.47)	VD	-0.07<br>(-1.73,1.58)	-0.88<br>(-1.93,0.17)	-0.91<br>(-2.50,0.67)
-0.14<br>(-2.10,1.82)	-0.10<br>(-2.20,1.99)	0.07<br>(-1.58,1.73)	Se+Pb	-0.81<br>(-2.48,0.86)	-0.84<br>(-2.89,1.21)
0.67<br>(-0.81,2.15)	0.70<br>(-0.95,2.36)	0.88<br>(-0.17,1.93)	0.81<br>(-0.86,2.48)	Pb	-0.03<br>(-1.63,1.56)
0.70<br>(-1.20,2.60)	0.74<br>(-1.30,2.77)	0.91<br>(-0.67,2.50)	0.84<br>(-1.21,2.89)	0.03<br>(-1.56,1.63)	PS
-0.42<br>(-1.77,0.94)	-0.38<br>(-2.16,1.40)	-0.20<br>(-1.30,0.89)	-0.28<br>(-2.07,1.51)	-1.08<br>(-2.33,0.16)	-1.12<br>(-2.84,0.60)
-0.20<br>(-2.14,1.75)	-0.16<br>(-2.24,1.93)	0.02<br>(-1.62,1.66)	-0.05<br>(-2.15,2.04)	-0.86<br>(-2.52,0.79)	-0.90<br>(-2.93,1.14)
-0.59<br>(-2.52,1.35)	-0.55<br>(-2.62,1.52)	-0.37<br>(-2.00,1.25)	-0.44<br>(-2.53,1.64)	-1.25<br>(-2.89,0.39)	-1.29<br>(-3.31,0.74)
0.18<br>(-1.73,2.09)	0.22<br>(-1.84,2.27)	0.39<br>(-1.21,2.00)	0.32<br>(-1.74,2.39)	-0.49<br>(-2.10,1.13)	-0.52<br>(-2.52,1.49)
0.09<br>(-1.85,2.04)	0.13<br>(-1.95,2.22)	0.31<br>(-1.33,1.95)	0.24<br>(-1.86,2.33)	-0.57<br>(-2.23,1.08)	-0.61<br>(-2.64,1.43)
1.94<br>(-0.18,4.06)	1.98<br>(-0.27,4.23)	**2.15<br>(0.30,4.00)**	2.08<br>(-0.18,4.34)	1.27<br>(-0.59,3.13)	1.24<br>(-0.97,3.45)
-0.85<br>(-2.13,0.42)	-0.82<br>(-2.29,0.66)	-0.64<br>(-1.37,0.09)	-0.71<br>(-2.20,0.78)	**-1.52<br>(-2.28,-0.77)**	**-1.55<br>(-2.96,-0.15)**
-0.49<br>(-2.20,1.22)	-0.45<br>(-2.31,1.42)	-0.27<br>(-1.63,1.08)	-0.34<br>(-2.22,1.53)	-1.15<br>(-2.52,0.22)	-1.19<br>(-3.00,0.63)

(continued)

HOMA-IR(Big Loop)(continued)

0.42<br>(-0.94,1.77)	0.20<br>(-1.75,2.14)	0.59<br>(-1.35,2.52)	-0.18<br>(-2.09,1.73)	-0.09<br>(-2.04,1.85)	-1.94<br>(-4.06,0.18)	0.85<br>(-0.42,2.13)	0.49<br>(-1.22,2.20)
0.38<br>(-1.40,2.16)	0.16<br>(-1.93,2.24)	0.55<br>(-1.52,2.62)	-0.22<br>(-2.27,1.84)	-0.13<br>(-2.22,1.95)	-1.98<br>(-4.23,0.27)	0.82<br>(-0.66,2.29)	0.45<br>(-1.42,2.31)
0.20<br>(-0.89,1.30)	-0.02<br>(-1.66,1.62)	0.37<br>(-1.25,2.00)	-0.39<br>(-2.00,1.21)	-0.31<br>(-1.95,1.33)	**-2.15**<br>**(-4.00,-0.30)**	0.64<br>(-0.09,1.37)	0.27<br>(-1.08,1.63)
0.28<br>(-1.51,2.07)	0.05<br>(-2.04,2.15)	0.44<br>(-1.64,2.53)	-0.32<br>(-2.39,1.74)	-0.24<br>(-2.33,1.86)	-2.08<br>(-4.34,0.18)	0.71<br>(-0.78,2.20)	0.34<br>(-1.53,2.22)
1.08<br>(-0.16,2.33)	0.86<br>(-0.79,2.52)	1.25<br>(-0.39,2.89)	0.49<br>(-1.13,2.10)	0.57<br>(-1.08,2.23)	-1.27<br>(-3.13,0.59)	**1.52**<br>**(0.77,2.28)**	1.15<br>(-0.22,2.52)
1.12<br>(-0.60,2.84)	0.90<br>(-1.14,2.93)	1.29<br>(-0.74,3.31)	0.52<br>(-1.49,2.52)	0.61<br>(-1.43,2.64)	-1.24<br>(-3.45,0.97)	**1.55**<br>**(0.15,2.96)**	1.19<br>(-0.63,3.00)
Omega-3	-0.22<br>(-2.00,1.55)	0.17<br>(-1.59,1.93)	-0.60<br>(-2.34,1.14)	-0.51<br>(-2.29,1.26)	**-2.36**<br>**(-4.33,-0.39)**	0.44<br>(-0.56,1.43)	0.07<br>(-1.45,1.58)
0.22<br>(-1.55,2.00)	Mg+VE	0.39<br>(-1.68,2.46)	-0.38<br>(-2.43,1.68)	-0.29<br>(-2.37,1.79)	-2.14<br>(-4.38,0.11)	0.66<br>(-0.81,2.13)	0.29<br>(-1.57,2.15)
-0.17<br>(-1.93,1.59)	-0.39<br>(-2.46,1.68)	MCRD	-0.77<br>(-2.81,1.27)	-0.68<br>(-2.75,1.39)	**-2.53**<br>**(-4.76,-0.29)**	0.27<br>(-1.19,1.72)	-0.10<br>(-1.95,1.75)
0.60<br>(-1.14,2.34)	0.38<br>(-1.68,2.43)	0.77<br>(-1.27,2.81)	Ins+La	0.09<br>(-1.97,2.14)	-1.76<br>(-3.98,0.46)	1.03<br>(-0.40,2.46)	0.67<br>(-1.16,2.50)
0.51<br>(-1.26,2.29)	0.29<br>(-1.79,2.37)	0.68<br>(-1.39,2.75)	-0.09<br>(-2.14,1.97)	Ins	-1.85<br>(-4.09,0.40)	0.95<br>(-0.52,2.42)	0.58<br>(-1.28,2.44)
**2.36**<br>**(0.39,4.33)**	2.14<br>(-0.11,4.38)	**2.53**<br>**(0.29,4.76)**	1.76<br>(-0.46,3.98)	1.85<br>(-0.40,4.09)	DASH	**2.79**<br>**(1.09,4.49)**	**2.43**<br>**(0.38,4.47)**
-0.44<br>(-1.43,0.56)	-0.66<br>(-2.13,0.81)	-0.27<br>(-1.72,1.19)	-1.03<br>(-2.46,0.40)	-0.95<br>(-2.42,0.52)	**-2.79**<br>**(-4.49,-1.09)**	Control	-0.37<br>(-1.51,0.78)
-0.07<br>(-1.58,1.45)	-0.29<br>(-2.15,1.57)	0.10<br>(-1.75,1.95)	-0.67<br>(-2.50,1.16)	-0.58<br>(-2.44,1.28)	**-2.43**<br>**(-4.47,-0.38)**	0.37<br>(-0.78,1.51)	CHOICE

Supplementary materials

Glycated Hemoglobin(Big Loop)

VD	0.23 (-0.69,1.16)	-0.02 (-0.78,0.73)	**-0.96 (-1.79,-0.14)**	-0.25 (-1.31,0.82)	0.46 (-0.29,1.20)	0.06 (-0.80,0.92)	-0.52 (-1.57,0.54)	0.44 (-0.13,1.01)
-0.23 (-1.16,0.69)	RED	-0.26 (-1.14,0.62)	**-1.19 (-2.14,-0.25)**	-0.48 (-1.64,0.68)	0.22 (-0.65,1.10)	-0.17 (-1.14,0.80)	-0.75 (-1.90,0.40)	0.21 (-0.52,0.93)
0.02 (-0.73,0.78)	0.26 (-0.62,1.14)	Pb	**-0.94 (-1.71,-0.16)**	-0.22 (-1.25,0.80)	0.48 (-0.21,1.17)	0.09 (-0.72,0.89)	-0.50 (-1.51,0.52)	0.46 (-0.03,0.96)
**0.96 (0.14,1.79)**	**1.19 (0.25,2.14)**	**0.94 (0.16,1.71)**	PS	0.71 (-0.37,1.79)	**1.42 (0.65,2.19)**	**1.02 (0.15,1.90)**	0.44 (-0.63,1.51)	**1.40 (0.80,2.00)**
0.25 (-0.82,1.31)	0.48 (-0.68,1.64)	0.22 (-0.80,1.25)	-0.71 (-1.79,0.37)	MLC	0.71 (-0.31,1.72)	0.31 (-0.79,1.41)	-0.27 (-1.53,0.99)	0.69 (-0.21,1.59)
-0.46 (-1.20,0.29)	-0.22 (-1.10,0.65)	-0.48 (-1.17,0.21)	**-1.42 (-2.19,-0.65)**	-0.71 (-1.72,0.31)	LGD	-0.40 (-1.20,0.41)	-0.98 (-1.99,0.03)	-0.02 (-0.50,0.46)
-0.06 (-0.92,0.80)	0.17 (-0.80,1.14)	-0.09 (-0.89,0.72)	**-1.02 (-1.90,-0.15)**	-0.31 (-1.41,0.79)	0.40 (-0.41,1.20)	DF	-0.58 (-1.67,0.51)	0.38 (-0.26,1.02)
0.52 (-0.54,1.57)	0.75 (-0.40,1.90)	0.50 (-0.52,1.51)	-0.44 (-1.51,0.63)	0.27 (-0.99,1.53)	0.98 (-0.03,1.99)	0.58 (-0.51,1.67)	DASH	**0.96 (0.07,1.85)**
-0.44 (-1.01,0.13)	-0.21 (-0.93,0.52)	-0.46 (-0.96,0.03)	**-1.40 (-2.00,-0.80)**	-0.69 (-1.59,0.21)	0.02 (-0.46,0.50)	-0.38 (-1.02,0.26)	**-0.96 (-1.85,-0.07)**	Control

Supplementary materials

Gestational Weight Gain*(Big Loop)*

VE+Omega-3	0.08<br>(-0.58,0.74)	-0.06<br>(-0.77,0.66)	-0.04<br>(-0.60,0.51)	-0.06<br>(-0.81,0.69)	-0.09<br>(-0.64,0.45)
-0.08<br>(-0.74,0.58)	VD+Omega-3	-0.13<br>(-0.79,0.52)	-0.12<br>(-0.54,0.30)	-0.13<br>(-0.83,0.56)	-0.17<br>(-0.64,0.30)
0.06<br>(-0.66,0.77)	0.13<br>(-0.52,0.79)	VD+EPO	0.01<br>(-0.54,0.57)	0.00<br>(-0.75,0.75)	-0.04<br>(-0.58,0.51)
0.04<br>(-0.51,0.60)	0.12<br>(-0.30,0.54)	-0.01<br>(-0.57,0.54)	VD	-0.01<br>(-0.61,0.58)	-0.05<br>(-0.36,0.26)
0.06<br>(-0.69,0.81)	0.13<br>(-0.56,0.83)	-0.00<br>(-0.75,0.75)	0.01<br>(-0.58,0.61)	Se+Pb	-0.04<br>(-0.62,0.55)
0.09<br>(-0.45,0.64)	0.17<br>(-0.30,0.64)	0.04<br>(-0.51,0.58)	0.05<br>(-0.26,0.36)	0.04<br>(-0.55,0.62)	RED
0.01<br>(-0.55,0.57)	0.09<br>(-0.39,0.57)	-0.05<br>(-0.60,0.51)	-0.03<br>(-0.36,0.30)	-0.05<br>(-0.64,0.55)	-0.08<br>(-0.39,0.22)
0.00<br>(-0.56,0.57)	0.08<br>(-0.41,0.57)	-0.05<br>(-0.62,0.51)	-0.04<br>(-0.38,0.30)	-0.05<br>(-0.66,0.55)	-0.09<br>(-0.41,0.23)
0.05<br>(-0.61,0.71)	0.13<br>(-0.34,0.60)	-0.01<br>(-0.67,0.65)	0.01<br>(-0.41,0.43)	-0.01<br>(-0.70,0.69)	-0.04<br>(-0.51,0.42)
0.07<br>(-0.64,0.79)	0.15<br>(-0.51,0.81)	0.01<br>(-0.70,0.73)	0.03<br>(-0.53,0.59)	0.01<br>(-0.73,0.76)	-0.02<br>(-0.57,0.52)
0.28<br>(-0.44,0.99)	0.35<br>(-0.31,1.01)	0.22<br>(-0.50,0.93)	0.23<br>(-0.33,0.79)	0.22<br>(-0.53,0.97)	0.18<br>(-0.36,0.73)
0.24<br>(-0.38,0.86)	0.31<br>(-0.24,0.87)	0.18<br>(-0.44,0.80)	0.19<br>(-0.24,0.62)	0.18<br>(-0.48,0.83)	0.14<br>(-0.27,0.55)
0.50<br>(-0.10,1.09)	**0.57**<br>**(0.04,1.10)**	0.44<br>(-0.16,1.04)	**0.45**<br>**(0.06,0.85)**	0.44<br>(-0.20,1.07)	**0.40**<br>**(0.03,0.78)**
0.15<br>(-0.47,0.77)	0.22<br>(-0.33,0.78)	0.09<br>(-0.53,0.71)	0.10<br>(-0.33,0.53)	0.09<br>(-0.57,0.74)	0.05<br>(-0.36,0.46)
0.09<br>(-0.53,0.71)	0.17<br>(-0.39,0.72)	0.03<br>(-0.59,0.65)	0.05<br>(-0.38,0.48)	0.03<br>(-0.62,0.69)	-0.00<br>(-0.41,0.41)
0.06<br>(-0.45,0.57)	0.13<br>(-0.29,0.56)	0.00<br>(-0.51,0.51)	0.01<br>(-0.22,0.25)	0.00<br>(-0.55,0.55)	-0.04<br>(-0.23,0.16)
-0.11<br>(-0.84,0.61)	-0.04<br>(-0.70,0.63)	-0.17<br>(-0.89,0.55)	-0.16<br>(-0.72,0.41)	-0.17<br>(-0.92,0.58)	-0.21<br>(-0.76,0.35)

(continued)

Supplementary materials

Gestational Weight Gain*(Big Loop)(continued)*

-0.01<br>(-0.57,0.55)	-0.00<br>(-0.57,0.56)	-0.05<br>(-0.71,0.61)	-0.07<br>(-0.79,0.64)	-0.28<br>(-0.99,0.44)	-0.24<br>(-0.86,0.38)	-0.50<br>(-1.09,0.10)	-0.15<br>(-0.77,0.47)
-0.09<br>(-0.57,0.39)	-0.08<br>(-0.57,0.41)	-0.13<br>(-0.60,0.34)	-0.15<br>(-0.81,0.51)	-0.35<br>(-1.01,0.31)	-0.31<br>(-0.87,0.24)	**-0.57<br>(-1.10,-0.04)**	-0.22<br>(-0.78,0.33)
0.05<br>(-0.51,0.60)	0.05<br>(-0.51,0.62)	0.01<br>(-0.65,0.67)	-0.01<br>(-0.73,0.70)	-0.22<br>(-0.93,0.50)	-0.18<br>(-0.80,0.44)	-0.44<br>(-1.04,0.16)	-0.09<br>(-0.71,0.53)
0.03<br>(-0.30,0.36)	0.04<br>(-0.30,0.38)	-0.01<br>(-0.43,0.41)	-0.03<br>(-0.59,0.53)	-0.23<br>(-0.79,0.33)	-0.19<br>(-0.62,0.24)	**-0.45<br>(-0.85,-0.06)**	-0.10<br>(-0.53,0.33)
0.05<br>(-0.55,0.64)	0.05<br>(-0.55,0.66)	0.01<br>(-0.69,0.70)	-0.01<br>(-0.76,0.73)	-0.22<br>(-0.97,0.53)	-0.18<br>(-0.83,0.48)	-0.44<br>(-1.07,0.20)	-0.09<br>(-0.74,0.57)
0.08<br>(-0.22,0.39)	0.09<br>(-0.23,0.41)	0.04<br>(-0.42,0.51)	0.02<br>(-0.52,0.57)	-0.18<br>(-0.73,0.36)	-0.14<br>(-0.55,0.27)	**-0.40<br>(-0.78,-0.03)**	-0.05<br>(-0.46,0.36)
Pb	0.01<br>(-0.34,0.35)	-0.04<br>(-0.52,0.44)	-0.06<br>(-0.62,0.50)	-0.26<br>(-0.82,0.29)	-0.22<br>(-0.65,0.20)	**-0.49<br>(-0.88,-0.09)**	-0.13<br>(-0.56,0.29)
-0.01<br>(-0.35,0.34)	PS	-0.05<br>(-0.54,0.44)	-0.07<br>(-0.63,0.50)	-0.27<br>(-0.84,0.29)	-0.23<br>(-0.67,0.21)	**-0.49<br>(-0.90,-0.09)**	-0.14<br>(-0.58,0.30)
0.04<br>(-0.44,0.52)	0.05<br>(-0.44,0.54)	Omega-3	-0.02<br>(-0.68,0.64)	-0.23<br>(-0.89,0.44)	-0.19<br>(-0.74,0.37)	-0.45<br>(-0.97,0.08)	-0.10<br>(-0.65,0.46)
0.06<br>(-0.50,0.62)	0.07<br>(-0.50,0.63)	0.02<br>(-0.64,0.68)	MgZnCaVD	-0.21<br>(-0.92,0.51)	-0.17<br>(-0.79,0.46)	-0.43<br>(-1.02,0.17)	-0.08<br>(-0.70,0.54)
0.26<br>(-0.29,0.82)	0.27<br>(-0.29,0.84)	0.23<br>(-0.44,0.89)	0.21<br>(-0.51,0.92)	Mg+VE	0.04<br>(-0.58,0.66)	-0.22<br>(-0.82,0.38)	0.13<br>(-0.49,0.75)
0.22<br>(-0.20,0.65)	0.23<br>(-0.21,0.67)	0.19<br>(-0.37,0.74)	0.17<br>(-0.46,0.79)	-0.04<br>(-0.66,0.58)	Ins+La	-0.26<br>(-0.74,0.22)	0.09<br>(-0.42,0.60)
**0.49<br>(0.09,0.88)**	**0.49<br>(0.09,0.90)**	0.45<br>(-0.08,0.97)	0.43<br>(-0.17,1.02)	0.22<br>(-0.38,0.82)	0.26<br>(-0.22,0.74)	FO	0.35<br>(-0.13,0.83)
0.13<br>(-0.29,0.56)	0.14<br>(-0.30,0.58)	0.10<br>(-0.46,0.65)	0.08<br>(-0.54,0.70)	-0.13<br>(-0.75,0.49)	-0.09<br>(-0.60,0.42)	-0.35<br>(-0.83,0.13)	DF
0.08<br>(-0.35,0.51)	0.09<br>(-0.35,0.53)	0.04<br>(-0.51,0.59)	0.02<br>(-0.60,0.64)	-0.19<br>(-0.81,0.44)	-0.14<br>(-0.65,0.36)	-0.41<br>(-0.88,0.07)	-0.06<br>(-0.56,0.45)
0.05<br>(-0.19,0.28)	0.05<br>(-0.20,0.31)	0.01<br>(-0.41,0.43)	-0.01<br>(-0.52,0.49)	-0.22<br>(-0.73,0.29)	-0.18<br>(-0.54,0.18)	**-0.44<br>(-0.76,-0.12)**	-0.09<br>(-0.45,0.27)
-0.12<br>(-0.69,0.44)	-0.12<br>(-0.69,0.46)	-0.16<br>(-0.83,0.50)	-0.18<br>(-0.91,0.54)	-0.39<br>(-1.11,0.33)	-0.35<br>(-0.98,0.28)	**-0.61<br>(-1.22,-0.00)**	-0.26<br>(-0.89,0.37)

(continued)

Gestational Weight Gain*(Big Loop)(continued)*

-0.09 (-0.71,0.53)	-0.06 (-0.57,0.45)	0.11 (-0.61,0.84)
-0.17 (-0.72,0.39)	-0.13 (-0.56,0.29)	0.04 (-0.63,0.70)
-0.03 (-0.65,0.59)	-0.00 (-0.51,0.51)	0.17 (-0.55,0.89)
-0.05 (-0.48,0.38)	-0.01 (-0.25,0.22)	0.16 (-0.41,0.72)
-0.03 (-0.69,0.62)	-0.00 (-0.55,0.55)	0.17 (-0.58,0.92)
0.00 (-0.41,0.41)	0.04 (-0.16,0.23)	0.21 (-0.35,0.76)
-0.08 (-0.51,0.35)	-0.05 (-0.28,0.19)	0.12 (-0.44,0.69)
-0.09 (-0.53,0.35)	-0.05 (-0.31,0.20)	0.12 (-0.46,0.69)
-0.04 (-0.59,0.51)	-0.01 (-0.43,0.41)	0.16 (-0.50,0.83)
-0.02 (-0.64,0.60)	0.01 (-0.49,0.52)	0.18 (-0.54,0.91)
0.19 (-0.44,0.81)	0.22 (-0.29,0.73)	0.39 (-0.33,1.11)
0.14 (-0.36,0.65)	0.18 (-0.18,0.54)	0.35 (-0.28,0.98)
0.41 (-0.07,0.88)	**0.44 (0.12,0.76)**	**0.61 (0.00,1.22)**
0.06 (-0.45,0.56)	0.09 (-0.27,0.45)	0.26 (-0.37,0.89)
DASH	0.03 (-0.33,0.39)	0.20 (-0.42,0.83)
-0.03 (-0.39,0.33)	Control	0.17 (-0.34,0.69)
-0.20 (-0.83,0.42)	-0.17 (-0.69,0.34)	CHOICE

Supplementary materials

Cesarean Section Rate(Big Loop)

VD	1.10 (0.63,1.93)	**3.60 (1.44,9.02)**	0.73 (0.29,1.82)	0.58 (0.18,1.89)
0.91 (0.52,1.59)	RED	**3.27 (1.48,7.22)**	0.66 (0.30,1.46)	0.53 (0.18,1.56)
**0.28 (0.11,0.70)**	**0.31 (0.14,0.68)**	Pb	**0.20 (0.07,0.59)**	**0.16 (0.04,0.60)**
1.38 (0.55,3.46)	1.52 (0.68,3.36)	**4.95 (1.68,14.58)**	Omega3+VE	0.80 (0.22,2.96)
1.72 (0.53,5.62)	1.90 (0.64,5.63)	**6.21 (1.68,22.98)**	1.25 (0.34,4.64)	MgZnCaVD
0.81 (0.43,1.52)	0.89 (0.57,1.37)	**2.90 (1.24,6.78)**	0.58 (0.25,1.37)	0.47 (0.15,1.44)
1.25 (0.50,3.15)	1.38 (0.62,3.07)	**4.51 (1.53,13.30)**	0.91 (0.31,2.69)	0.73 (0.20,2.69)
0.43 (0.14,1.32)	0.47 (0.17,1.32)	1.53 (0.43,5.43)	0.31 (0.09,1.10)	0.25 (0.06,1.07)
1.17 (0.39,3.50)	1.29 (0.48,3.48)	**4.21 (1.23,14.44)**	0.85 (0.25,2.92)	0.68 (0.16,2.86)
0.93 (0.53,1.64)	1.03 (0.75,1.41)	**3.35 (1.51,7.44)**	0.68 (0.30,1.50)	0.54 (0.18,1.60)
1.33 (0.72,2.49)	1.47 (0.97,2.23)	**4.80 (2.07,11.12)**	0.97 (0.42,2.25)	0.77 (0.25,2.37)
0.77 (0.46,1.28)	0.85 (0.68,1.05)	**2.76 (1.29,5.91)**	0.56 (0.26,1.20)	0.44 (0.15,1.29)
0.70 (0.12,4.24)	0.77 (0.14,4.40)	2.52 (0.38,16.62)	0.51 (0.08,3.36)	0.41 (0.05,3.08)

(continued)

Supplementary materials

Cesarean Section Rate(Big Loop)(continued)

1.24 (0.66,2.35)	0.80 (0.32,2.01)	2.35 (0.76,7.29)	0.86 (0.29,2.56)	1.07 (0.61,1.89)	0.75 (0.40,1.40)	1.30 (0.78,2.18)	1.43 (0.24,8.67)
1.13 (0.73,1.74)	0.72 (0.33,1.61)	2.13 (0.76,5.99)	0.78 (0.29,2.09)	0.97 (0.71,1.34)	0.68 (0.45,1.03)	1.18 (0.95,1.48)	1.30 (0.23,7.40)
**0.35 (0.15,0.81)**	**0.22 (0.08,0.66)**	0.65 (0.18,2.31)	**0.24 (0.07,0.81)**	**0.30 (0.13,0.66)**	**0.21 (0.09,0.48)**	**0.36 (0.17,0.78)**	0.40 (0.06,2.63)
1.71 (0.73,4.01)	1.10 (0.37,3.25)	3.23 (0.91,11.47)	1.18 (0.34,4.04)	1.48 (0.67,3.28)	1.03 (0.44,2.39)	1.80 (0.84,3.85)	1.97 (0.30,13.02)
2.14 (0.69,6.62)	1.38 (0.37,5.12)	4.05 (0.93,17.56)	1.47 (0.35,6.22)	1.85 (0.62,5.50)	1.29 (0.42,3.96)	2.25 (0.78,6.52)	2.47 (0.32,18.76)
MNP	0.64 (0.27,1.51)	1.89 (0.64,5.56)	0.69 (0.24,1.95)	0.86 (0.56,1.35)	0.60 (0.36,1.01)	1.05 (0.72,1.53)	1.15 (0.20,6.75)
1.55 (0.66,3.66)	MLC	2.94 (0.83,10.45)	1.07 (0.31,3.68)	1.34 (0.60,3.00)	0.94 (0.40,2.18)	1.63 (0.76,3.52)	1.79 (0.27,11.86)
0.53 (0.18,1.56)	0.34 (0.10,1.21)	LGD	0.36 (0.09,1.48)	0.46 (0.16,1.29)	**0.32 (0.11,0.93)**	0.56 (0.20,1.53)	0.61 (0.08,4.51)
1.45 (0.51,4.11)	0.93 (0.27,3.22)	2.75 (0.68,11.13)	Ins+La	1.26 (0.46,3.40)	0.88 (0.31,2.46)	1.53 (0.58,4.02)	1.67 (0.23,12.12)
1.16 (0.74,1.80)	0.74 (0.33,1.66)	2.19 (0.78,6.16)	0.80 (0.29,2.16)	FO	0.70 (0.46,1.06)	1.21 (0.96,1.53)	1.33 (0.23,7.61)
1.66 (0.99,2.78)	1.07 (0.46,2.48)	**3.13 (1.07,9.13)**	1.14 (0.41,3.20)	1.43 (0.94,2.19)	DASH	**1.74 (1.22,2.48)**	1.91 (0.33,11.13)
0.95 (0.65,1.39)	0.61 (0.28,1.32)	1.80 (0.66,4.94)	0.66 (0.25,1.73)	0.82 (0.65,1.04)	**0.57 (0.40,0.82)**	Control	1.10 (0.19,6.17)
0.87 (0.15,5.09)	0.56 (0.08,3.70)	1.64 (0.22,12.14)	0.60 (0.08,4.33)	0.75 (0.13,4.29)	0.52 (0.09,3.05)	0.91 (0.16,5.13)	CHOICE

The red numbers represent a significant difference, and the yellow tables indicate a significant change compared to the original results

Supplementary materials

*Table S6:*GRADE Assessment

High-Risk Populations of GDM

	Certainty assessment							Certainty	Importance
Outcomes	№ of studies	Study design	Risk of bias	Inconsistency	Indirectness	Imprecision	Other considerations		
Incidence of GDM	20	randomised trials	serious[a]	not serious	not serious	not serious	none	⊕⊕⊕◯ Moderate[a]	CRITICAL
Fasting blood glucose	11	randomised trials	serious[a]	not serious	not serious	not serious	none	⊕⊕⊕◯ Moderate[a]	CRITICAL
Gestational weight gain	11	randomised trials	serious[a]	not serious	not serious	not serious	none	⊕⊕⊕◯ Moderate[a]	CRITICAL
Cesarean section rate	16	randomised trials	serious[a]	not serious	not serious	not serious	none	⊕⊕⊕◯ Moderate[a]	CRITICAL

Patients with GDM

	Certainty assessment							Certainty	Importance
Outcomes	№ of studies	Study design	Risk of bias	Inconsistency	Indirectness	Imprecision	Other considerations		
Fasting blood glucose	29	randomised trials	serious[a]	not serious	not serious	not serious	none	⊕⊕⊕◯ Moderate[a]	CRITICAL
HOMA-IR	19	randomised trials	serious[a]	not serious	not serious	not serious	none	⊕⊕⊕◯ Moderate[a]	CRITICAL
Glycated hemoglobin	11	randomised trials	serious[a]	not serious	not serious	not serious	none	⊕⊕⊕◯ Moderate[a]	CRITICAL
Gestational weight gain	24	randomised trials	serious[a]	not serious	not serious	not serious	none	⊕⊕⊕◯ Moderate[a]	CRITICAL
Cesarean section rate	14	randomised trials	serious[a]	not serious	not serious	not serious	none	⊕⊕⊕◯ Moderate[a]	CRITICAL

Explanations
a. The risk of bias was assessed as “some concerns” using the ROB 2 tool.

Supplementary materials

Figure S1: Cumulative Rank Curve

High-Risk Populations of GDM

Incidence of GDM

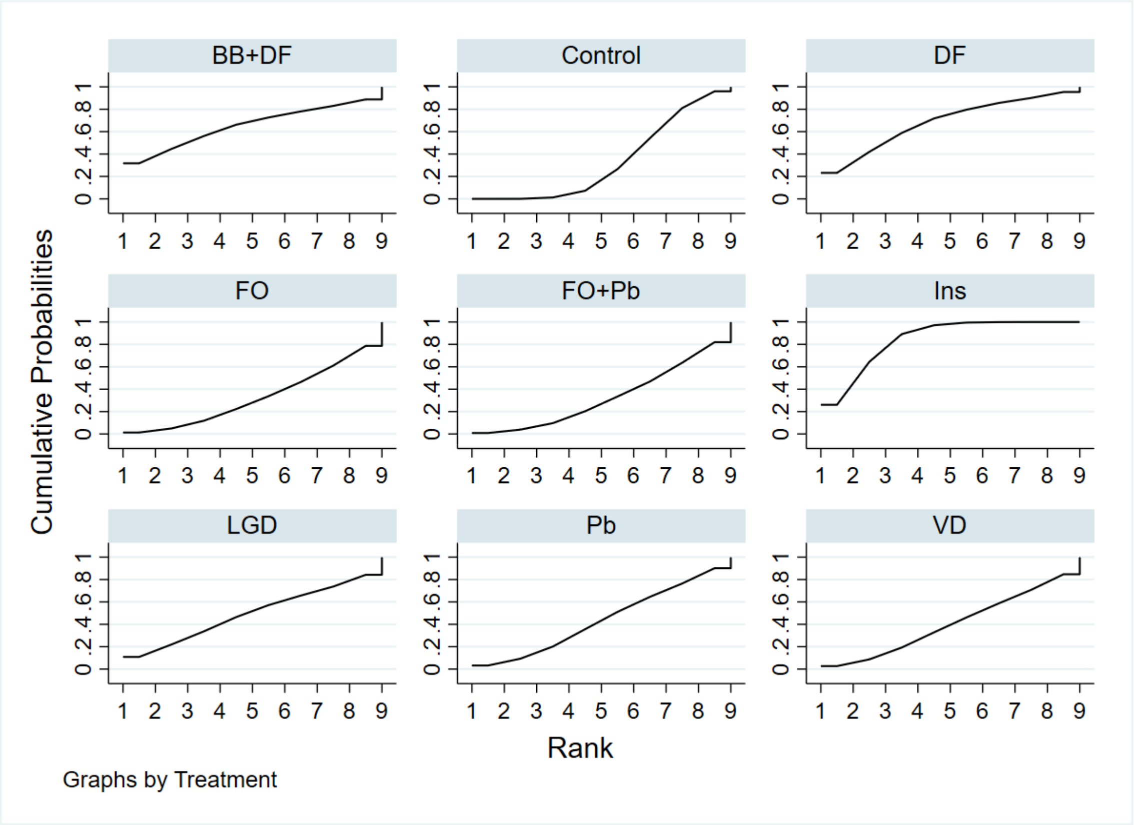

Supplementary materials

Fasting Blood Glucose

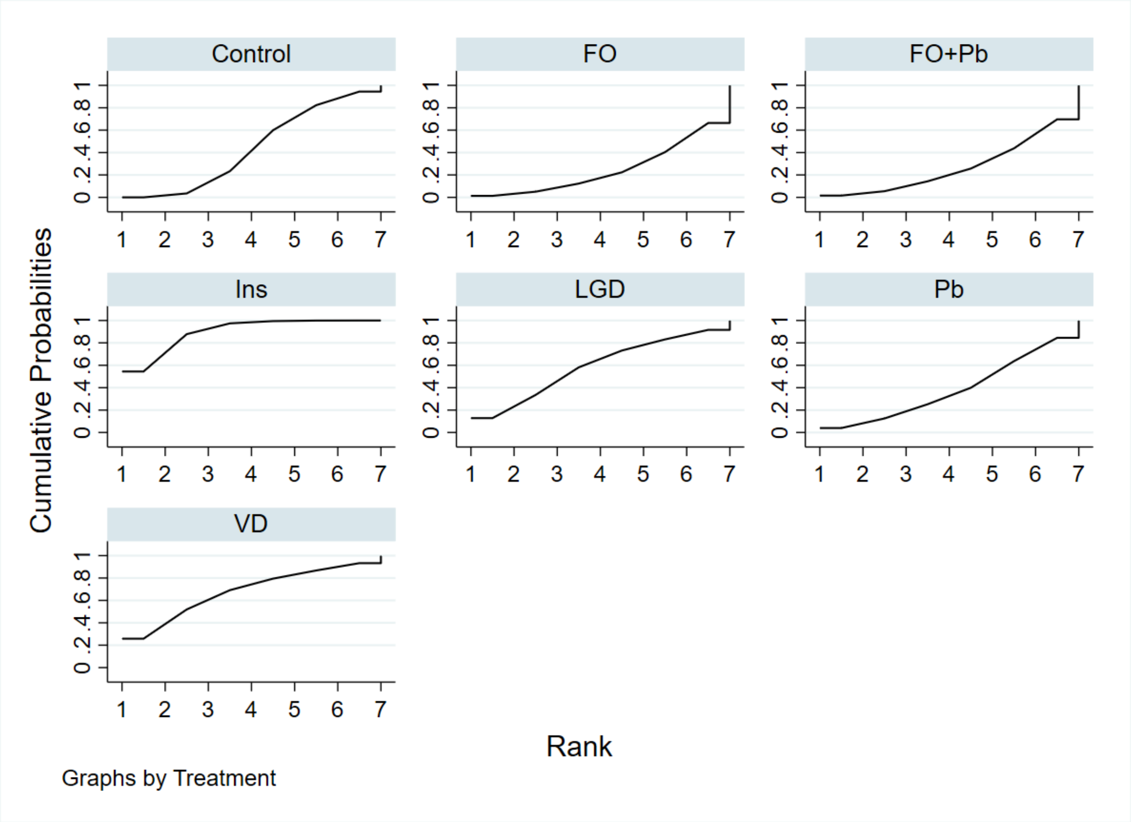

Supplementary materials

Gestational Weight Gain

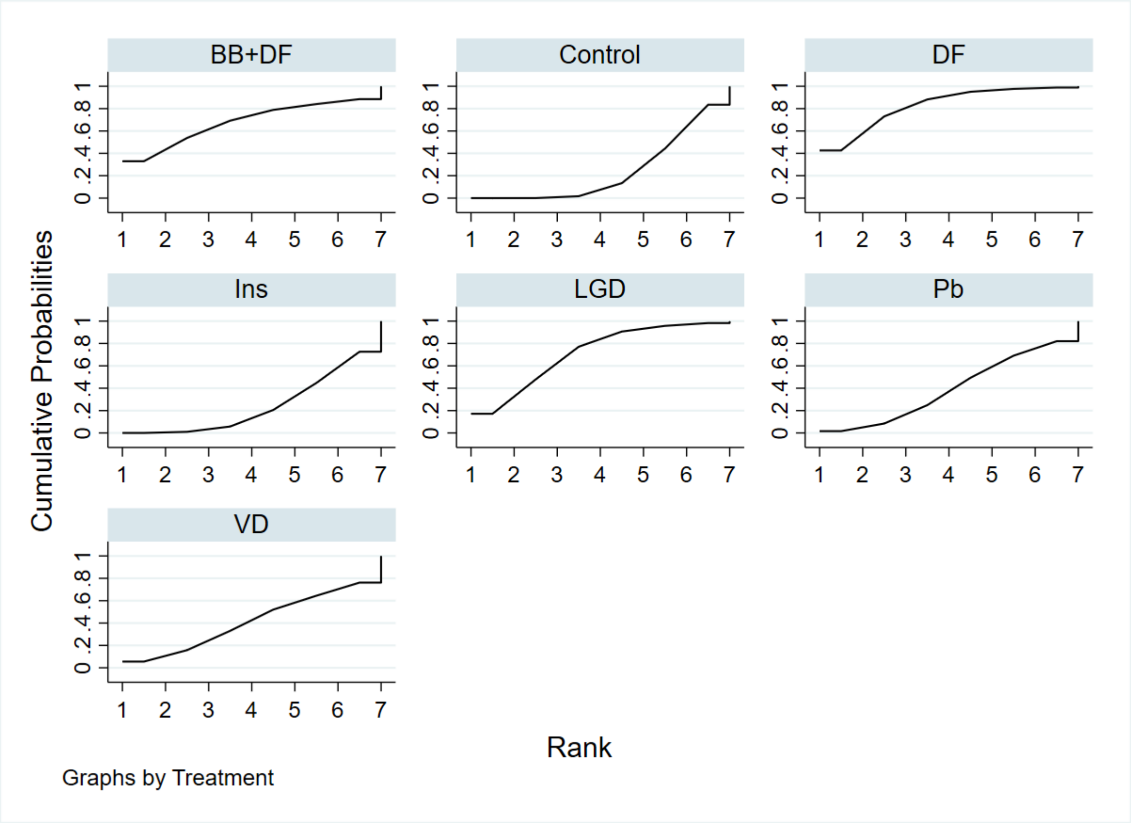

Supplementary materials

Cesarean Section Rate

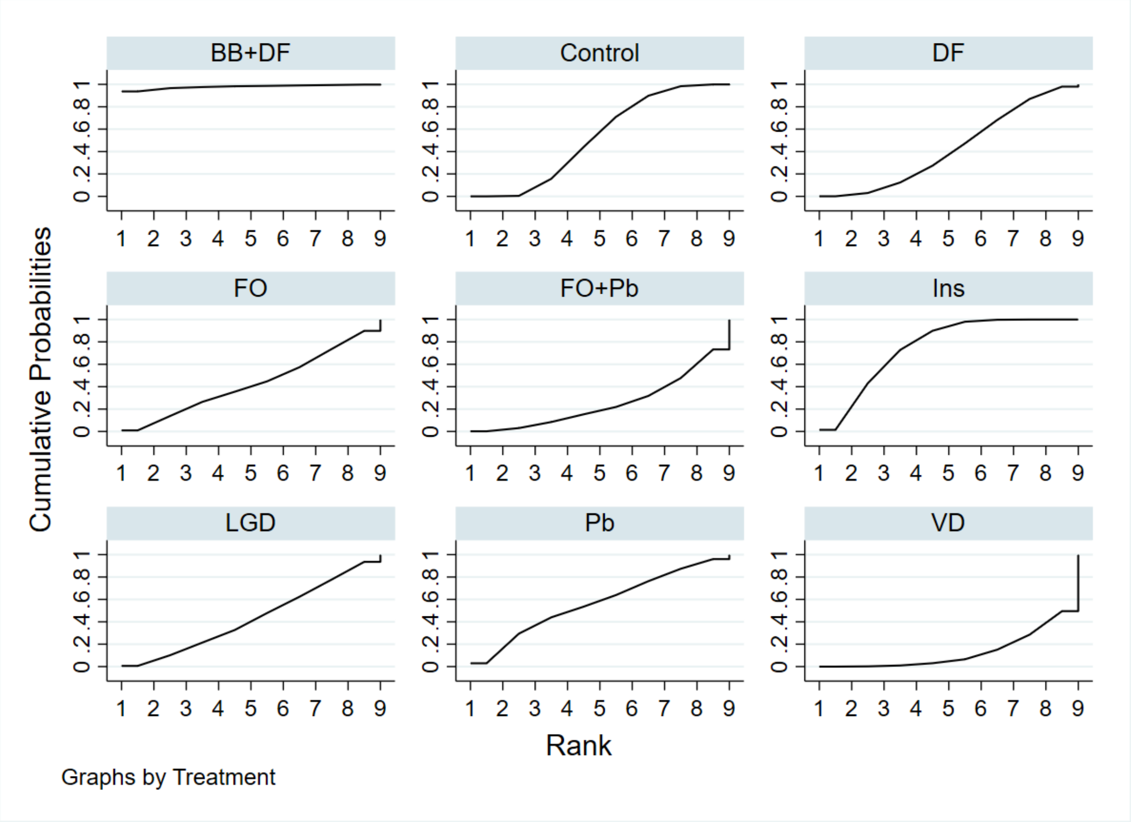

Supplementary materials

Patients with GDM

Fasting Blood Glucose

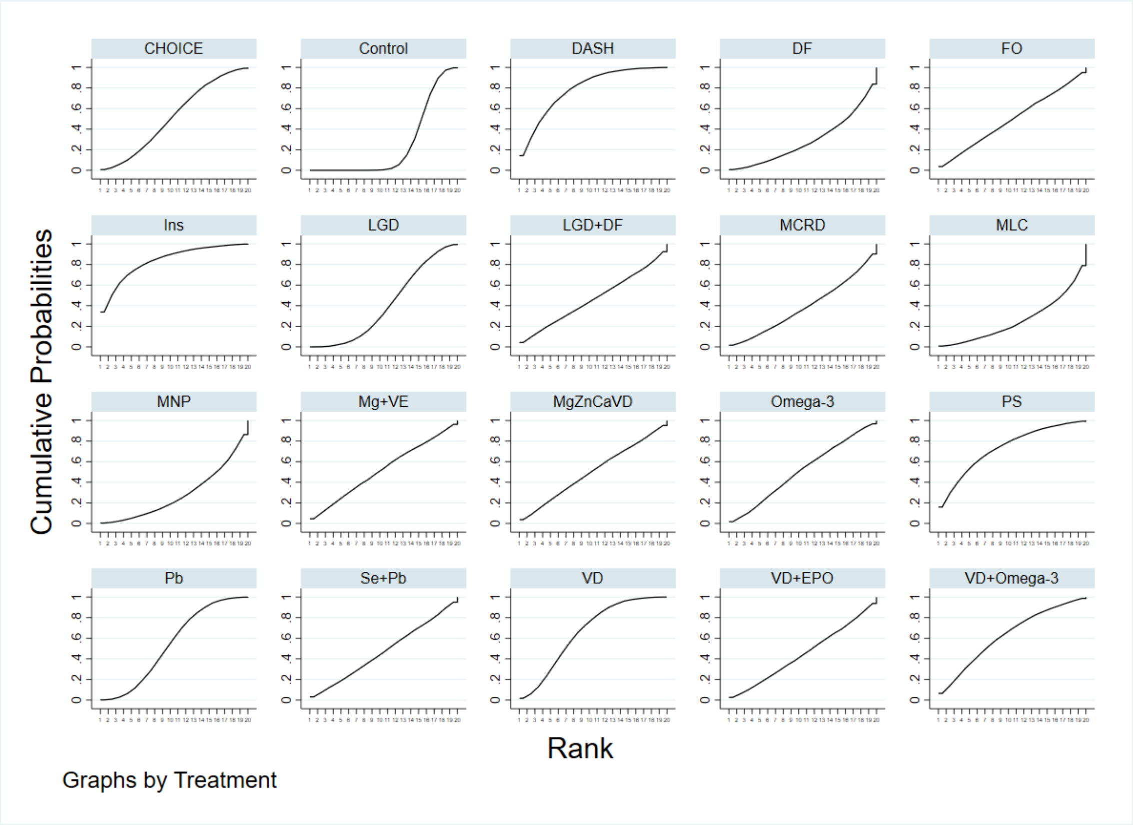

Supplementary materials

HOMA-IR

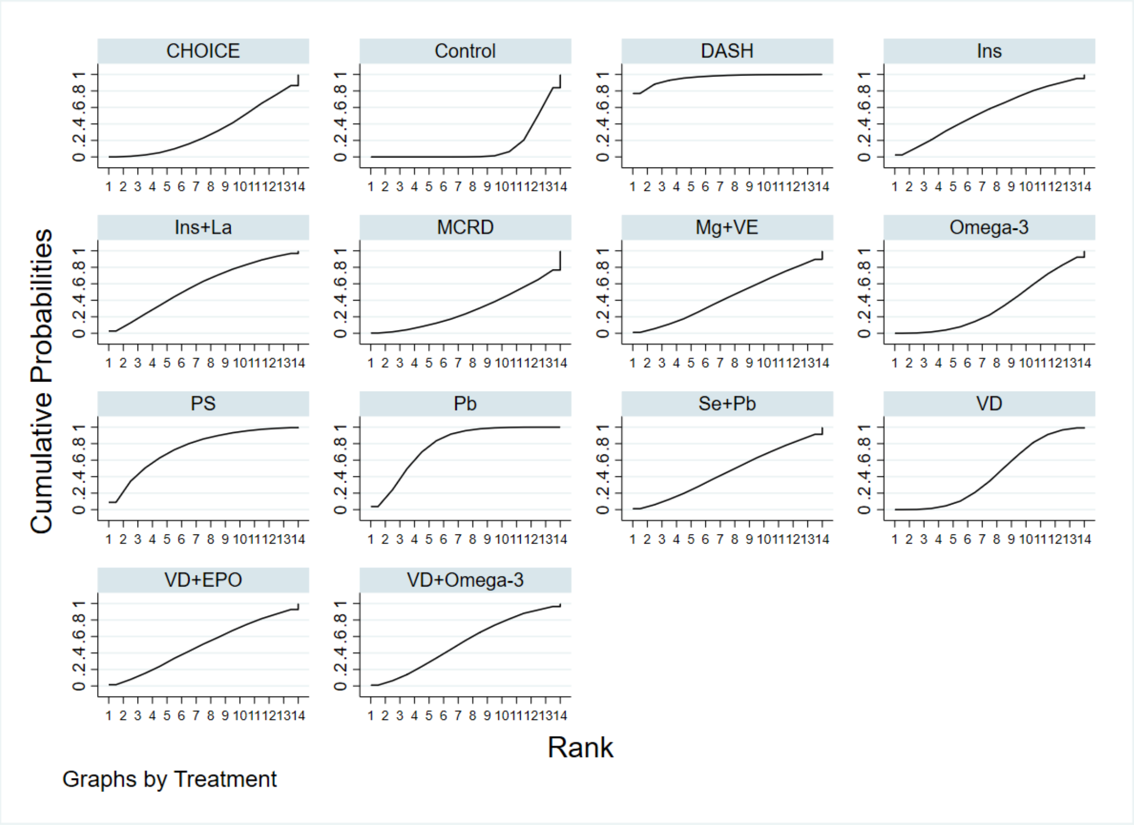

Supplementary materials

Glycated Hemoglobin

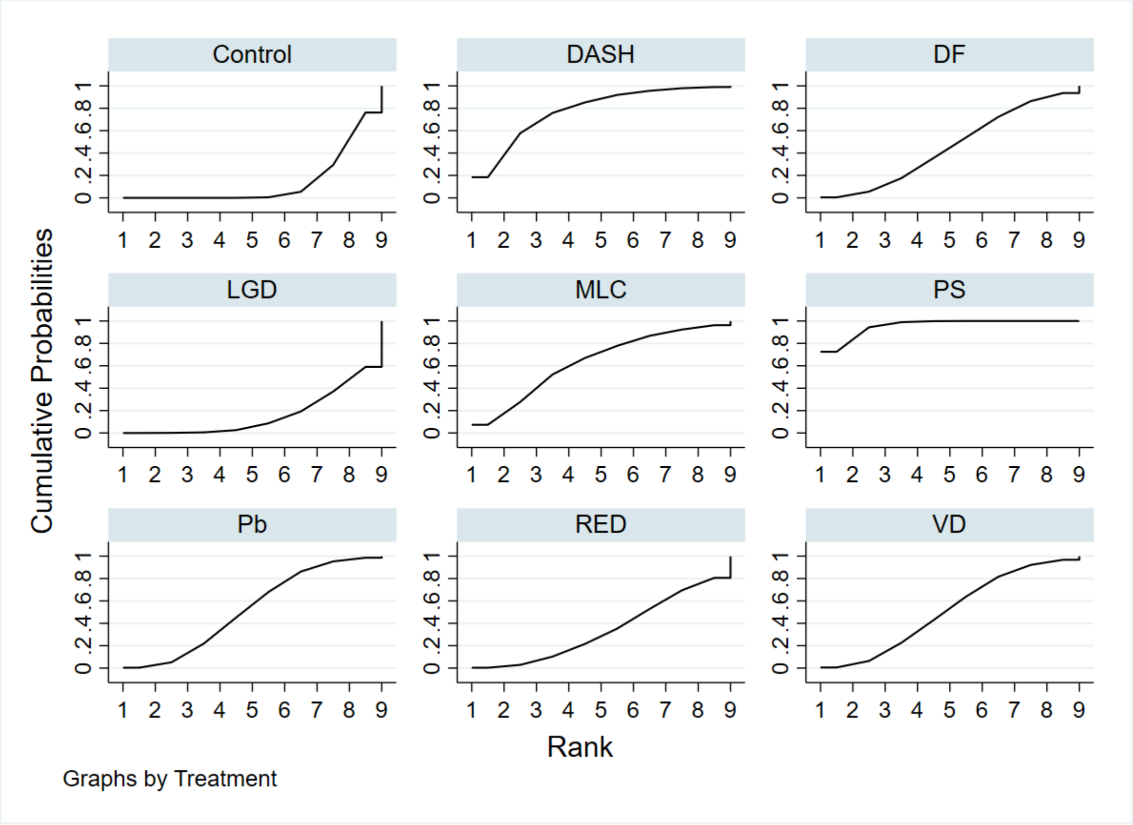

Supplementary materials

Gestational Weight Gain

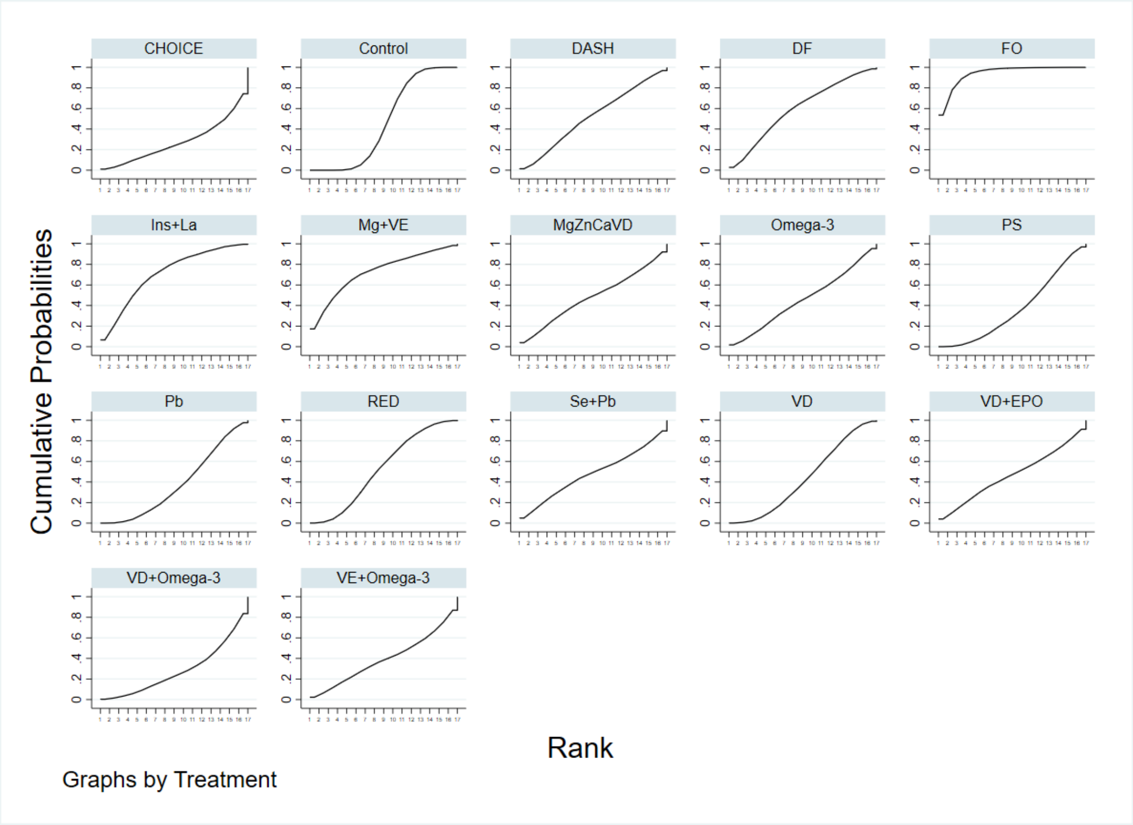

Supplementary materials

Cesarean Section Rate

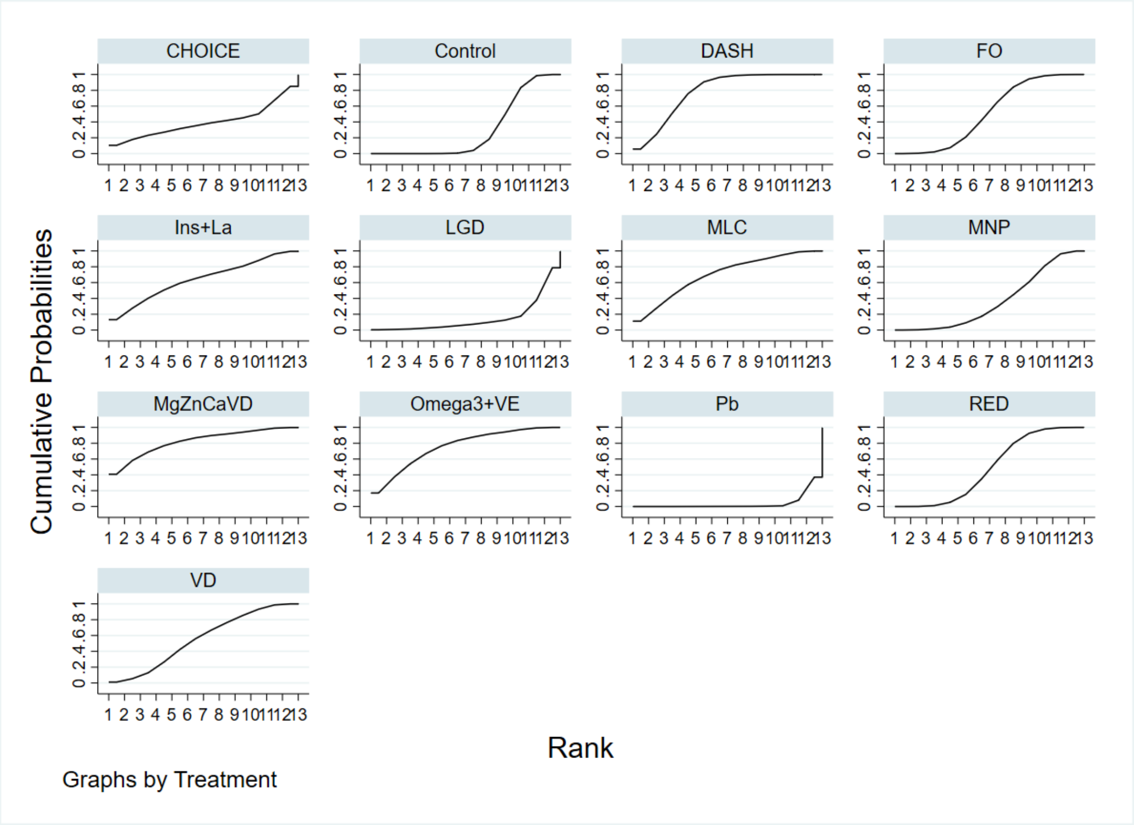

Supplementary materials

Figure S2: Inconsistency analysis

RED:Restricted Energy Diet; CHOICE:60% Carbohydrate,25% Fat,15% Protein; MCRD:Carbohydrate-Restricted Diet; DF:Dietary Fiber; MLC:Modestly lower Carbohydrate Diet; LGD:Low Glycemic Index Diet; MNP:Macronutrient Preload; DASH:Dietary Approaches to Stop Hypertension;BB: Blueberry;FO:Fish Oil;Pb:Probiotics;La:Alpha-Lactalbumin

High-Risk Populations of GDM

Incidence of GDM

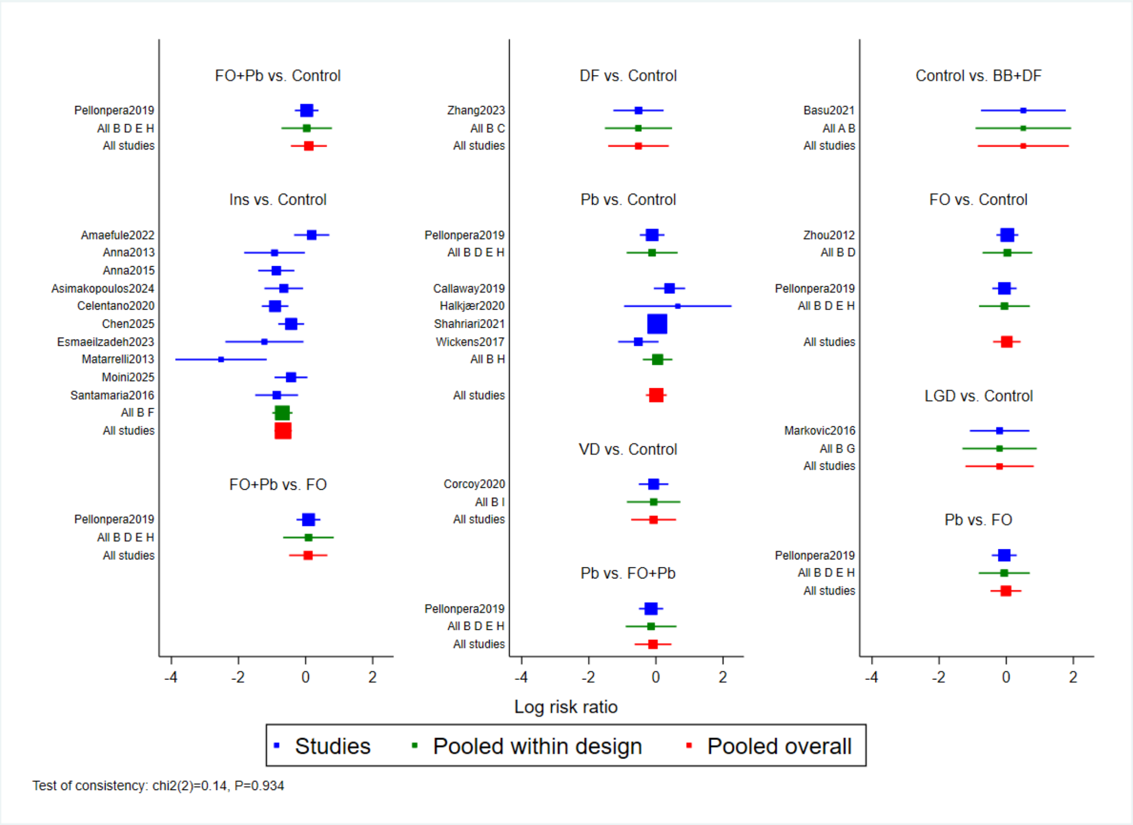

Supplementary materials

Fasting Blood Glucose

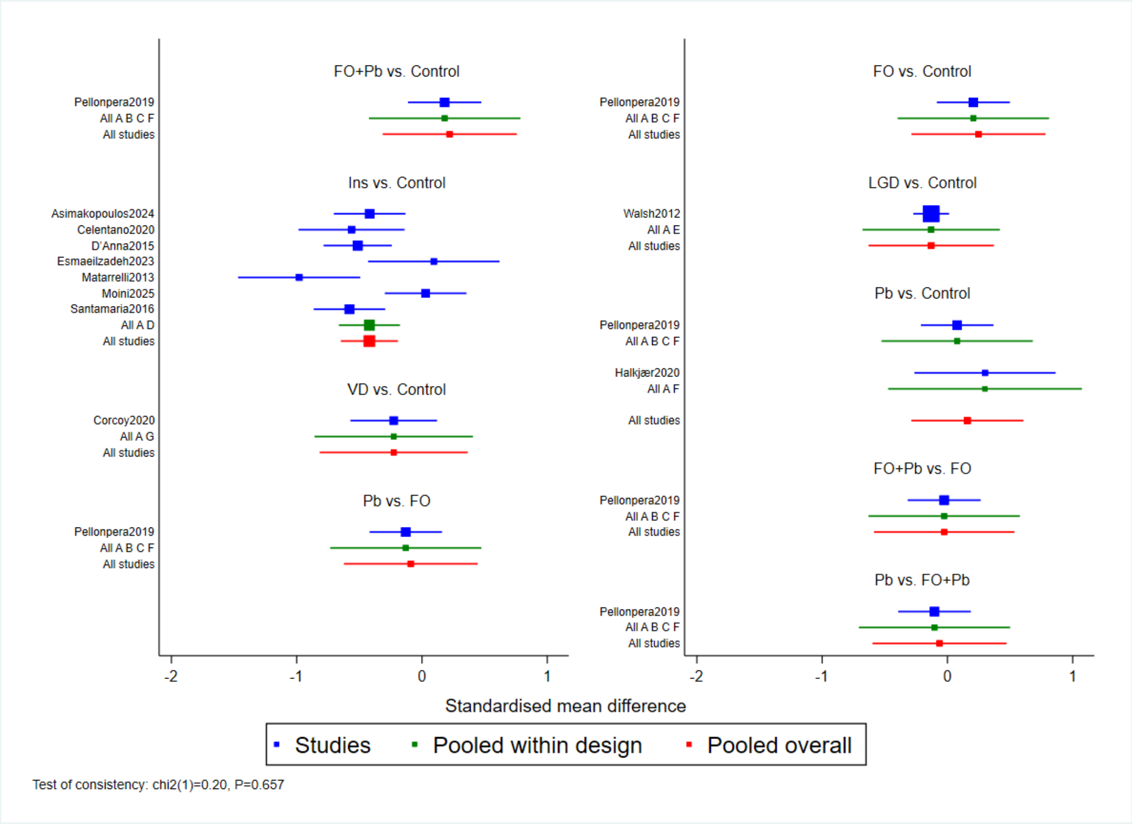

Supplementary materials

Gestational Weight Gain

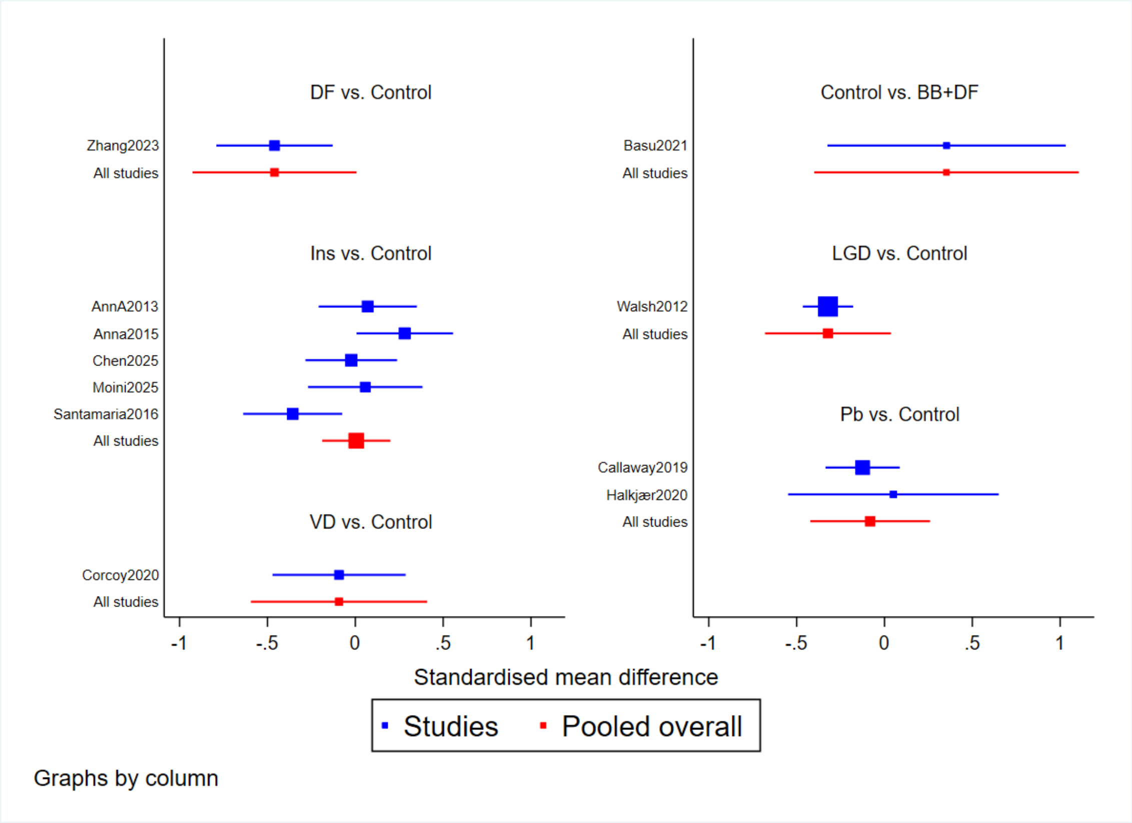

Supplementary materials

Cesarean Section Rate

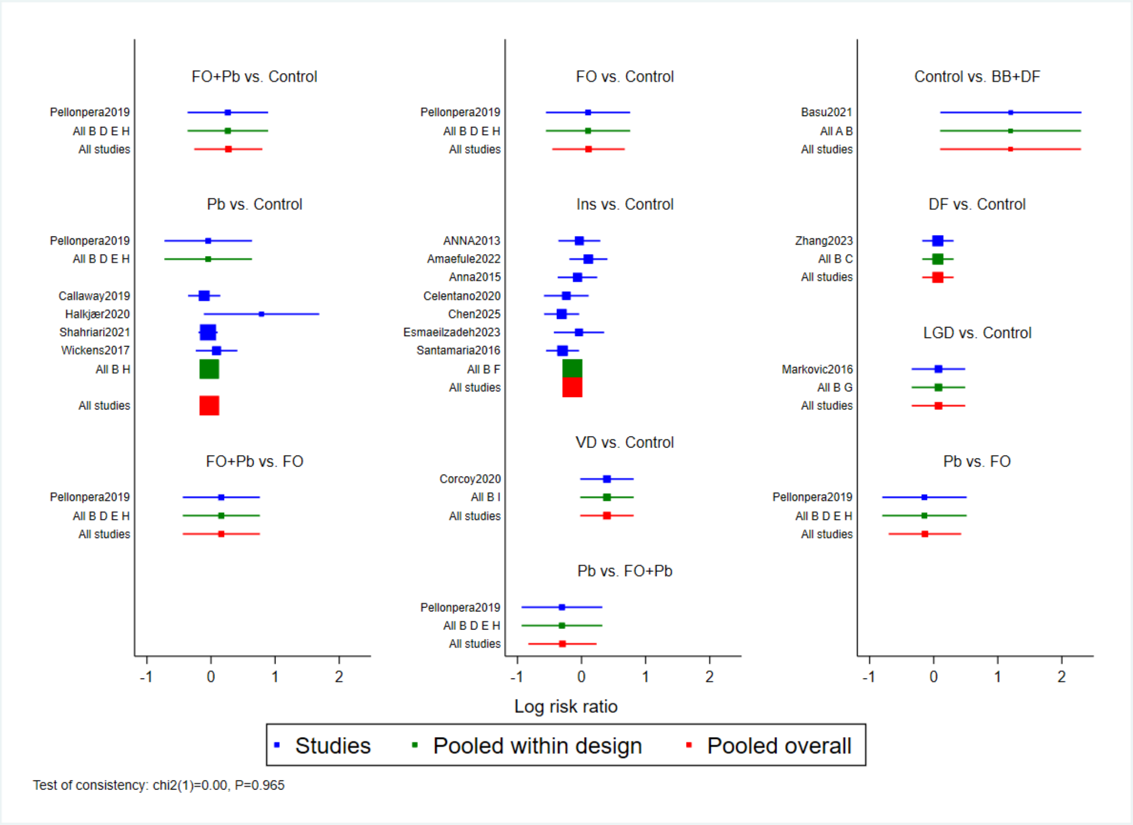

Supplementary materials

Patients with GDM

Fasting Blood Glucose

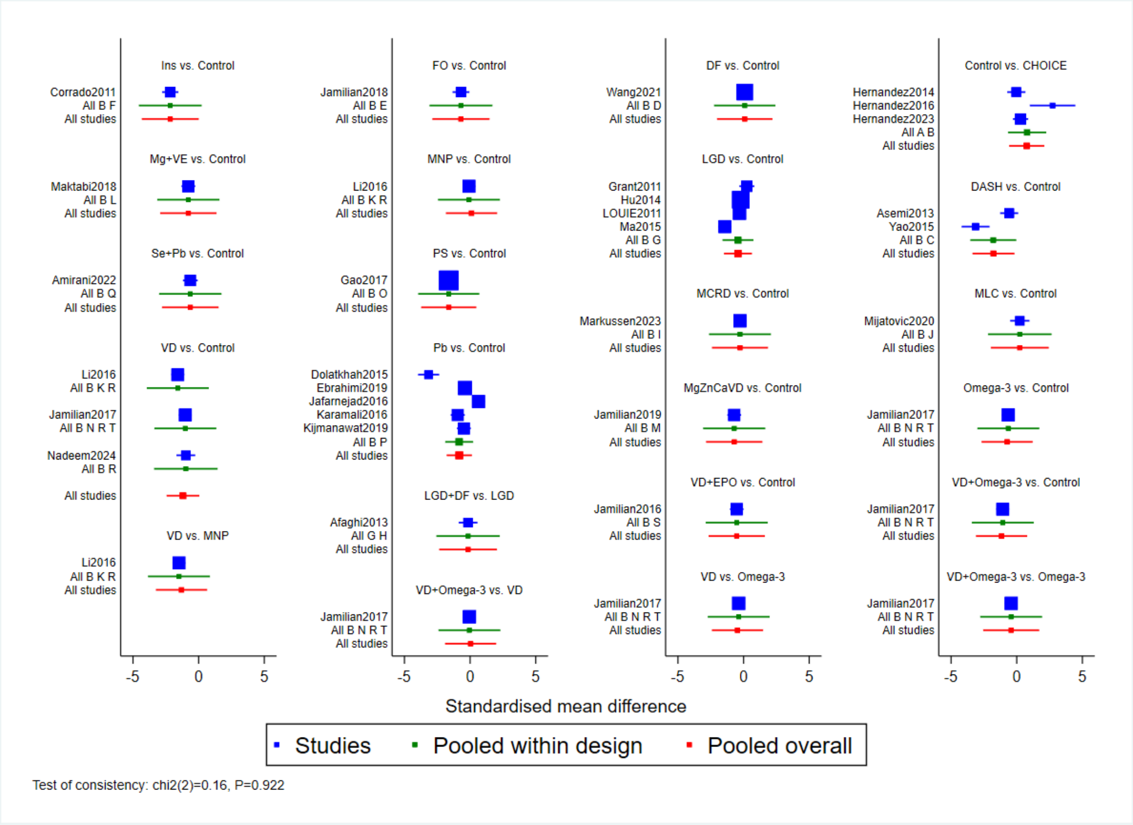

Supplementary materials

HOMA-IR

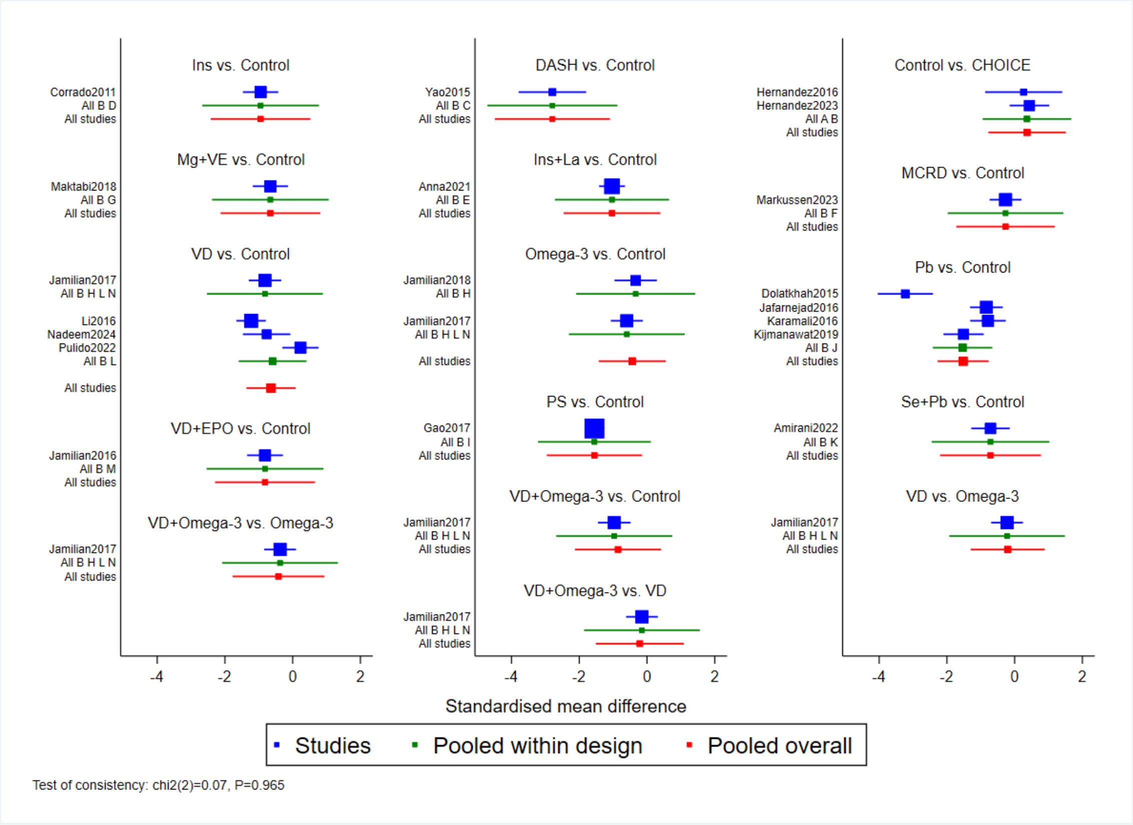

Supplementary materials

Glycated Hemoglobin

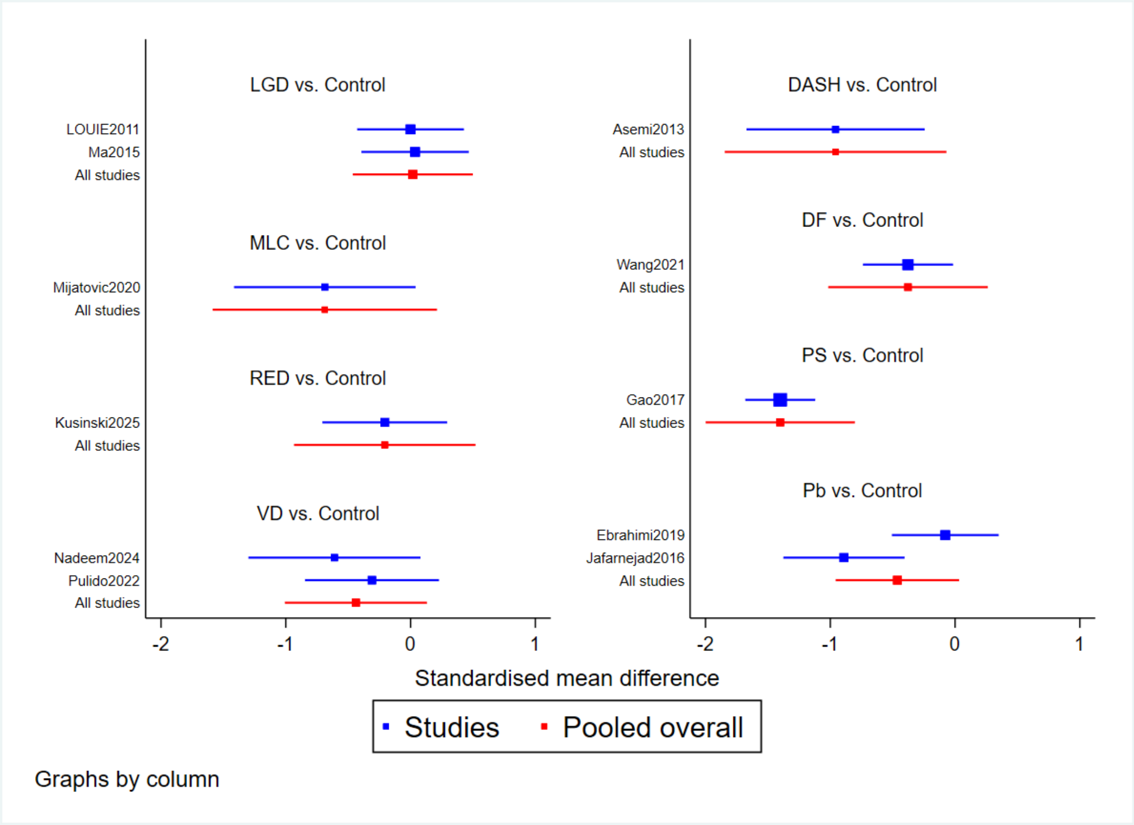

Supplementary materials

Gestational Weight Gain

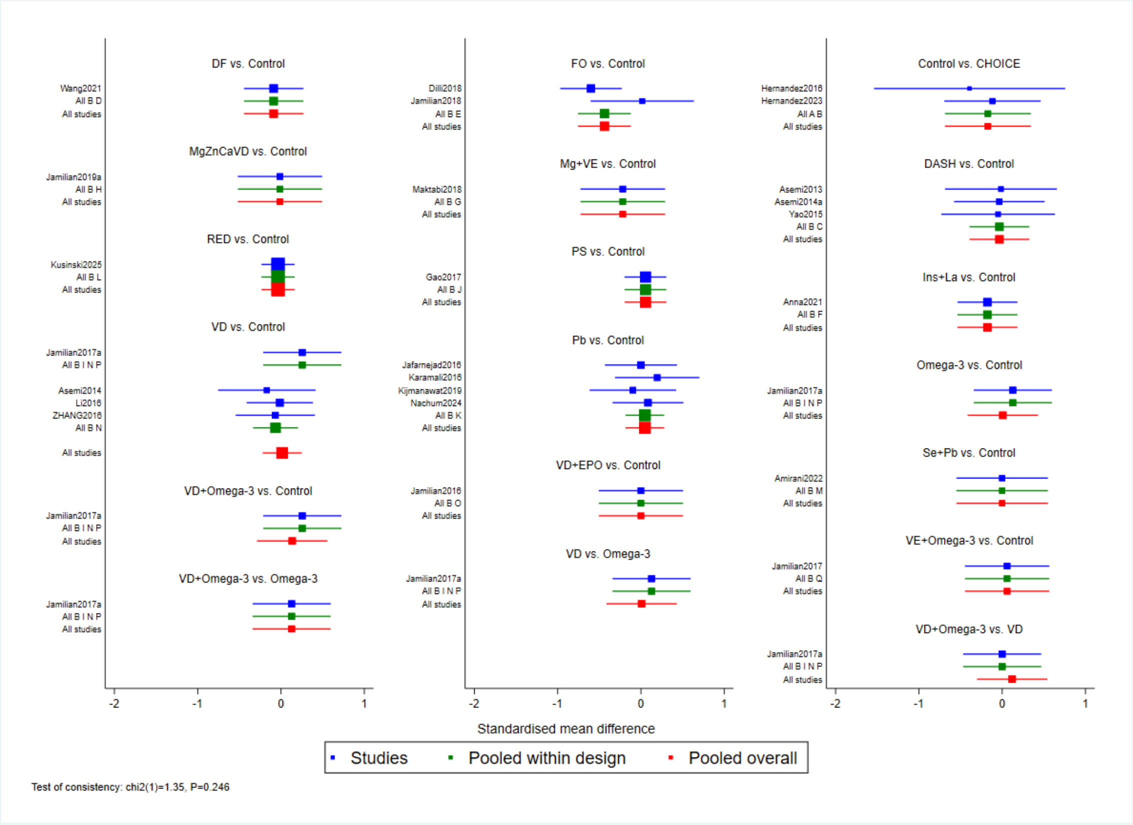

Supplementary materials

Cesarean Section Rate

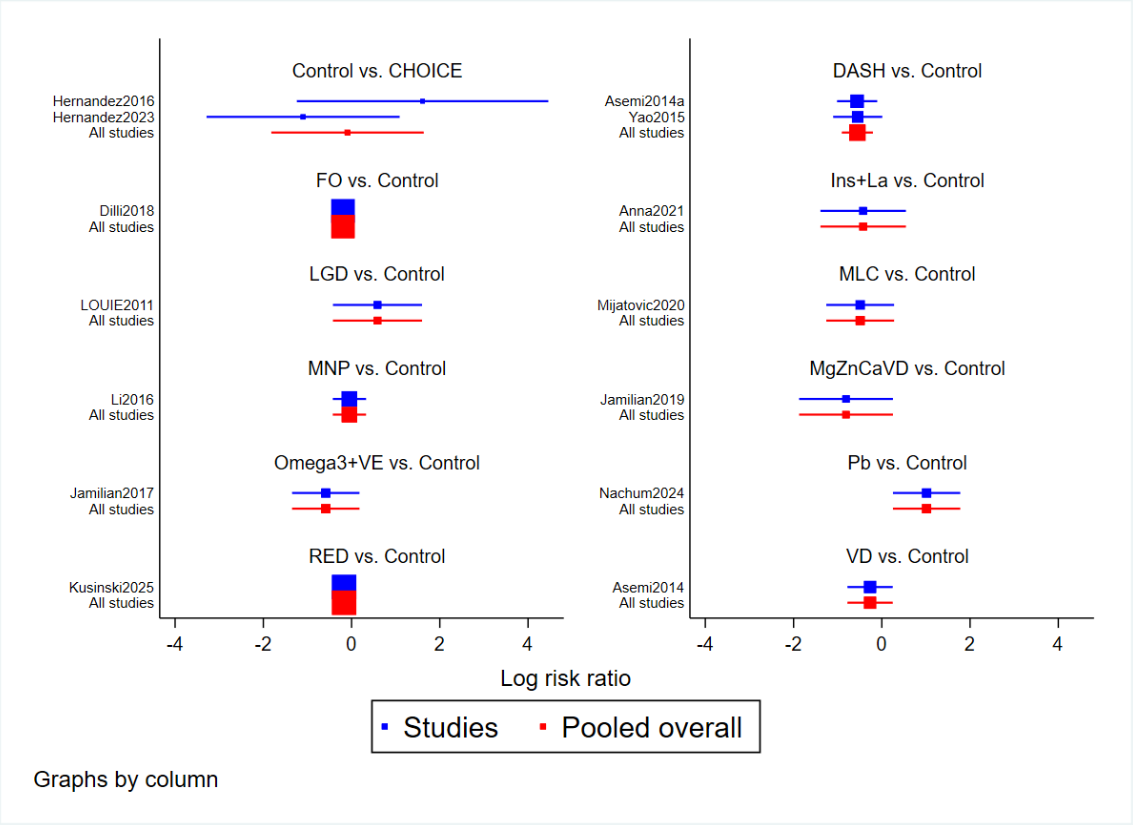

Supplementary materials

Figure S3:Egger's publication bias plot

High-Risk Populations of GDM

Incidence of GDM

.
```
Number of studies =   25                                   Root MSE      =    1.543
```

Std_Eff	Coef.	Std. Err.	t	P>|t|	[95% Conf.	Interval]
slope	.2066537	.1687873	1.22	0.233	-.1425094	.5558168
bias	-1.821544	.7458338	-2.44	0.023	-3.364419	-.2786696

```
Test of H0: no small-study effects          P = 0.023
```

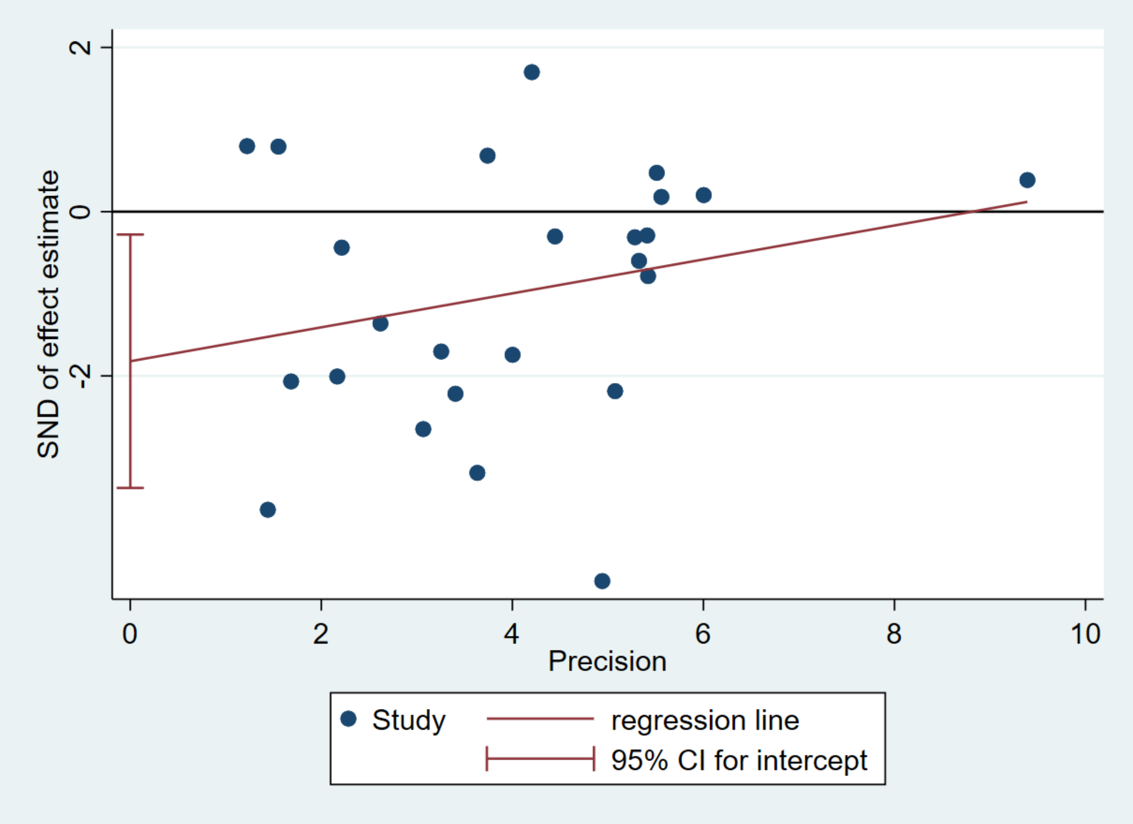

Supplementary materials

Fasting Blood Glucose

```
.
Number of studies =   16                            Root MSE      =   1.923
------------------------------------------------------------------------------
     Std_Eff |      Coef.   Std. Err.       t    P>|t|     [95% Conf. Interval]
-------------+----------------------------------------------------------------
       slope |  -.0950302   .2136661    -0.44   0.663    -.5532985     .363238
        bias |   -.463631   1.452889    -0.32   0.754    -3.579768    2.652506
------------------------------------------------------------------------------

Test of H0: no small-study effects          P = 0.754
```

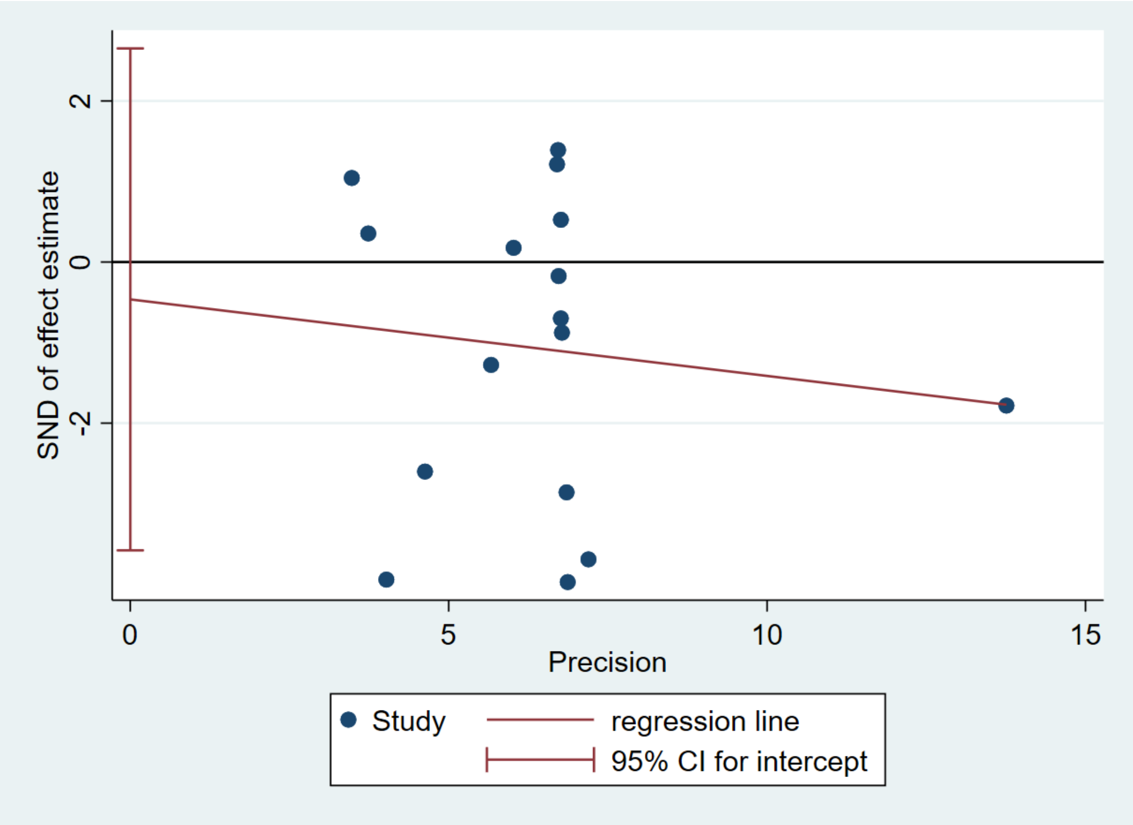

Supplementary materials

Gestational Weight Gain

.
```
Number of studies =   11                                 Root MSE      =    1.534
```

Std_Eff	Coef.	Std. Err.	t	P>|t|	[95% Conf.	Interval]
slope	-.4012079	.1654419	-2.43	0.038	-.7754636	-.0269523
bias	2.060918	1.217103	1.69	0.125	-.6923601	4.814197

```
Test of H0: no small-study effects          P = 0.125
```

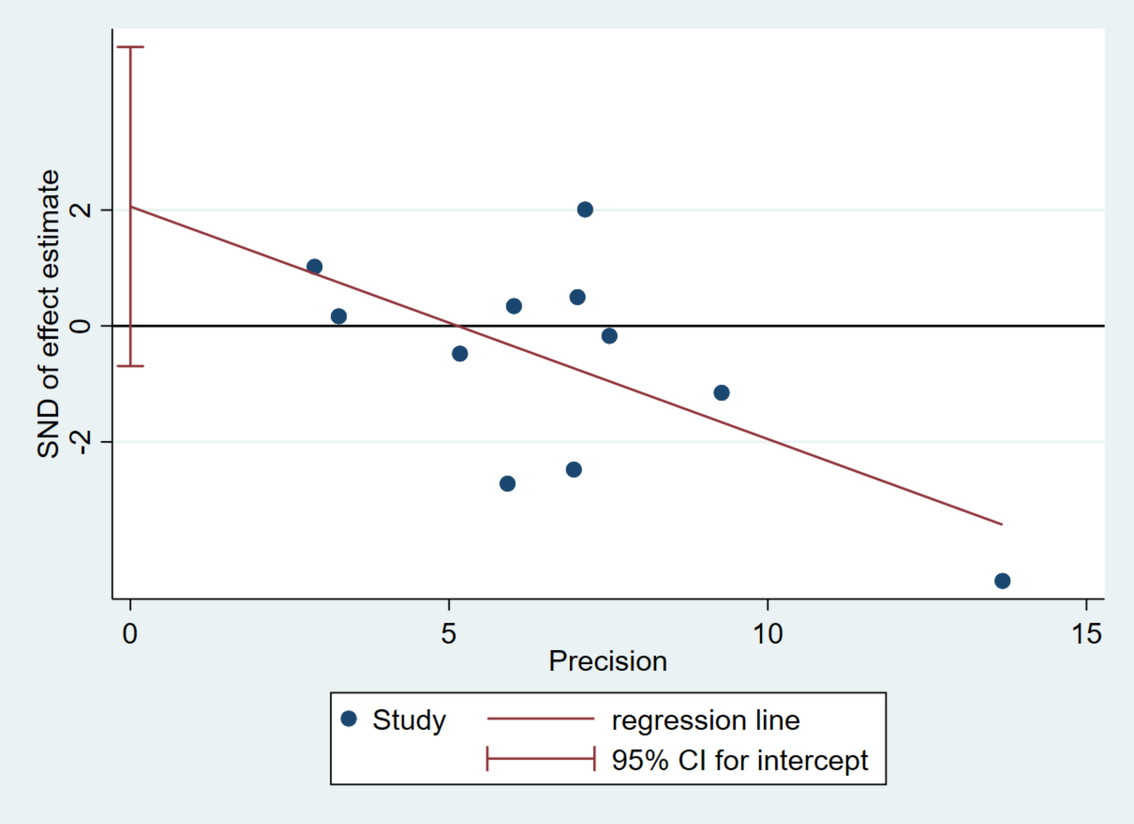

Supplementary materials

Cesarean Section Rate

.
Number of studies = 21   Root MSE = 1.065

Std_Eff	Coef.	Std. Err.	t	P>|t|	[95% Conf.	Interval]
slope	-.2059306	.0902632	-2.28	0.034	-.3948536	-.0170076
bias	1.083845	.5303441	2.04	0.055	-.0261783	2.193868

Test of H0: no small-study effects   P = 0.055

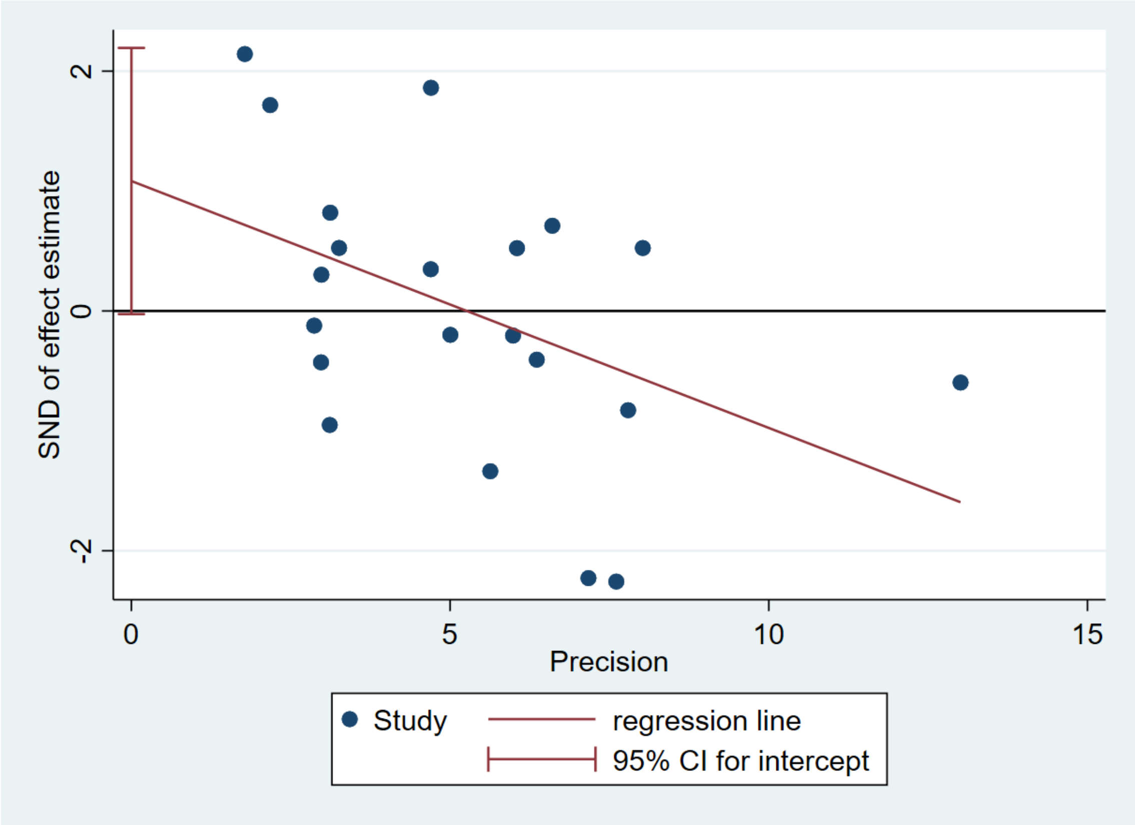

Supplementary materials

Patients with GDM

Fasting Blood Glucose

.
Number of studies = 35   Root MSE = 2.929

Std_Eff	Coef.	Std. Err.	t	P>|t|	[95% Conf.	Interval]
slope	-.6854417	.4803247	-1.43	0.163	-1.66267	.2917863
bias	.1805252	1.856489	0.10	0.923	-3.596529	3.95758

Test of H0: no small-study effects   P = 0.923

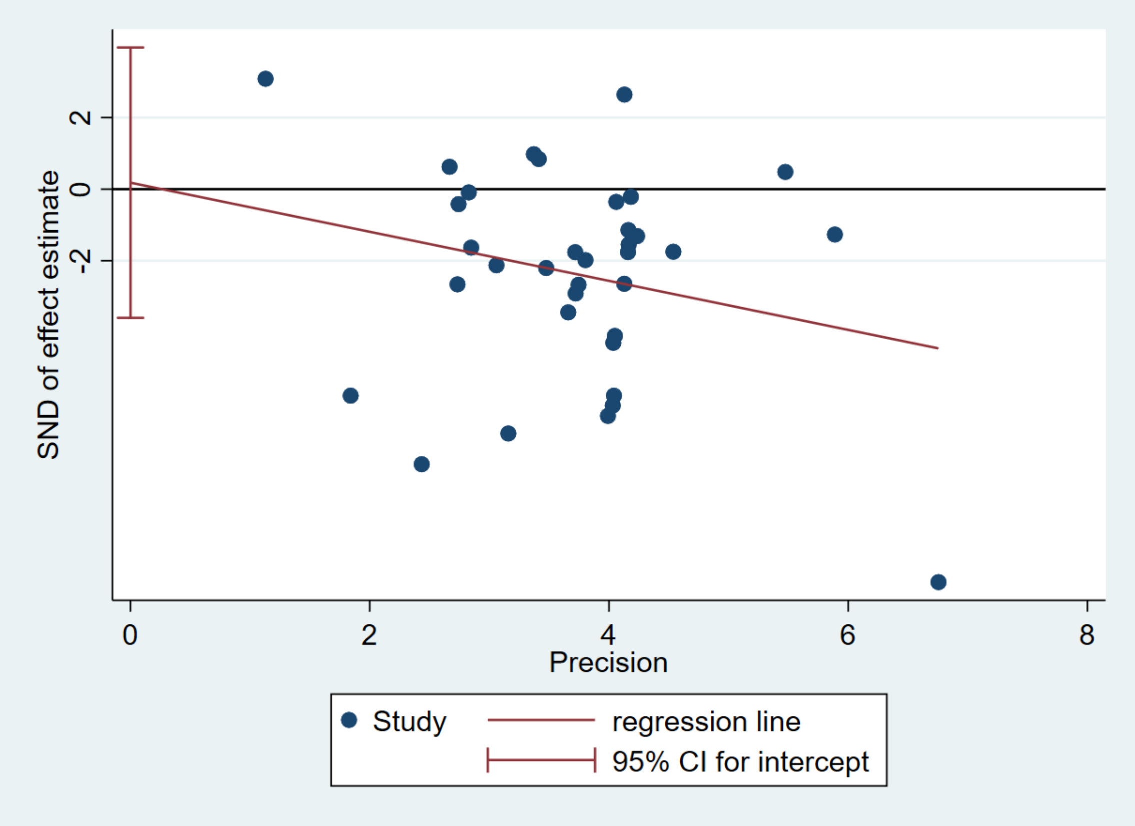

Supplementary materials

HOMA-IR

.

Number of studies =   24   Root MSE   =   2.567

Std_Eff	Coef.	Std. Err.	t	P>|t|	[95% Conf.	Interval]
slope	-1.046898	.5247894	-1.99	0.059	-2.135244	.0414485
bias	.9402117	2.040761	0.46	0.650	-3.292067	5.172491

Test of H0: no small-study effects   P = 0.650

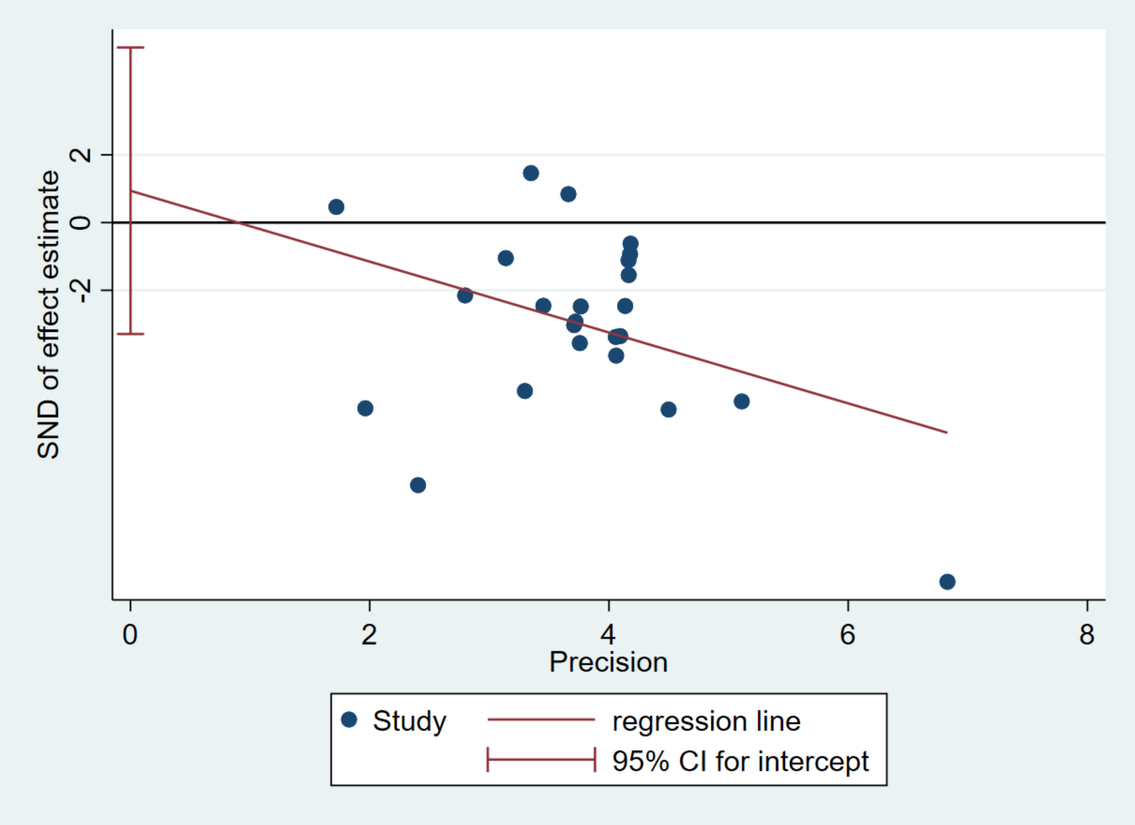

Supplementary materials

Glycated Hemoglobin

.
Number of studies = 11   Root MSE = 2.422

Std_Eff	Coef.	Std. Err.	t	P>|t|	[95% Conf.	Interval]
slope	-1.216774	.5992487	-2.03	0.073	-2.572369	.138821
bias	2.92911	2.610985	1.12	0.291	-2.977348	8.835568

Test of H0: no small-study effects   P = 0.291

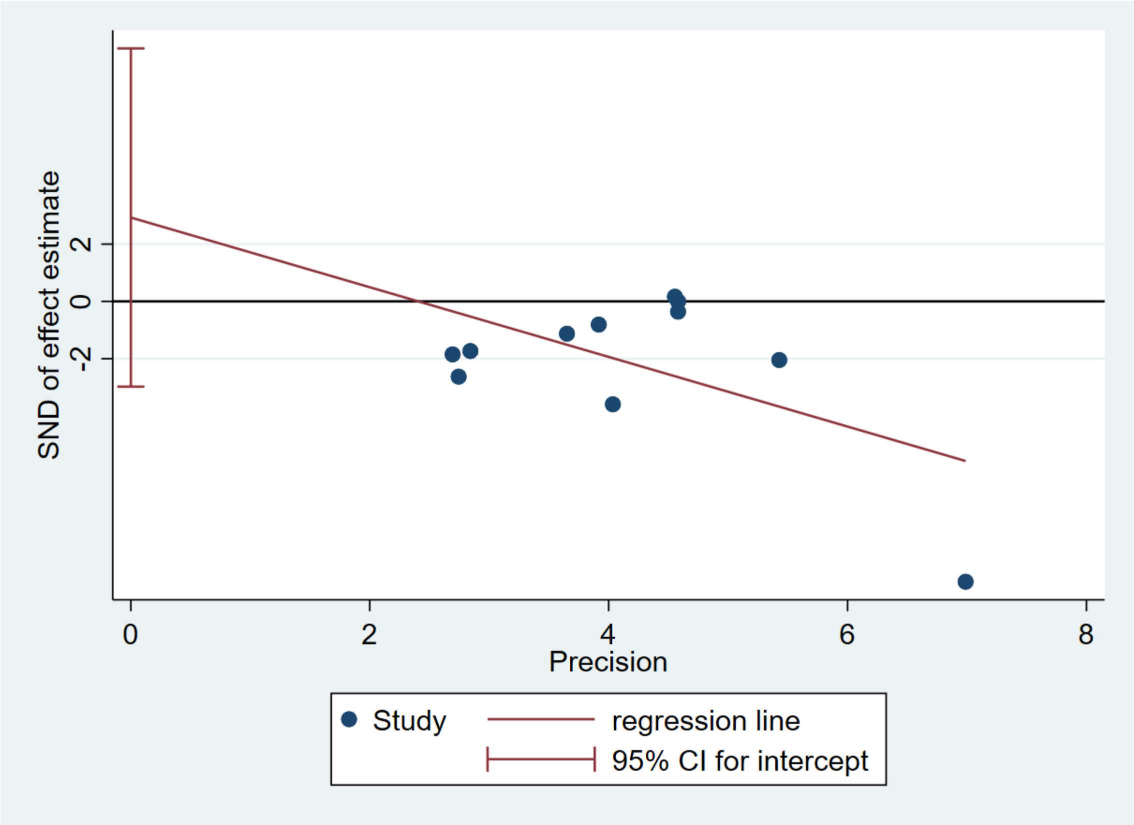

Supplementary materials

Gestational Weight Gain

Number of studies = 29 Root MSE = .7933

Std_Eff	Coef.	Std. Err.	t	P>|t|	[95% Conf.	Interval]
slope	-.0407005	.0996585	-0.41	0.686	-.2451827	.1637818
bias	.0735049	.4535099	0.16	0.872	-.8570206	1.00403

Test of H0: no small-study effects P = 0.872

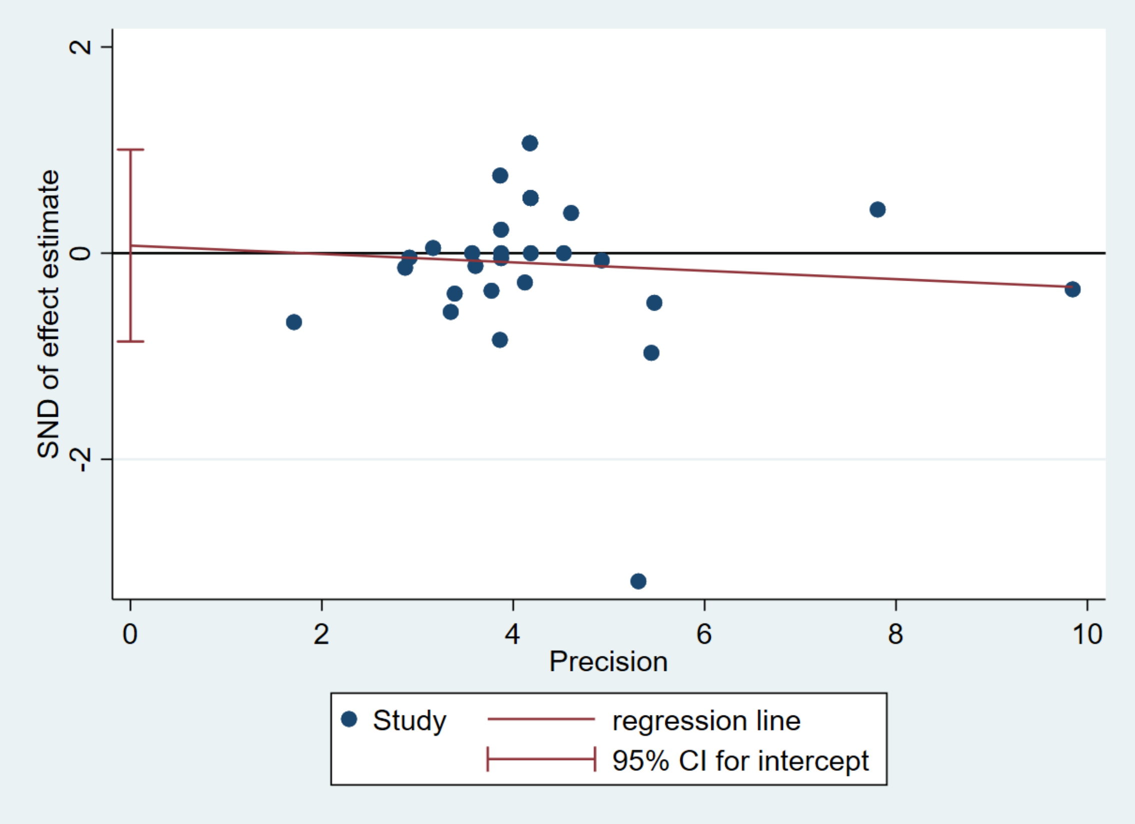

Supplementary materials

Cesarean Section Rate

```
Number of studies =  14                              Root MSE        =  1.352
```

Std_Eff	Coef.	Std. Err.	t	P>|t|	[95% Conf.	Interval]
slope	-.2097379	.1494934	-1.40	0.186	-.5354559	.1159801
bias	.0260914	.6379564	0.04	0.968	-1.363896	1.416079

```
Test of H0: no small-study effects          P = 0.968
```

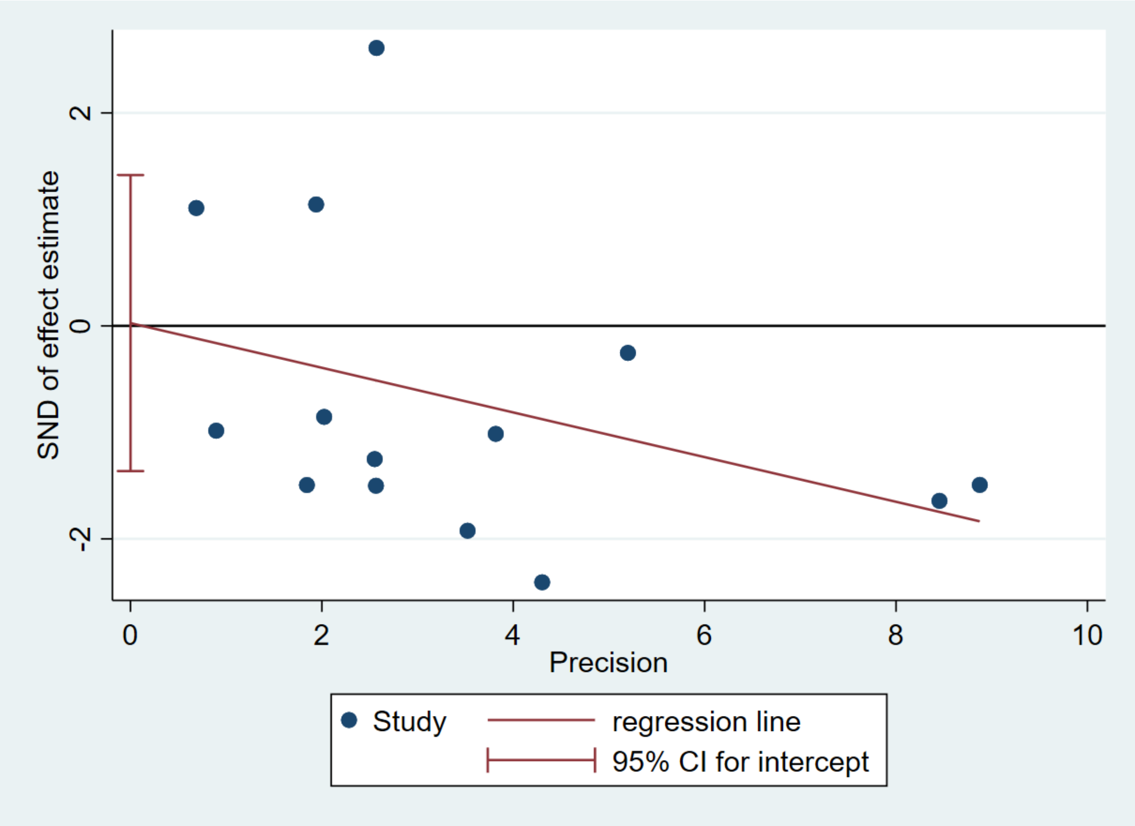

Supplementary materials

Figure S4: Subgroup analysis of fasting blood glucose outcomes in the GDM population

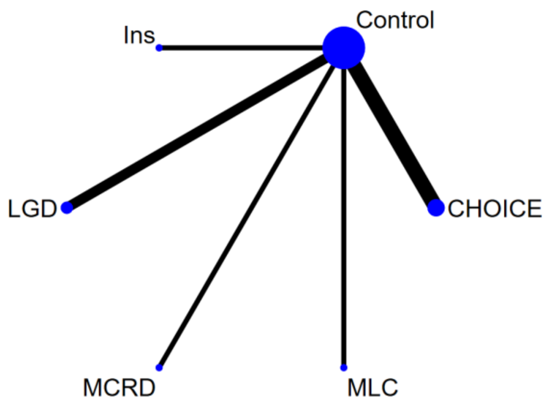

Figure S4.1. Network plot of subgroup analysis in the GDM population from high-income countries.

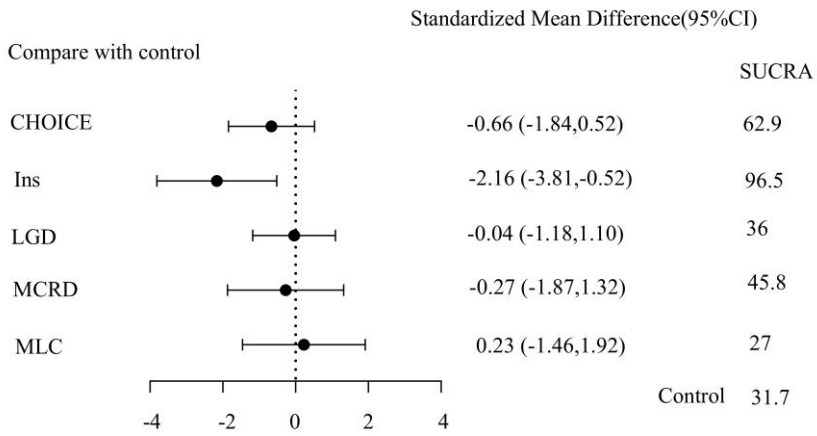

Figure S4.2. Forest plot of each intervention versus control and corresponding SUCRA values in the high-income country subgroup.

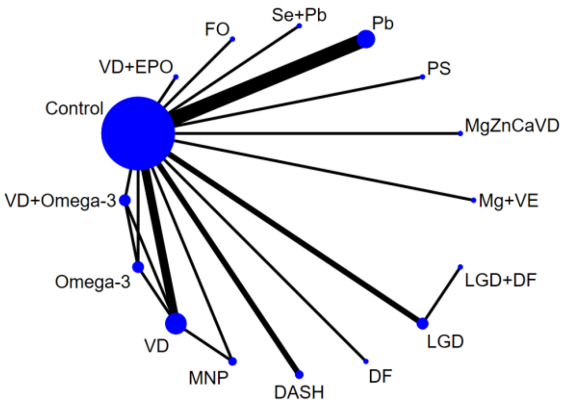

Figure S4.3. Network plot of subgroup analysis in the GDM population from low- and middle-income countries.

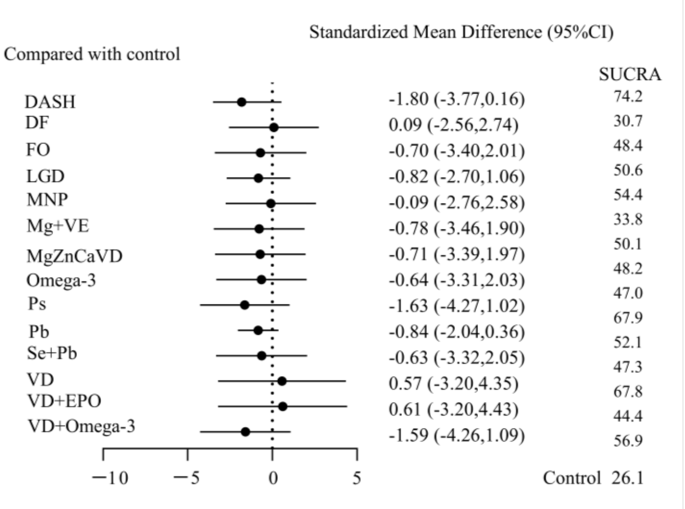

Figure S4.4. Forest plot of each intervention versus control and corresponding SUCRA values in low- and middle-income countries.

Supplementary materials

Figure S5: Sensitivity analysis results excluding high-risk studies

High-risk populations for GDM

Incidence of GDM

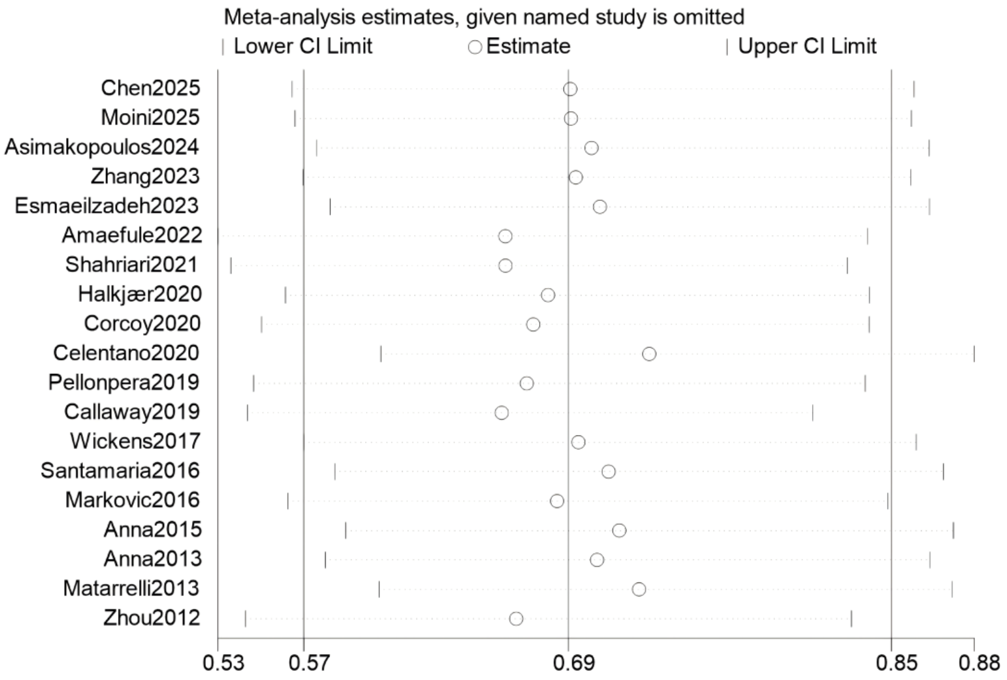

Cesarean section rate

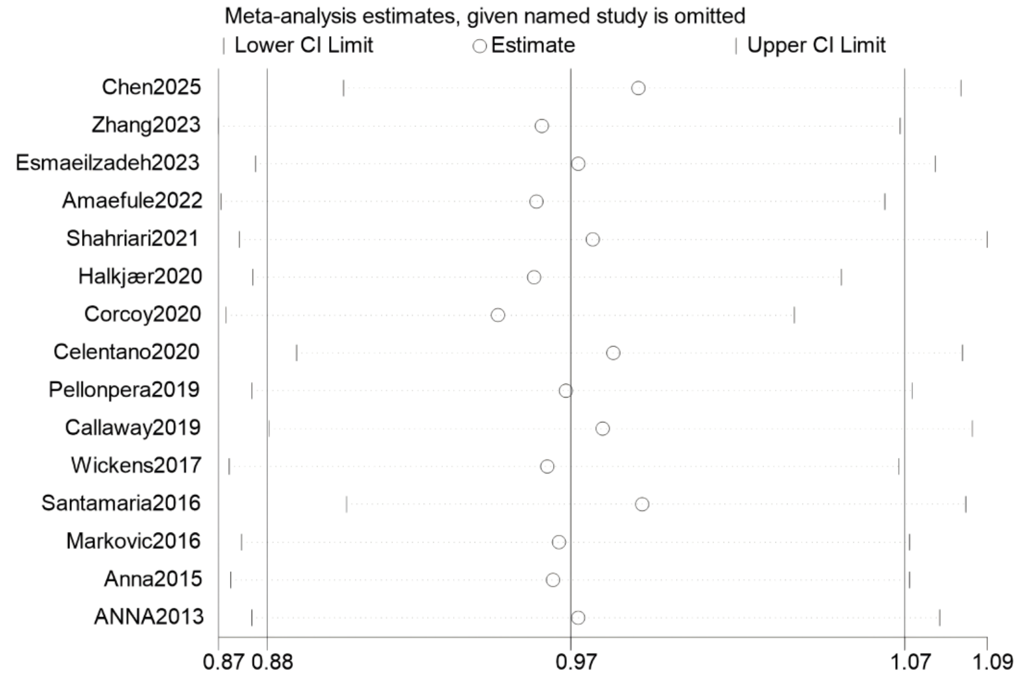

Supplementary materials

Gestational weight gain

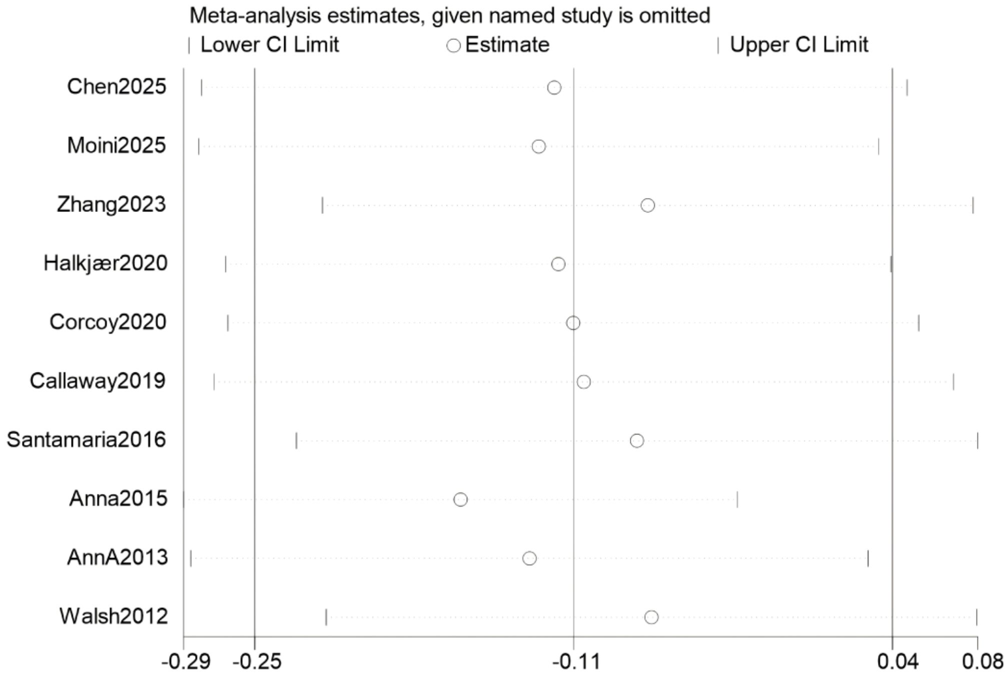

GDM patients

HOMA-IR

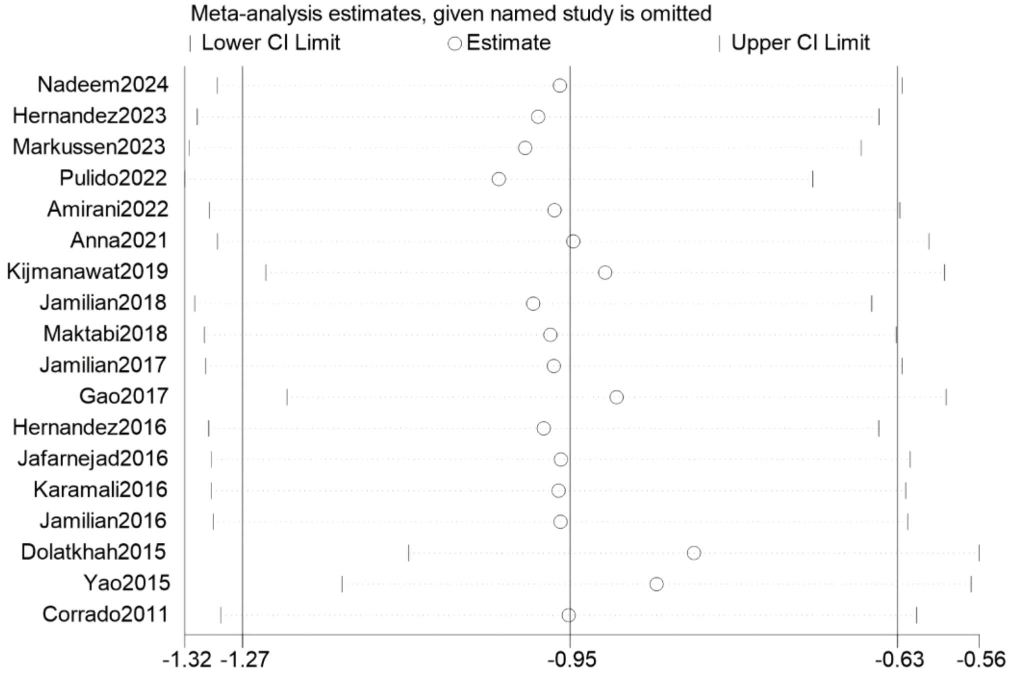

Supplementary materials

Cesarean section rate

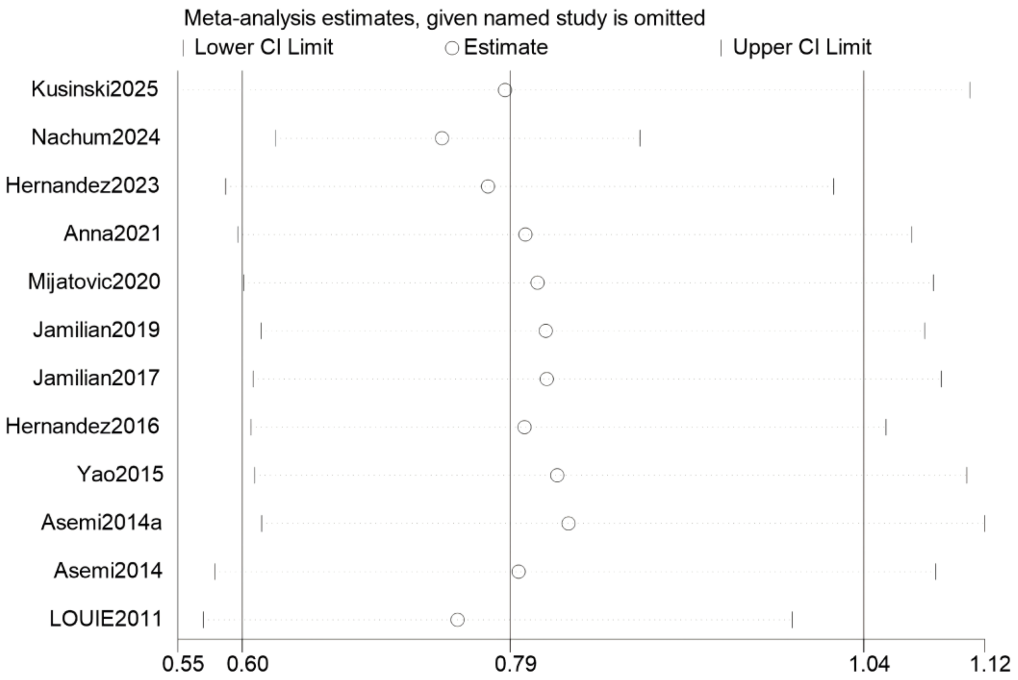

Fasting blood glucose

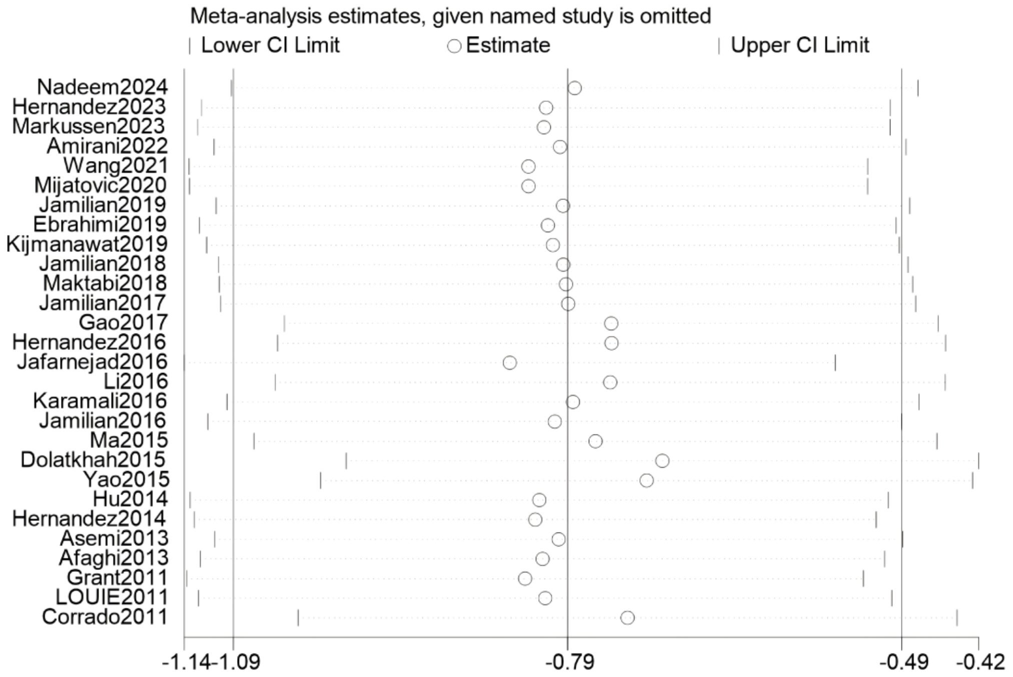

Supplementary materials

Gestational weight gain

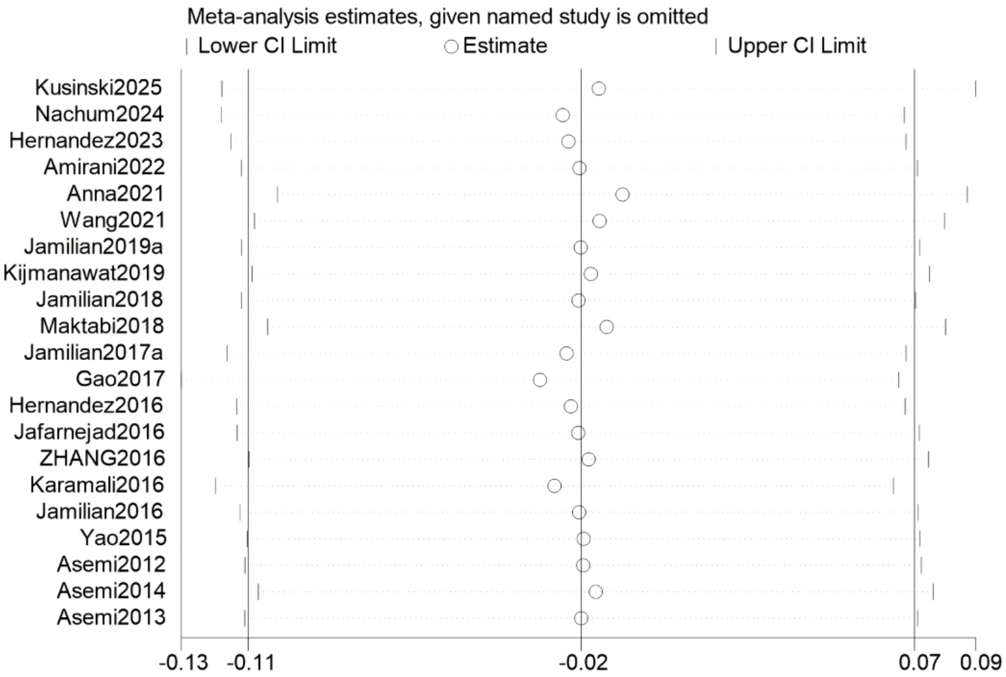

Supplement: Supplementary file 1 [file healthcare-14-02593-s001.zip › healthcare-4314206-supplementary.pdf]
